# A tunable family of CAAC-ruthenium olefin metathesis catalysts modularly derived from a large-scale produced ibuprofen intermediate†

**DOI:** 10.1039/d3sc03849a

**Published:** 2023-09-22

**Authors:** Adrian Sytniczuk, Filip Struzik, Karol Grela, Anna Kajetanowicz

**Affiliations:** a Biological and Chemical Research Centre, Faculty of Chemistry, University of Warsaw Żwirki i Wigury 101 02-089 Warsaw Poland a.kajetanowicz@uw.edu.pl

## Abstract

A series of tunable CAAC-based ruthenium benzylidene complexes with increased lipophilicity derived from a ketone being a large-scale produced key substrate for a popular nonsteroidal anti-inflammatory drug—ibuprofen was obtained and tested in various olefin metathesis transformations. As a group, these catalysts exhibited higher activity than their known analogues containing a smaller and less lipophilic phenyl substituent on the α-carbon atom, but in individual reactions, the size of the *N*-aryl moiety was revealed as a decisive factor. For example, in the cross-metathesis of methyl oleate with ethylene (ethenolysis)—a reaction with growing industrial potential—the best results were obtained when the *N*-aryl contained an isopropyl or *tert*-butyl substituent in the *ortho* position. At the same time, in the RCM, CM, and self-CM transformations involving larger olefinic substrates, the catalysts with smaller aryl-bearing CAAC ligands, where methyl and ethyl groups occupy *ortho*, *ortho*’ positions performed better. This offers a great deal of tunability and allows for selection of the best catalyst for a given reaction while keeping the general structure (and manufacturing method) of the ibuprofen-intermediate derived CAAC ligand the same.

## Abstract 2

A tunable family of ibuprofen intermediate-derived CAAC-base complexes for different applications, dependent from the size of the *N*-aryl substituent.

## Graphical abstract

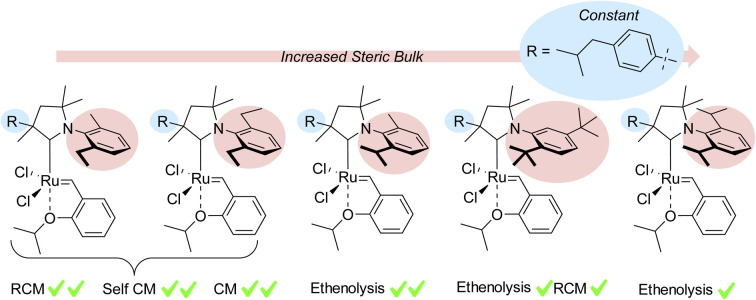

## Introduction

Olefin metathesis has attracted persistent interest from both academia and industry for many years, as it enables the synthesis of a wide range of useful organic compounds with carbon–carbon double bonds.^1,2^ This includes preparation of biologically active ingredients used in the pharmaceutical industry^3^ or fragrance compounds needed by F&F companies,^4^ but also transformation of raw materials of natural origin, such as rapeseed or palm oil, into materials used in the production of lubricants, plasticisers, or surfactants.^5^ Important milestones in the development of this methodology have been the introduction of well-defined ruthenium and molybdenum complexes,^6,7^ the application of N-heterocyclic carbenes (NHCs) as ligands to increase the stability of catalysts,^8–10^ the discovery of stereoselective^11–13^ and stereoretentive complexes^14,15^ providing the products with defined geometry of the double bond, and, more recently, the use of cyclic (alkyl)(amino)carbenes (CAACs). The latter were introduced by Bertrand^16^ and were further utilised by Grubbs^17^ and Pederson^18^ to synthesise the corresponding ruthenium catalysts. CAACs themselves are more nucleophilic and electrophilic than NHCs, as a result of the replacement of the π-donating and σ-attracting nitrogen atom in the latter by the σ-donating quaternary carbon atom. This change also alters the geometry around the metal centre, as the CAAC ligands create a kind of “wall”, while in the case of NHCs it is more an “umbrella” over the ruthenium atom.^19^ These factors may be responsible for the lower sensitivity to ethylene due to the higher stability of the ruthenacycle to β-elimination, but also their increased susceptibility to bimolecular decomposition.^20^

Initially, catalysts containing CAAC-type ligands were mainly used in ethenolysis, an important industrial reaction that converts, among others, vegetable oils rich in unsaturated fats into materials used in the production of, for example, cosmetics and household chemicals.^21^ Later, however, their applicability was extended to other types of olefin metathesis, including ring-closing metathesis (RCM), cross metathesis (CM), en-yne metathesis, and ring-opening metathesis polymerisation (ROMP).^19^

**Fig. 1:**
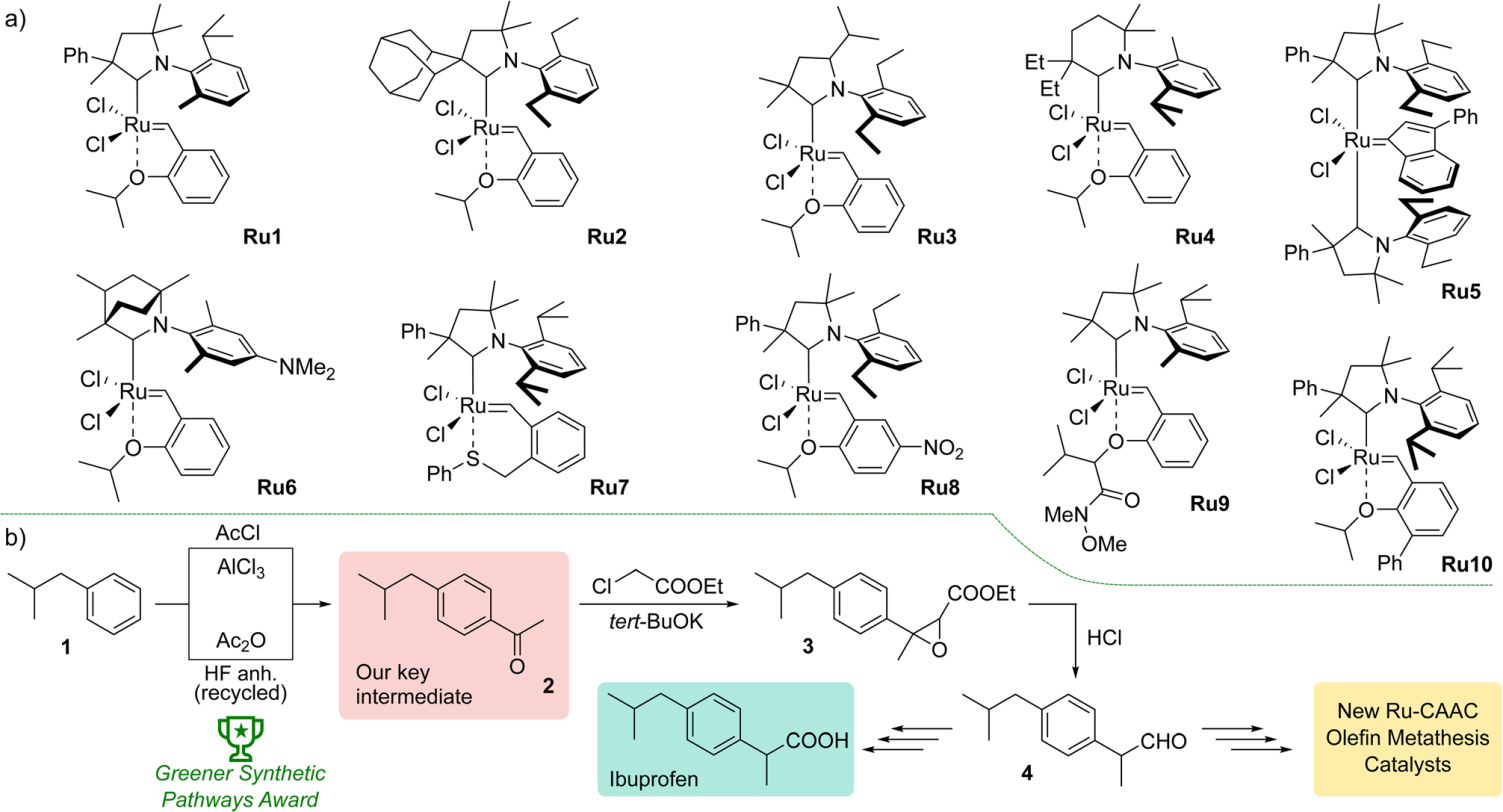
(a) Examples of CAAC-based ruthenium complexes showing diverse structural modifications of the CAAC ligand structure. (b) Upper path, original synthetic pathway to Ibuprofen by Boots UK Limited. Lower path, currently used Friedel–Crafts acylation optimised to meet *Green Chem.* principles.

The first promising results obtained with the new type of complexes triggered further research, including modification of the structure of aniline and substituents on the α- or γ-carbon,^22^ introduction of six-membered CAACs,^23^ obtaining complexes with enantiomerically pure ligands,^24^ modified with polar groups to increase water solubility^25^ or possessing CAAC with bicyclic structure increasing the thermal stability of the resulted catalyst (Fig. 1).^26^ The focus was also on changes within the benzylidene ligands, introducing additional substituents in the aromatic ring^27^ or modifying the structure of the chelating group.^28^ A series of indenylidene catalysts with two CAAC ligands were also obtained.^29^ Despite the diversity of structures of CAAC-based complexes, selected examples of which are shown in Fig. 1, there is a lack of systematic studies on the effect of the architecture of individual ligands on catalysts activity in different types of metathesis reactions. An exception is the study by Grubbs *et al.*,^21^ but here the authors focused exclusively on ethenolysis reactions.

To join this general trend towards the synthesis of more active and selective catalysts, we turned our attention to systems containing more lipophilic substituents on the α-carbon atom. Looking for inspiration, we came across the Ibuprofen synthesis developed in Boots UK Limited that uses 4-isobutylacetophenone (2) as the key intermediate.^30^ In the original Boots' synthesis ketone 2 reacts with chloroethylacetate to give epoxide 3, which—in a one-pot process of hydrolysis, decarboxylation and rearrangement—produces 2-(4-isobutylphenyl)propanal (4).^31^ Recently, a new protocol for Friedel–Crafts acylation optimised to meet *Green Chem.* principles was introduced by BHC Company (now BASF Corporation) leading to 2 with high atom economy. We believe that commercial mass-produced ketone 2 may be a convenient and inexpensive starting material for the synthesis of CAAC-type ligand precursors, in which the phenyl substituent on the α-carbon is decorated with a lipophilic isobutyl group.

## Results

**Scheme 1:**
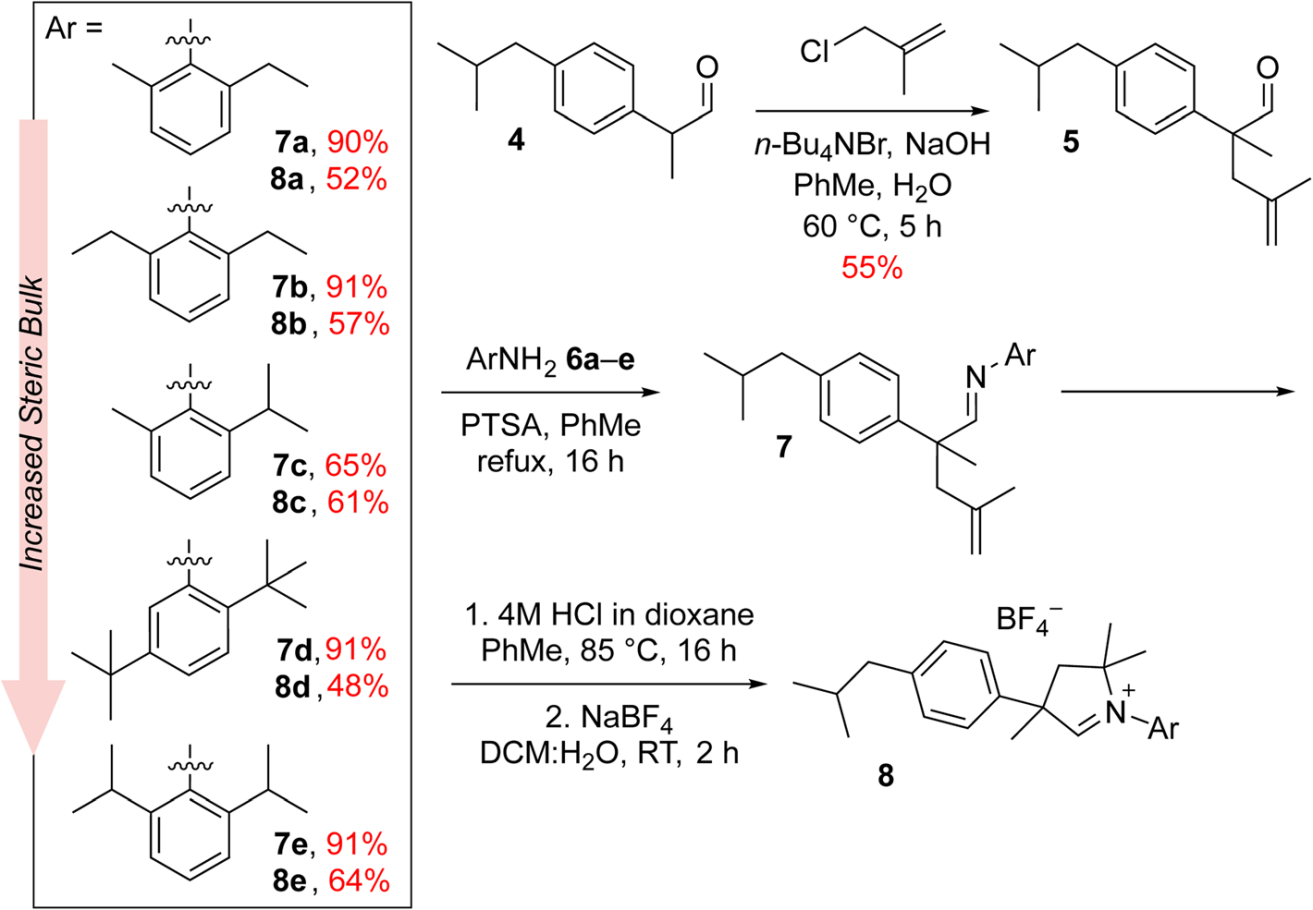
Synthesis of aldiminium salts 8a–e, being the CAAC ligand precursors.

The new aldiminium salts were synthesised according to a well-known literature procedure.^21^ First, we carried out the alkylation of 2-(4-isobutylphenyl)propanal (4) with 3-chloro-2-methylpropene under PTC conditions in the presence of tetrabutylammonium bromide and sodium hydroxide (Scheme 1). The resulting aldehyde 5 was then converted into a series of imines 7a–e by reaction with variously substituted anilines 6a–e in the presence of catalytic amount of *p*-toluenesulfonic acid. In all cases but for aniline 6c, the obtained yields were very good, reaching at least 90%. In the next step, we converted the imines 7a–e to the corresponding aldiminium salts 8a–e in a two-step procedure that first led to the closure of the corresponding chloride salt and then to the exchange of counterions from Cl^−^ to BF_4_^−^. Here, the yields were rather moderate; nevertheless, we were able to obtain sufficient amounts of aldiminium salts for further studies.

**Scheme 2:**
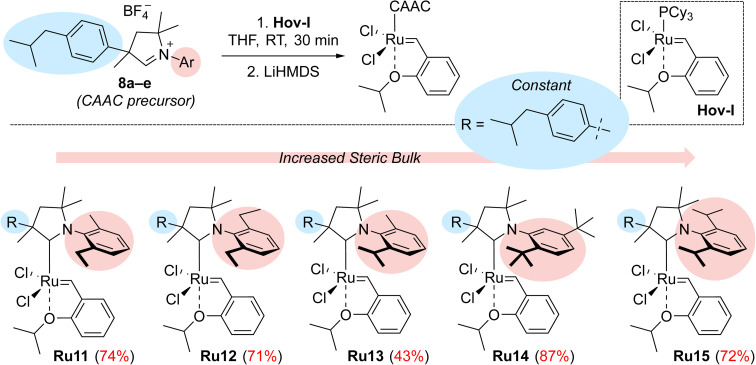
Synthesis of CAAC-based complexes Ru11–15. R = *p*-(Me_2_CHCH_2_)C_6_H_4_.

In subsequent reactions, we used the obtained aldiminium salts 8a–e to synthesise the desired catalysts (Scheme 2). To do so, we generated the corresponding carbenes *in situ* by means of LiHMDS in tetrahydrofuran at room temperature, which were treated with the Hoveyda–Grubbs first-generation complex (Hov-I). The expected Ru11–Ru15 catalysts were obtained as a result of PCy_3_ to CAAC ligands exchange; in most cases the yields exceeded 70–80%, which is a very good result for this type of catalysts.^21^ The newly obtained catalysts in a solid form are stable on air, and can be stored under typical conditions (+4 °C) for an extended period of time without loss of activity. Interestingly, in comparison with Ru1 the ibuprofen-intermediate derived catalysts are better soluble in nonpolar organic solvents such as *n*-hexane (Table 1); a property that we found potentially useful, as some metathetical transformations of fatty oils, waste α-olefins or other lipophilic substrates are best made in neat.

**Table 1:** Solubility of CAAC-based Hoveyda–Grubbs-type complexes Ru1 (benchmark) and Ru11–Ru15 in *n*-hexane at room temperature

Complex	Ru1	Ru11	Ru12	Ru13	Ru14	Ru15
Solubility [mg mL^−1^]	0.039	0.400	0.580	0.478	1.525	0.333

**Fig. 2:**
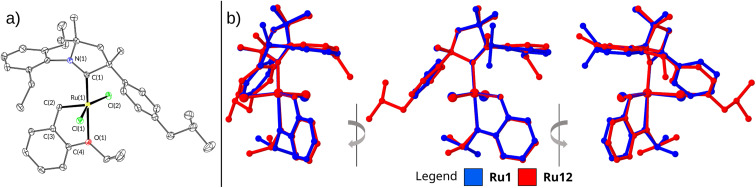
(a) Solid-state crystallographic structures of complexes Ru12. Hydrogen atoms removed for clarity. (b) Side view of molecule overlay of Ru1 and Ru12.

The structure and purity of all complexes were confirmed using standard analytical techniques, such as NMR, IR, HR-MS, and EA (for details, see ESI†). All complexes were isolated as green microcrystalline solids, yet we were able to grow a single crystal suitable for X-ray diffraction of only Ru12 (Fig. 2a and Table 2). The crystal grown *via* liquid to liquid diffusion of *n*-pentane into concentrated DCM solutions of the catalyst crystallised in the monoclinic *P*2_1_/*c* space group with one molecule of the compound in the asymmetric unit of the crystal lattice. The superimposition of Ru12 on Ru1 (the crystal structure taken from Grubbs' paper)^21^ shows no significant differences in the structure of these complexes (Fig. 2b). As expected, the Ru atom in both analysed complexes is pentacoordinated. The change in the carbene ligand in Ru12 compared to Ru1 has no significant effect on the length of the Ru–O bond. The values of this parameter are 2.3192(15) and 2.332(8) Å for Ru12 and Ru1, respectively. The Ru–C(1) (carbene atom in *N*-heterocycle) bond length for Ru12 and Ru1 also does not show a substantial difference and is equal to 1.936(2) and 1.940(7) Å for Ru12 and Ru1, respectively (Table 2). A comparison of the bond lengths between Ru and C(2) (carbene atom in benzylidene) also shows no significant differences. In both complexes these bond lengths are comparable: 1.840(2) and 1.836(9) Å, respectively. The values of the Ru–Cl bond lengths in Ru12 (2.3342(6) and 2.3543(6) Å) do not differ from these in Ru1 complex (2.3356(18) and 2.3271(13) Å). The angles between atoms C(1)–Ru–O(1) and C(1)–Ru–C(2) differ between these two complexes, although not significantly. Furthermore, no significant differences were observed in the values of dihedral angles, N(1)–Ru–C(1)–Cl(1) and N(1)–Ru-C(1)–Cl(2) (Table 2). Structural differences in the geometry of the coordination centre of Ru1 and Ru12 become more pronounced after the respective alignment of their molecules (Fig. 2b). The overlay of Ru1 and Ru12 depicted in Fig. 2 reveals that the molecular skeletons of these complexes exhibit different degree of deflection of the phenyl ring at the quaternary carbon atom in the plane of alkoxy benzylidene ligand.

**Table 2:** Selected bond lengths (Å) and angles (°) of complexes

	Ru1^a^^,^^b^	Ru12
Ru–C(1)	1.940(7)	1.936(2)
	1.931(12)	
Ru–O(1)	2.332(8)	2.3192(15)
	2.325(15)	
Ru–C(2)	1.836(9)	1.840(2)
	1.828(18)	
Ru–Cl(1)	2.3356(18)	2.3342(6)
	2.335(4)	
Ru–Cl(2)	2.3271(13)	2.3543(6)
	2.307(3)	
C(1)–Ru–O(1)	179.0(3)	176.13(8)
	177.7(5)	
C(1)–Ru–C(2)	102.9(3)	102.50(10)
	101.6(7)	
N(1)–Ru–C(1)–Cl(1)	−87.11	−83.21
	−125.91	
N(1)–Ru–C(1)–Cl(2)	116.60	117.20
	77.10	

a Data taken from ref. 21.

b Two molecules in the asymmetric cell unit.

**Fig. 3:**
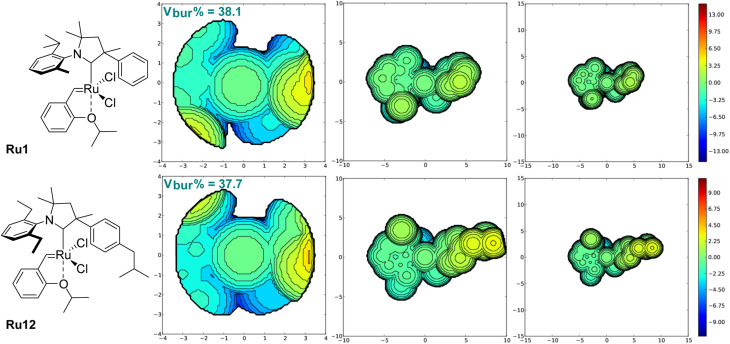
*V*_bur_% steric parameters and maps calculated in SambVca software for CAAC ligands in complexes Ru1 and Ru12. Left: Standard radii (3.5 Å), centre: enlarged radii (10 Å), right: enlarged radii (15 Å). The structures of the complexes (and not simply the ligands) are shown to enhance the readability.

To analyse closer the structure–activity relationship in the newly obtained complexes, we calculated the percent buried volume (*V*_bur_%) and topographic steric maps^32–34^ for CAAC ligands in benchmark Ru1 and in the new complex Ru12 (Fig. 3). As expected, due to the different substitution pattern in the aniline part, percent buried volume of CAAC in known Ru1 (38.1%) is greater than *V*_bur_% value of the CAAC ligands in newly obtained complex Ru12 (37.7%), although the latter contains rather a bulky Ph fragment with isobutyl substituent. Analysis of topographic steric maps shows that Ru centre in Ru1 is slightly more crowded at both R (Me, Ph) and Ar-sites compared to the newly obtained complex. This is caused by the twist of the *N*-aryl and phenyl substituents toward the ruthenium metallic centre visible on X-ray, whereas in Ru12 both substituents are twisted in a way that gives more space close to the metal centre. Moreover, it is clearly visible on Fig. 3 that, in the case of Ru12, there is a small cavity, which further decreases overall steric demand of the ligand.

The next logical step was to investigate the activity and selectivity of a set of new catalysts and compare them, where appropriate, with the benchmark catalysts.

### Activity of new complexes in ethenolysis

If one asked most organic or organometallic chemists which olefin metathesis reaction is most associated with the Ru-CAAC catalysts, the vast majority would probably indicate ethenolysis (cross-metathesis with ethylene) of methyl oleate (9). As already mentioned, it is the model reaction of CAAC-based complexes, which is related to their excellent ethylene tolerance, due to the high resistance of the ruthenacycle formed during the catalytic cycle to β-elimination.^20^ Ethenolysis of 9 was also our first choice, in which we tested Ru11–15, as well as complex Ru1 which is highly efficient in this type of processes,^21^ thus serving us as the benchmark (Table 3). The reaction conditions were adapted from the literature, allowing the best comparison to Ru1, however, we extended the reaction time to 6 h and—to make the process more user-friendly—prepared reagents, catalysts, and reaction setup outside of a glovebox.^21^ All reactions were conducted for 6 h at 40 °C in a steel autoclave at ethylene pressure of 10 bar. At this point, quality of methyl oleate (9) should be commented briefly. From our earlier studies^35,36^ we know that the purity of the starting material is crucial to the efficiency of the process; however, taking into account high price of extra-pure 9 (>99%) used by Grubbs^21^ and others,^37^ in our study we decided to go for methyl oleate of 95% purity. In addition to the number of catalytic cycles leading to ethenolysis products (10 and 11), we also measured the selectivity of the reaction as the proportion of ethenolysis products to undesired side products 12 and 13 originating from the parasitic self-CM process according to the definition used by experts in the field.^21,29^ Other by-products were not detected. Finally, since ethenolysis technology is believed to be used on a large-scale in biorefineries, making economical production of bulk-chemicals, usually low catalysts loading—not more than a few dozen ppm (part per million)—is targeted in this reaction.^38,39^

**Table 3:** Ethenolysis of 9 (95% pure) using ethylene grade 3.5 and 4.5^a^

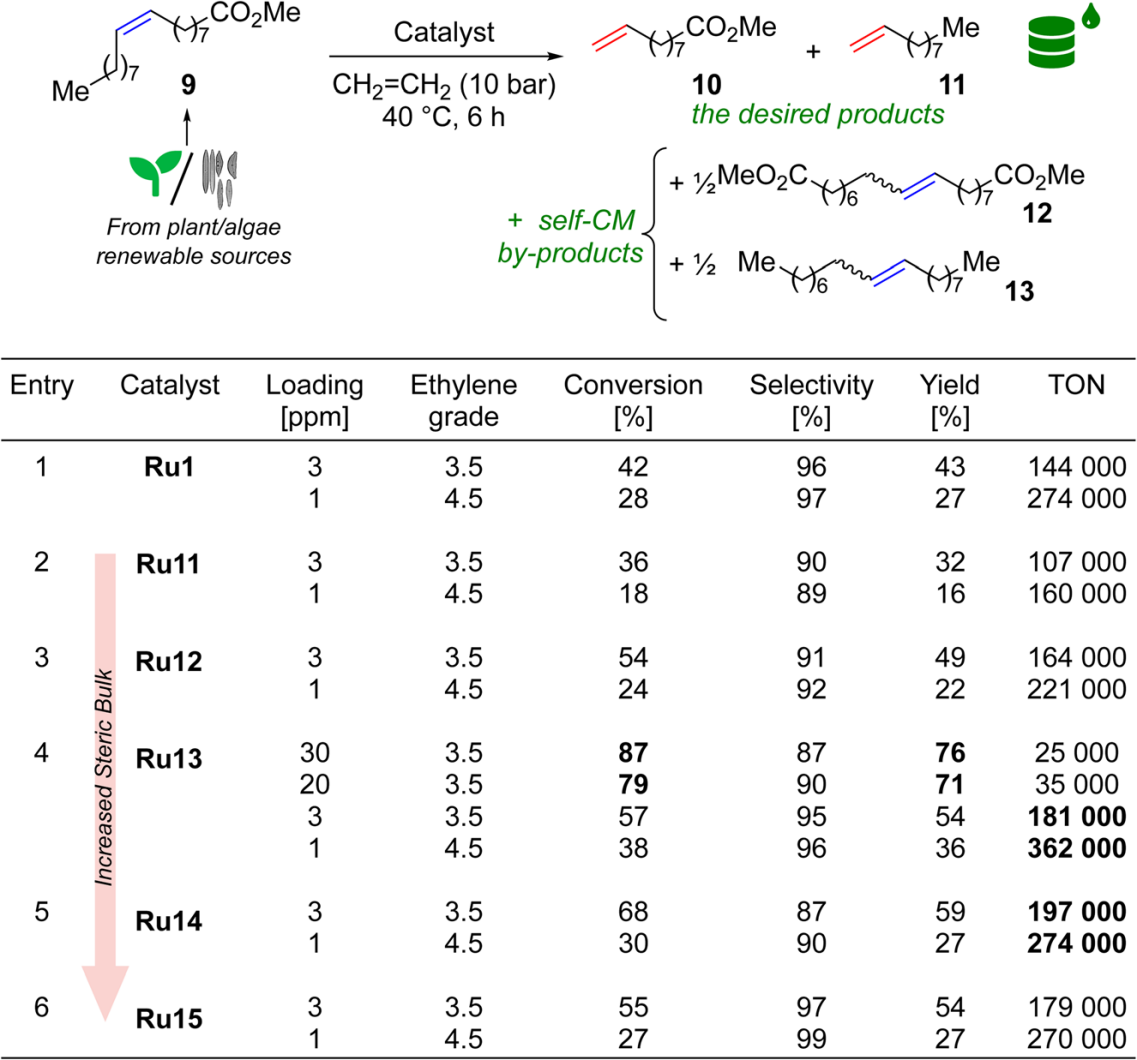

a Methyl oleate purity 95%, ethylene purity 99.95% (grade 3.5) or 99.995% (grade 4.5). Conversion = 100 × [1 − (*A*_9_ × *A*^0^_IS_)/(A^0^_9_ × *A*_IS_)]; selectivity = 100 × (*n*_10_ + *n*_11_)/[(*n*_10_ + *n*_11_) + 2 × (*n*_12_ + *n*_13_)]; yield = (conversion × selectivity)/100; TON = yield × [(*n*^0^_9_/*n*^0^_[Ru]_)]/100; *A*_9_, *A*_IS_ = GC area of methyl oleate and internal standard at the end of the reaction; *A*^0^_9_, *A*^0^_IS_ = GC area of methyl oleate and internal standard before the reaction. IS = internal standard (methyl stearate).

To gain a better insight into the influence of the catalyst structure on the results obtained, we first analysed a series of complexes that differ in the size of the substituents in the 2 and 6 positions of aniline, namely Ru11, Ru12, Ru13, and Ru15. Remarkably, at a catalyst loading of 3 ppm all complexes but Ru11, surpassed a TON of 140 000, and their activity increased in the following sequence of substituents: 2-Et-6-Me (Ru11) < 2,6-di(Et) (Ru12) < 2,6-di(i-Pr) (Ru15) < 2-i-Pr-6-Me (Ru13) (Table 3, entries 2–4,6), which is consistent with previous observations by Grubbs and Bertrand.^21^ and confirms the authors' hypothesis that at least one of the substituents in the *ortho* position of the nitrogen-bound aromatic ring should be sterically hindered. The hypothesis was strengthened when Ru14 complex—which has a large *tert*-butyl substituent at position 2 and no substituent at position 6—was included in the study and which showed even better activity in ethenolysis of 9 (Table 3, entry 5). On the other hand, it also appears that to obtain high selectivity toward ethenolysis products 10 and 11 instead of self-metathesis by-products 12 and 13, a substituent at position 6 (*e.g.* Me, i-Pr) in addition to a large substituent at position 2 (*e.g.* i-Pr) is necessary, as the best results were obtained for Ru13 and Ru15. For reactions carried out at a catalyst loading of 1 ppm, the trends for both activity and selectivity were preserved. Furthermore, to our delight, the results obtained in the reaction catalysed by Ru13, regardless of catalyst loading (yield 54%, TON 181 000 for 3 ppm, and 36%, TON 362 000), outperformed its closest analogue Ru1, differing by the aromatic substituent on the α-carbon (Ph in Ru1*versus* 4-*iso*-butylphenyl in Ru13, Table 3, entries 1 and 4), which justifies the search for new structural modifications.

Although it is currently difficult to predict at what conversion levels the future ethenolysis-based biorefineries will operate in practice, it seemed interesting to check the Ru loading necessary to obtain a high conversion in ethenolysis. To do so, we repeated the ethenolysis reaction of neat 9 using Ru13, the best catalyst selected previously, to find that 20–30 ppm are enough to secure 80–87% conversion (70–79% yield) in this reaction (Table 3, entry 4).^40^ It is worth mentioning that these results are in line with recently published activities reported for sterically-activated catalyst Ru10 bearing a CAAC ligand with (Ph, Me)-substituents at C(2) carbon and di(i-Pr)phenyl aniline fragment.^37^ This catalyst in ethenolysis of neat 9 (99% pure) gave 36% yield, TON 3600 at 100 ppm loading; 10% of yield, TON 10 000 at 10 ppm; and 8% yield, TON 16 000 at 5 ppm. Interestingly, it was possible to reach full conversion (97% of yield), however, using 9 as 0.02 M solution in toluene and 1000 ppm (0.1 mol%) of Ru10.^37^ However direct comparison between Mauduit and Bertrand's and our system meaningless due to different conditions used.^37^

### Activity of new complexes in self-CM reactions

**Scheme 3:**
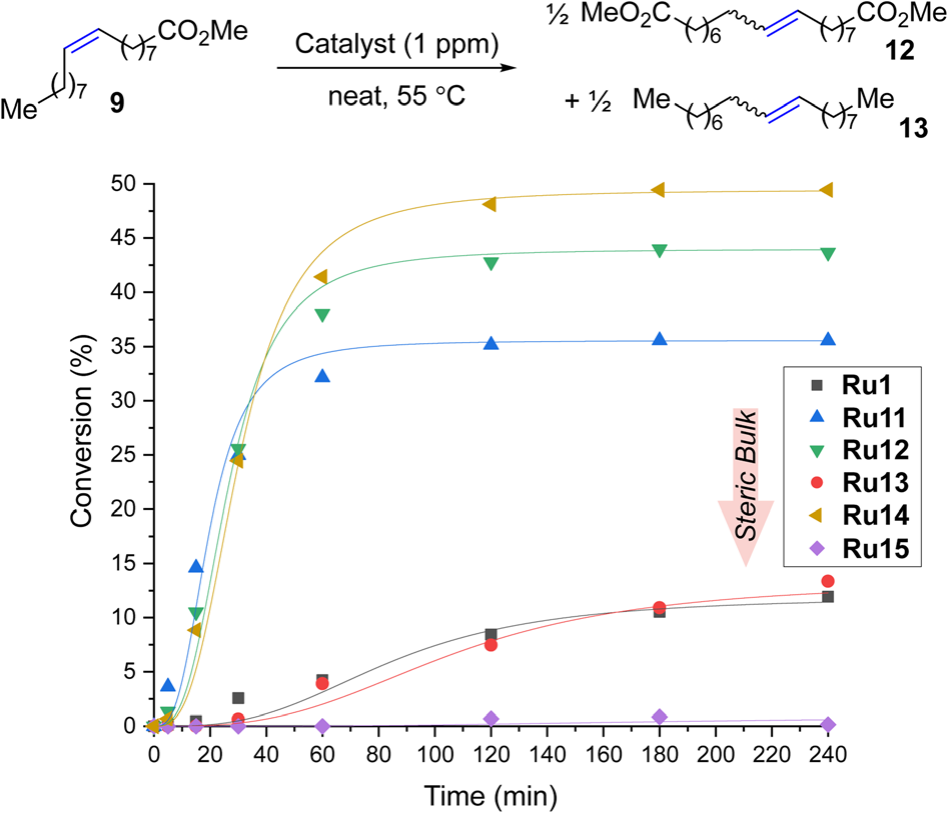
Self-metathesis of methyl oleate (9, 95% pure). Conversion = 100 × [1 − (*A*_9_ × *A*^0^_IS_)/(*A*^0^_9_ × *A*_IS_)]; *A*^0^_9_, *A*^0^_IS_ = GC area of methyl oleate and internal standard before the reaction. Lines are visual aids only.

To expand the applicability of 4-isobutylphenyl-based complexes to new types of metathesis reactions, we turned our attention to the self-metathesis of methyl oleate (9) leading to valuable diester 12 (Scheme 3). In this case, it is important to keep in mind that because the internal olefin is being used as the only substrate (and no volatile product such as ethylene is being removed from the reaction mixture) this transformation is an equilibrium process, so the maximal theoretical conversion achievable in this case is 50%.^23,41,42^

The reactions were performed in the presence of 1 ppm catalyst in neat at 55 °C. We were pleased to find that in this process Ru14 provided maximum conversion after only three hours, which, to the best of our knowledge, makes it the most active CAAC-based catalyst for the self-metathesis of methyl oleate (9) (so far, the best was Ru5 developed by Skowerski *et al.* giving a conversion of 45% at a loading of 5 ppm).^29^ When the same reaction was performed in the presence of 0.5 ppm of Ru14 after 4 hours at 55 °C 45% of conversion of 9 was observed, which corresponds to TON equal to 450 000.^43^ To our best knowledge, it is the first example of self-CM reaction of 9 performed at part-per-billion level published in scientific literature. The second best catalyst Ru12 was at 1 ppm loading 8 percentage points worse, while the other complexes were even less productive, and the most crowded Ru15 did not produce even a trace of the products. It is important to stress that, as was in the case of ethenolysis reaction, the production of 12, a commodity chemical, requires the use of the smallest possible loading of the noble metal catalyst, to make it economically^44^ Increasing the loading to 2.5 ppm improved the results for most catalysts (for example, Ru12 achieved a conversion of 50% and Ru11 was only 3 percentage points worse, for details, see ESI†), but the most crowded Ru15 remained inert in this reaction also at higher loading.

**Scheme 4:**
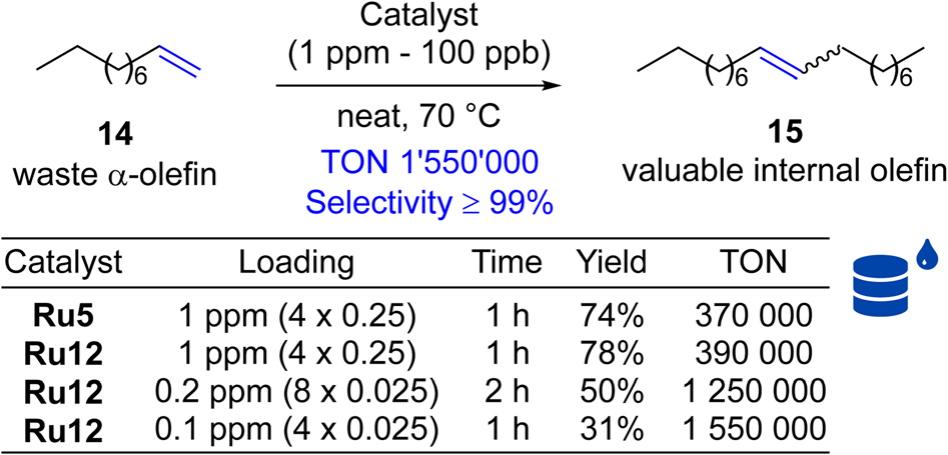
Self-metathesis of 1-decene (14, 96% pure). TON = 0.5 × yield × [(*n*^0^_14_/*n*^0^_[Ru]_)]/100.^29^ Where: *n*^0^_14_, *n*^0^_[Ru]_ = initial moles of 1-decene and catalyst used.

Next, self-CM reaction of α-olefins was selected, because of its potential high industrial importance, allowing for the transformation of low-value Fischer–Tropsch feedstocks into added-value chemicals. The metathetical “dimerisation” (self-CM, Scheme 4) of the abundant linear α-olefins present in Fischer–Tropsch fractions (30 to 70%) that are composed of C5 to C10 olefins offers an attractive direction, and was studied before.^45^ Unfortunately, this initial study has shown that several key issues must be addressed to make this technology applicable at large-scale industrial production. First, the catalyst should be robust and exhibit high activity at low loadings (ppm level or below). Second, this high catalytic activity must be associated with an extremely high selectivity towards the desired product, and elimination of unwanted processes such as C–C double bond migrations through the alkene chain of the linear olefinic substrate or product (“isomerisation”).^46–50^ Using 1-decene as the model α-olefin, we decided to test the performance of our new catalysts being used at extremely low loadings (Scheme 4). Here, we tested Ru12, the second best catalyst selected in self-CM of 9 (see above), because this complex was synthesised from more available and less expensive 2,6-diethylaniline (according to Sigma-Aldrich web page 2,5-di-*tert*-butylaniline is almost 40 times more expensive than 2,6-diethylaniline, which substantially increases the cost). It is also the closest analogue of Apeiron's Ru5 used previously in this process. As a result, we observed that even one part per million of this catalysts can convert 78% of substrate 14 into internal olefin 15. Of similar importance, we noted that selectivity in this reaction was ≥99% (no isomerisation products were observed). To explore the potential of further decreasing the amount of catalyst used, we decreased the loading of Ru12 to part per billion levels and recorded up to 1 550 000 productive turn-over cycles (TON)^29,51^ before the catalyst molecule was deactivated.

### Activity of new complexes in CM reactions

Taking a step further, we investigated the cross metathesis reaction between allylbenzene (16) and *Z*-1,4-diacetoxy-2-butene (17) which is a prototypical CM transformation, suggested by Grubbs to be used as a standard benchmark reaction (typically with 1 mol% of Ru catalyst).^52^ Following this general guidance^52^ the CM reaction was carried out in toluene at 55 °C (Table 4) using, however, a much reduced loading of CAAC-catalysts (250 ppm).

**Table 4:** Cross metathesis of allylbenzene (16) and *Z*-1,4-diacetoxy-2-butene (17)

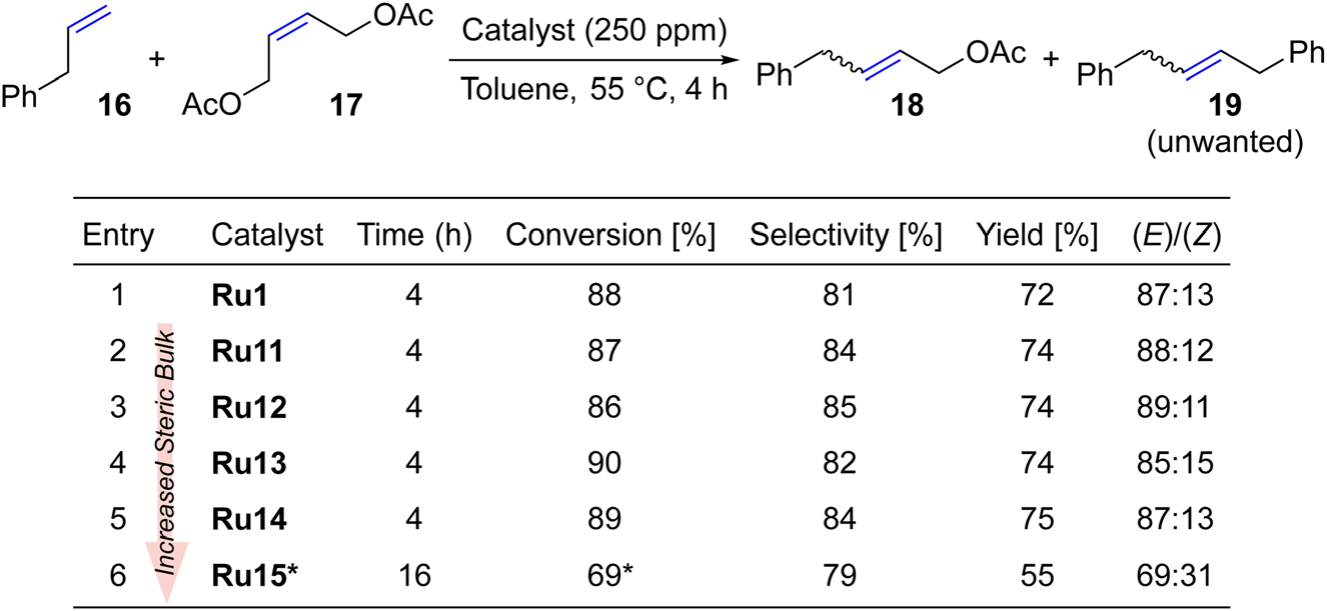

a Additional 100 ppm added after 4 h.

To our satisfaction, virtually all complexes led to a conversion close to 90% (up to 75% yield) after only 4 hours and in the presence of as little as 250 ppm of catalyst. In all the cases, the yields were around 10% lower than the conversions, as allylbenzene dimer 19 was formed as a side product (importantly, no C–C double bond migration was noted neither in 18 nor 19). Interestingly, the newly disclosed sterically activated CAAC catalyst Ru10 gave, in the same reaction, a 62% yield at a much higher loading of 5000 ppm (0.5 mol%).^37^ Also, in the case of cross metathesis between 16 and 17 the least active complex was the most hindered one, namely Ru15 possessing two isopropyl *ortho*-substituents in the aniline moiety. However, even this complex gave a moderately satisfactory conversion of 69% which was reached after 16 hours, with an additional portion of catalyst added to the reaction mixture. A relatively high contribution of the *E*-isomer of 4-phenylbut-2-en-1-yl acetate (18) was observed for all complexes except Ru15, which is typical for complexes with NHC^52^ rather than CAAC ligands.^53^ On the other hand, Grubbs^53^ observed that more active catalysts with CAAC ligands at high conversions yield more *E*-product, which may suggest that also here thermodynamic factors play more important role than inherent properties of the catalysts.

Encouraged by the results obtained in the cross-metathesis reaction of allylbenzene (16) and *Z*-1,4-diacetoxy-2-butene (17), we decided to investigate the activity of our catalysts in reaction with a challenging electron-deficient olefin—acrylonitrile (21)—an CM partner that typically requires 5–8 mol% catalyst loading.^54–56^ This compound belongs to type III olefins (less reactive) according to the Grubbs classification,^57^ and was classified as a “poison” to Ru-based metathesis catalysts.^27^

**Scheme 5:**
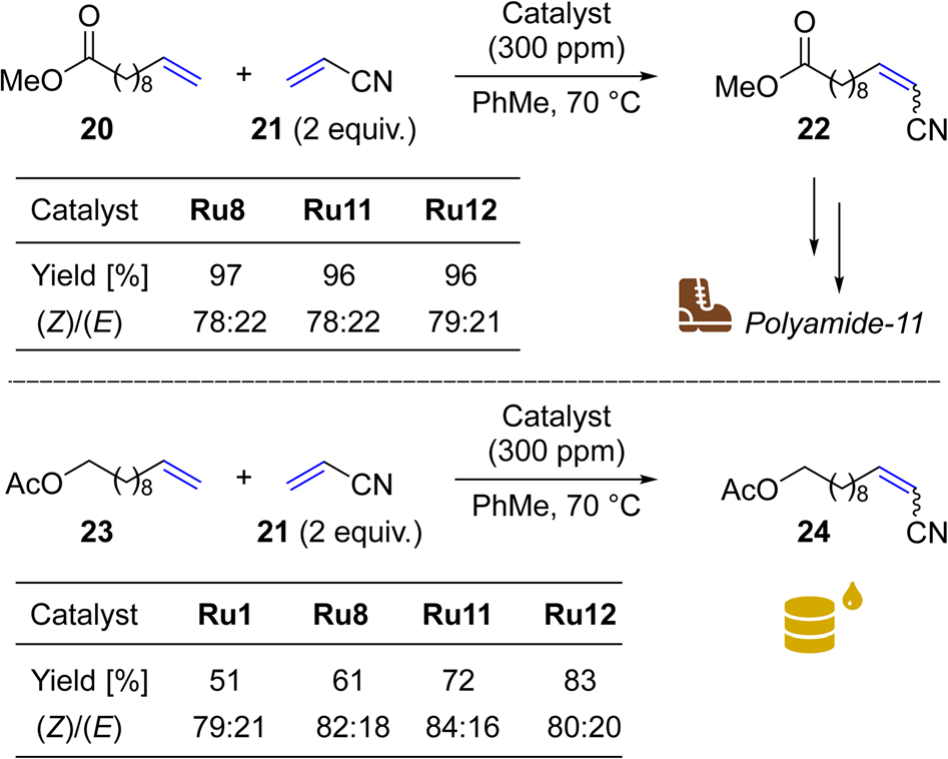
Cross metathesis between (a) 10-dodecenoic acid methyl ester (20) and (b) undec-10-en-1-yl acetate (23) and acrylonitrile (21, 2 equiv.).

To do so, we focus on a reaction leading to nitrile 22, a precursor of a valuable monomer used in Arkema's Nylon-11 (Rilsan® Polyamide 11) production.^58^ It should be noted that CM reactions of acrylonitrile (21) with 10-dodecenoic acid methyl ester (20) and other fatty acid esters were exhaustively studied by Bruneau in cooperation with scientists from Arkema.^59,60^ In this research use of at least 3 mol% (30 000 ppm) of Hoveyda–Grubbs NHC-containing catalysts was necessary to get high conversions of 20. In our study, two of the most active “small-CAAC” complexes (Ru11 and Ru12) were compared with the benchmark catalysts Ru1 and Ru8. As a result, we were pleased to see that with only 300 ppm of Ru11 and Ru12 it was possible to get 96–97% conversion in this industrially relevant reaction (Scheme 5, upper). This result places our new catalysts *ex aequo* with the nitro-activated CAAC complex Ru8 which was specially designed for CM with acrylonitrile.^27^

Next, we opted to test another olefinic substrate: undec-10-en-1-yl acetate (23) in CM with acrylonitrile (21), using the same conditions and set of catalysts (Scheme 5, lower). We were pleased to note that in this case the catalysts containing 4-isobutylphenyl moiety provided the desired product 24 in 83 (Ru12) and 72% (Ru11) isolated yields, a result superior not only to that obtained with the benchmark complex Ru1 (51%), but also to Ru8 (61%), recently commercialised by Apeiron-Synthesis.

### Activity of new complexes in RCM reactions

**Scheme 6:**
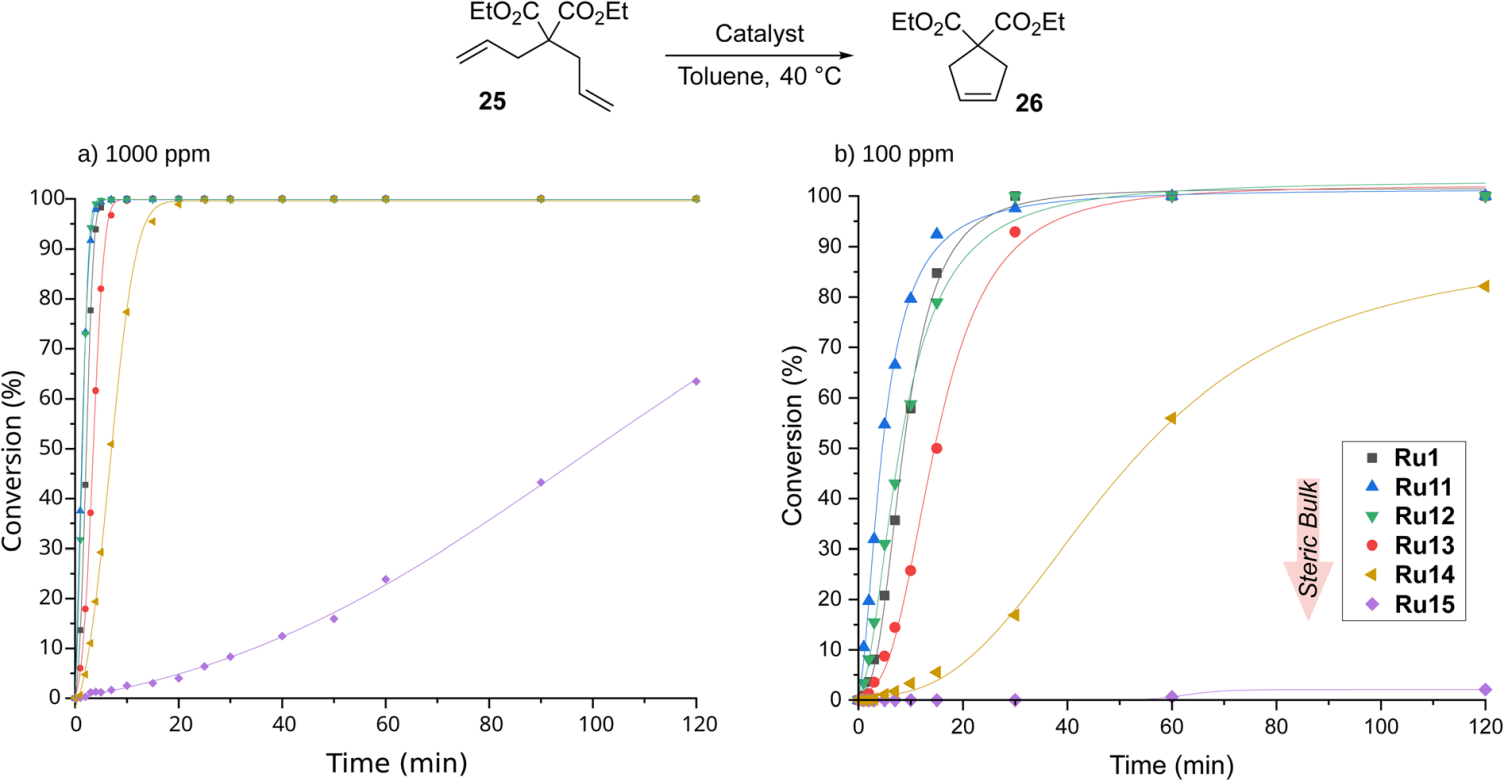
Time/conversion curves for the RCM reaction of diethyl 2,2-diallylmalonate (25, 99% pure) with 1000 ppm of CAAC-based complexes (monitored by GC). Lines are visual aids only.

CAAC-based complexes are mainly associated with the ethenolysis reaction, whereas other types of metathesis catalysed by them are much less explored.^19,61^ To fill this gap, we next turned our attention to ring-closing metathesis (RCM), a transformation that is frequently used in the context of medicinal chemistry^3,62,63^ and natural product synthesis.^64,65^ For RCM reactions tried in APIs (active pharmaceutical ingredients) production, ruthenium-based metathesis catalysts were typically applied in 0.1–2 mol%.^64^ One of the established model reactions to study cyclisation-effectivity of metathesis catalysts are the reactions of diethyl 2,2-diallylmalonate (25) and 2-allyl-2-(methylallyl)tosylate (27).^52^ RCM of 25 was carried out in toluene at 40 °C and with catalyst loading equal to 1000 ppm (0.1 mol%, Scheme 6), conditions similar to those applied previously by Gawin for reactions with CAAC-Hoveyda and bis(CAAC)Ru indenylidene complexes.^29^

The best catalyst described in the literature being an analogue of Ru1 with two methyl substituents on α-carbon reached full conversion after 30 minutes.^29^ To our delight, our complexes, excluding Ru15 containing the most sterically hindered substituent on nitrogen in the pyrrolidine ring, achieved the same result after just 20 minutes, and the best of them, namely Ru11 and Ru12, reached quantitative conversion in as little as 5 minutes (for details, see ESI†). Furthermore, the slowest of the complexes—Ru15—also accomplished full conversion, although in 6 hours. The high activity of the complexes studied prompted us to investigate their behaviour at 10 times lower loading, *i.e.*, 100 ppm. As expected, more time was needed to achieve full conversion; however, Ru11, Ru12, and the benchmark Ru1 achieved it in only 30 min, and Ru13 took twice as long. The two remaining complexes appeared much less active, with Ru14 giving 80% conversion after 2 hours and Ru15 almost completely dormant. Interestingly, in the RCM reaction the trend observed in ethenolysis was disrupted as catalysts containing relatively small substituents in the aniline-derived moiety performed best. It also appears that the smaller substituent on the α-carbon (phenyl instead of 4-isobutylphenyl) has a positive influence on the reaction rate (Ru1*versus*Ru13), although the differences are marginal.

**Scheme 7:**
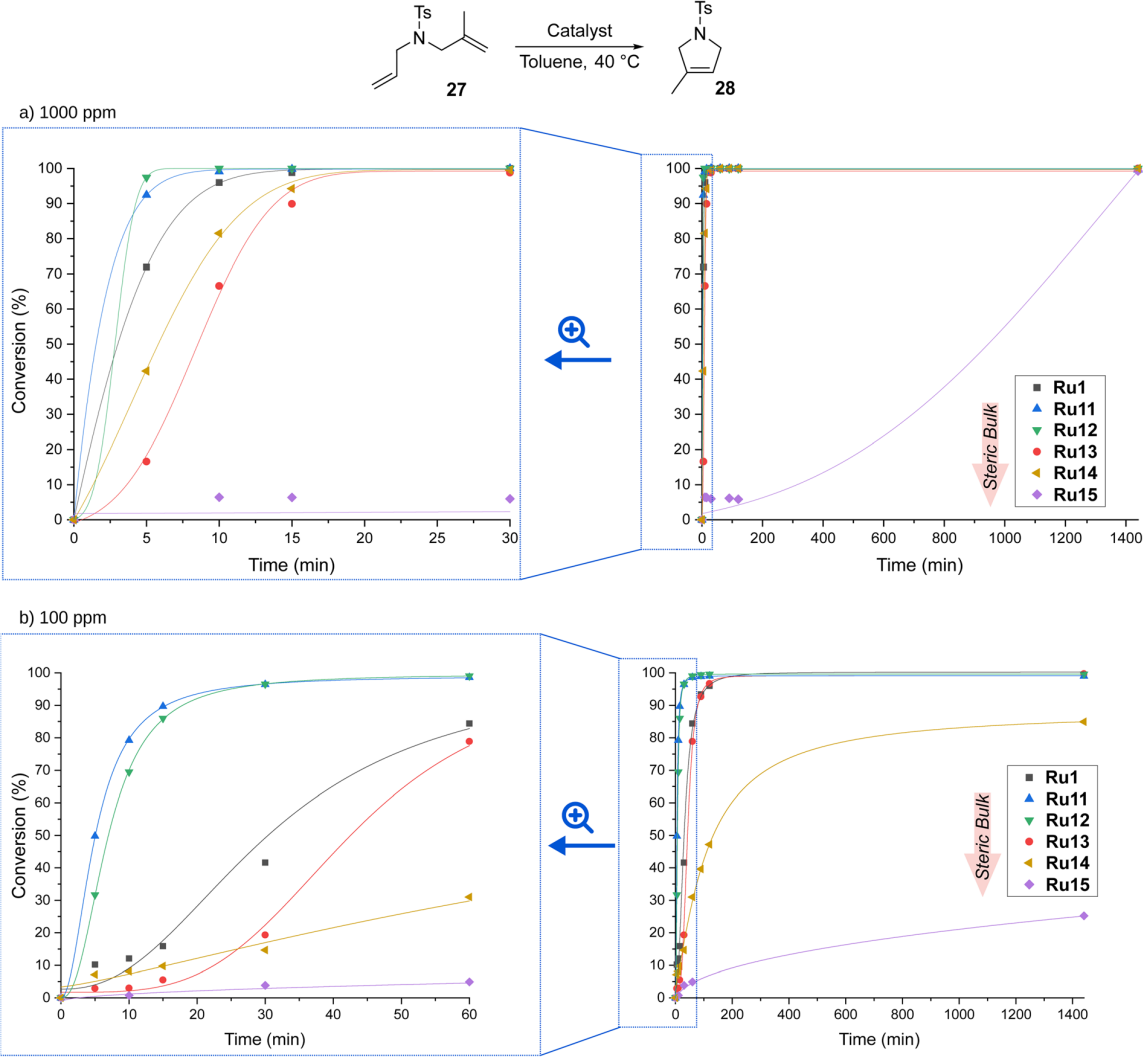
Time/conversion curves for the RCM reaction of 2-allyl-2-(methylallyl)tosylate (27, 96% pure) with (a) 1000 and (b) 100 ppm of CAAC-based complexes (monitored by GC). Lines are visual aids only.

Encouraged by the high activity of our complexes, we tested them in RCM of a more challenging substrate, 2-allyl-2-(methylallyl)tosylate (27), which reacts to form product 28 containing a trisubstituted double bond (Scheme 7).

At a catalyst loading of 1000 ppm, the trends observed for RCM of 25 were retained. Also here, the best performing complexes were those containing relatively small aromatic substituents on the nitrogen atom of pyrrolidine, *viz.*Ru11 and Ru12, which achieved full conversion after only 10 minutes; slightly worse, but still excellent results were obtained for Ru1, Ru14, and Ru13 (full conversion after about 20 minutes). Again, Ru15 decorated with two isopropyl substituents was found to be the least active, but even it fully transformed 27 into 28 after a sufficiently long time, *viz.* 24 hours. Decreasing the loading tenfold, to 100 ppm, in most cases did not affect the conversion, but only increased the time needed for its achievement, to 60 min (for Ru11 and Ru12) and 120 min (for Ru1 and Ru15). However, this time, the most sterically hindered complexes were not active enough and decomposed before converting all molecules of substrate 27 into the desired product 28, reaching 80 and 20% conversion after 24 hours for Ru14 and Ru15, respectively.

**Scheme 8:**
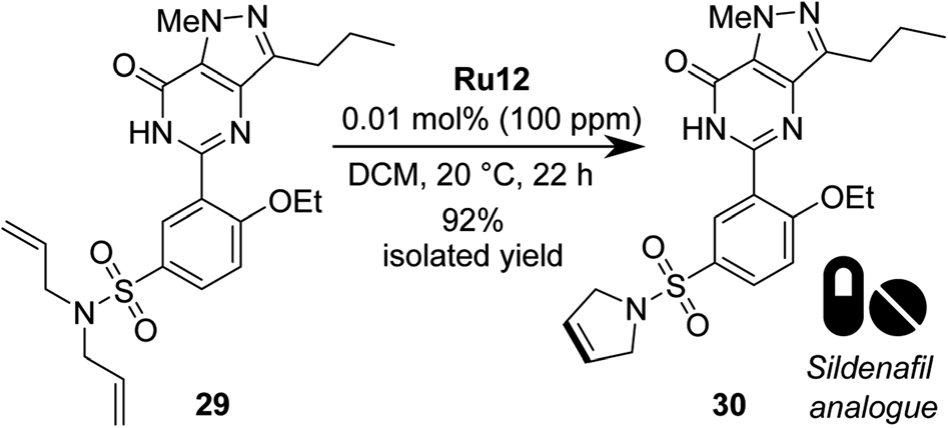
RCM reaction of Sildenafil analogue 29 (98% pure).

Finally, we decided to test the best catalyst, Ru12, in a more challenging RCM, using a polyfunctional substrate of medicinal chemistry interest, such as phosphodiesterase type 5 inhibitors (PDE5 inhibitors).^66^ To do so, we opted to apply the best RCM catalyst selected by us (*vide supra*) in the metathesis of Sildenafil^67,68^ (marketed *inter alia* under the brand name Viagra) analogue 29 (Scheme 8). From the point of view of catalytic olefin metathesis, such a substrate exhibits a potential risk, as it contains a number of Lewis basic centres that can chelate the propagating 14-e^−^ Ru species, thus arresting the catalyst's activity.^69–73^ To do so, RCM of 29 was conducted at RT in DCM for 22 h, giving product 30 in 95% isolated yield with only 0.01 mol% of catalyst Ru12, which was also the best one in RCM reactions with malonate models. Previously the same *N*,*N*-diallylsulfonamide substrate 29 was tested with NHC-bearing catalysts, that however were used in 2–0.5 mol% loading.

### Relative stability in solution as a function of *N*-aryl fragment of the CAAC ligand

**Fig. 4:**
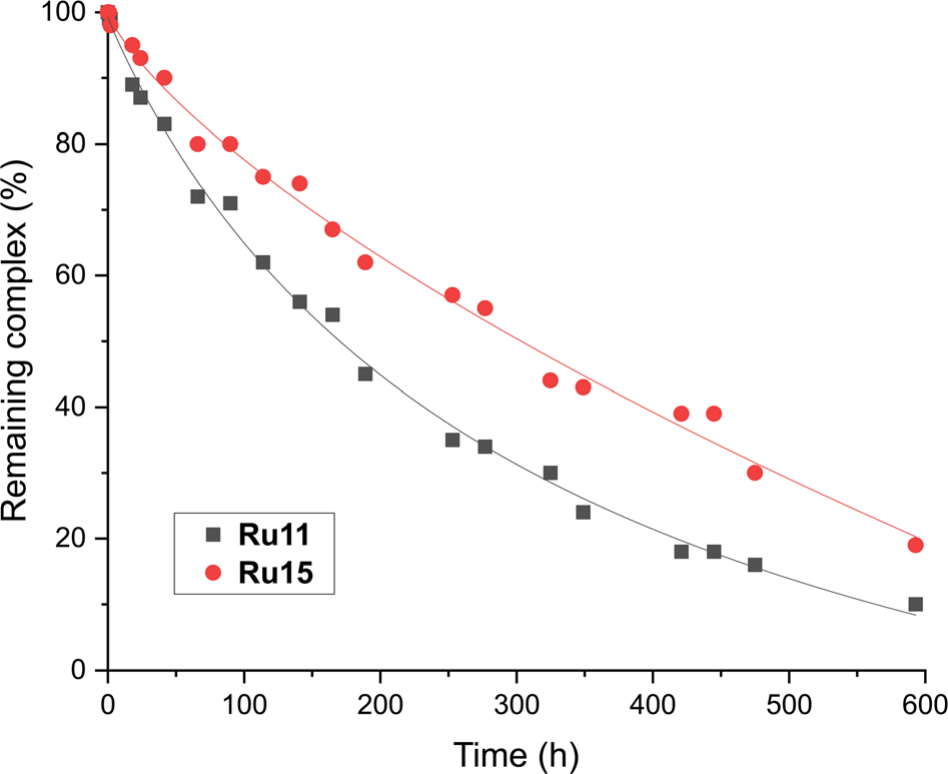
Relative stability of two representative CAAC-based ruthenium complexes (0.024 M solution in toluene-*d*_8_, at 80 °C, under air over 20 days). Lines are visual aids only.

From the chemical reactivity picture described above one can see that the new complexes (Ru11–Ru15), although very similar in their structures (identical benzylidene ligand and akin “ibuprofen intermediate-derived” CAAC ligands) exhibit different reactivity profiles in different types of olefin metathesis transformations. For example, “small-CAAC” complexes Ru11 and Ru12 were better in RCM of 16 and 18, while “larger-CAAC” Ru13 and Ru14 were better in ethenolysis. To check how the difference in the steric bulk exhibited by the “aniline fragment” (Ar) translates into the catalysts stability in a solution, relative decomposition rates of Ru11 and Ru15 in toluene-*d*_8_ solution under air atmosphere were measured (Fig. 4).^74^ Although in general the stability in solution of both complexes was very high (only ∼10% of decomposition after 2 days) under rather harsh conditions used (80 °C, under air), we observed a small but visible difference in stability that can be attributed to the steric bulk of the *N*-aryl part of the CAAC ligand. Namely, the bulkiest complex (2,6-di(i-Pr), Ru15) was visibly more stable than the one bearing less substituted Ar fragment (2-Et-6-Me, Ru11, Fig. 4), which harmonises well with noted lower catalytic activity of the latter.

## Conclusion

The replacement of the phenyl substituent with 4-isobutylphenyl (derived from a mass-produced ibuprofen intermediate) yielded a promising family of CAAC-Ru complexes whose stability and activity can be tuned by controlling the bulkiness of the *N*-aryl part of the ligand, thus broadening the spectrum of their applications (ethenolysis, RCM, self-CM, CM). Catalysts derived from relatively bulky anilines (Ru13 and Ru14) at a loading of only 1–3 ppm exhibit high activity in the CM of methyl oleate (11) with the smallest possible olefinic partner—ethylene. This result is of interest as the ethenolysis reaction is believed to gain industrial importance in the near future due to increased demands for use of biomass in production of sustainable chemicals with a reduced CO_2_-footprint. On the other hand, complexes bearing smaller *N*-aryl groups perform much better in RCM and CM reactions involving larger olefinic substrates, allowing for small loadings from 0.1 mol% to 0.2 ppm (200 ppb), depending on the reaction/substrate type. Therefore, while the relatively bulky *N*-aryl fragments in Ru13 and Ru14 make them very productive in ethenolysis of methyl oleate, the less bulky Ru11 and Ru12 react better with the bulkier substrates, such as the ones used in RCM and CM reactions. Interestingly, very bulky Ru15 failed in almost every transformation.

Maslow once stated “If your only tool is a hammer, then every problem looks like a nail”.^75^ Accordingly, we believe that the different substrate preferences exhibited by the new catalysts, which is related to the size of the substituents in the *N*-aryl part of the CAAC ligand, allow for a perfect fit between catalyst and substrate, and in more general terms opens interesting opportunities in fine-tuning of future generations of Ru olefin metathesis catalysts.

## Data availability

Data related to this paper will be deposited in Zenodo repository.

## Author contributions

Conceptualization, A. K., K. G.; investigation, A. S., F. S.; data analysis, A. S., F. S., A. K., K. G.; writing—original draft, A. K. and K. G. with input from all authors; writing—review and editing, A. S., F. S., A. K. K. G.; funding acquisition, A. K.

## Conflicts of interest

A. S., F. S., A. K., and K. G. are authors of Polish Patent Application, P.441535, which covers part of the results described in this paper.

## Supplementary Material

SC-014-D3SC03849A-s001

SC-014-D3SC03849A-s002
