# Supplementary material for: A tunable family of CAAC-ruthenium olefin metathesis catalysts modularly derived from a large-scale produced ibuprofen intermediate
Source: Chem Sci. 2023 Sep 22;14(39):10744–55. doi: 10.1039/d3sc03849a (PMC10566500; doi:10.1039/d3sc03849a)
Supplement: SC-014-D3SC03849A-s001 [file SC-014-D3SC03849A-s001.pdf]

Electronic Supplementary Material (ESI) for Chemical Science.
This journal is © The Royal Society of Chemistry 2023

Supporting information

A tunable family of CAAC-ruthenium olefin metathesis catalysts modularly derived from a large scale produced ibuprofen intermediate

Adrian Sytniczuk,[a] Filip Struzik,[a] Karol Grela,[a] and Anna Kajetanowicz[a,*]

Biological and Chemical Research Centre, Faculty of Chemistry, University of Warsaw
Żwirki i Wigury 101, 02-089 Warsaw, Poland

Email address: a.kajetanowicz@uw.edu.pl

Table of contents

1 Materials and methods 4

1.1 General remarks 4

1.2 Reagents and solvents 5

2 Synthesis of aldehyde 5 6

2.1 2-(4-isobutylphenyl)-2,4-dimethylpent-4-enal (**5**) 6

3 Synthesis of imines 7

3.1 General procedure for synthesis of imines 7

3.1.1 *N*-(2-ethyl-6-methylphenyl)-2-(4-isobutylphenyl)-2,4-dimethylpent-4-en-1-imine (**7a**) 7

3.1.2 *N*-(2,6-diethylphenyl)-2-(4-isobutylphenyl)-2,4-dimethylpent-4-en-1-imine (**7b**) 7

3.1.3 2-(4-isobutylphenyl)-*N*-(2-isopropyl-6-methylphenyl)-2,4-dimethylpent-4-en-1-imine (**7a**) ... 7

3.1.4 *N*-(2,5-di-*tert*-butylphenyl)-2-(4-isobutylphenyl)-2,4-dimethylpent-4-en-1-imine (**7e**) 8

3.1.5 *N*-(2,6-diisopropylphenyl)-2-(4-isobutylphenyl)-2,4-dimethylpent-4-en-1-imine (**7d**) 8

4 Synthesis of tetrafluoroborate CAACs precursors 9

4.1 General procedure for salts synthesis 9

4.1.1 1-(2-ethyl-6-methylphenyl)-4-(4-isobutylphenyl)-2,2,4-trimethyl-3,4-dihydro-2*H*-pyrrol-1-ium tetrafluoroborate (**8a**) 9

4.1.2 1-(2,6-diethylphenyl)-4-(4-isobutylphenyl)-2,2,4-trimethyl-3,4-dihydro-2*H*-pyrrol-1-ium tetrafluoroborate (**8b**) 10

4.1.3 4-(4-isobutylphenyl)-1-(2-isopropyl-6-methylphenyl)-2,2,4-trimethyl-3,4-dihydro-2*H*-pyrrol-1-ium tetrafluoroborate (**8c**) 10

4.1.4 1-(2,5-di-*tert*-butylphenyl)-4-(4-isobutylphenyl)-2,2,4-trimethyl-3,4-dihydro-2*H*-pyrrol-1-ium tetrafluoroborate (**8d**) 10

4.1.5 1-(2,6-diisopropylphenyl)-4-(4-isobutylphenyl)-2,2,4-trimethyl-3,4-dihydro-2*H*-pyrrol-1-ium tetrafluoroborate (**8e**) 11

5 Synthesis of ruthenium complexes 12

5.1 General procedure for ruthenium complexes synthesis 12

5.1.1 **Ru11** 12

5.1.2 **Ru12** 12

5.1.3 **Ru13** 13

5.1.4 **Ru14** 13

5.1.5 **Ru15** 14

6 Solubility of complexes in *n*-hexane 14

7 Ethenolysis of methyl oleate (9) 15

7.1 Preparation of **9** 15

7.2 General procedure for ethenolysis of **9** 15

7.3 Procedure for GC analysis of results 15

7.4 Results 16

8 SCM of methyl oleate (9) 17

8.1 General procedure for SCM of **9** 17

8.2 Results for 2.5 ppm of catalyst 17

8.3 Results for 1 ppm of catalyst 19

9 SCM of 1-decene (14) 20

9.1 General procedure for SCM of **14** 20

9.2 Results 20

10 CM of allyl benzene (16) and (*Z*)-but-2-ene-1,4-diyl diacetate (17) 21

10.1 General procedure for CM of **16** and **17** 21

10.2 Results 21

11 CM of undec-10-en-1-yl acetate (20) and acrylonitrile (21) 22

11.1 General procedure for CM of **20** and **21** 22

11.2 Results 22

12 CM of undec-10-en-1-yl acetate (23) and acrylonitrile (21) 23

12.1 General procedure for CM of **23** and **21** 23

12.2 Results 23

13 RCM of diethyl 2,2-diallylmalonate (25) 24

13.1 General procedure for RCM of **25** 24

13.2 Results for 1000 ppm of catalyst 24

13.3 Results for 100 ppm of catalyst 26

14 RCM of *N*-allyl-4-methyl-*N*-(2-methylallyl)benzenesulfonamide (27) 27

14.1 General procedure for RCM of **27** 27

14.2 Results for 1000 ppm of catalyst 27

14.3 Results for 100 ppm of catalyst 28

15 RCM of *N*,*N*-diallyl-4-ethoxy-3-(1-methyl-7-oxo-3-propyl-6,7-dihydro-1*H*-pyrazolo[4,3-*d*]pyrimidin-5-yl)benzenesulfonamide (29) 30

16 Stability test for Ru11 and Ru15 on air 31

17 XRD DATA 33

17.1 Crystal data and structure refinement details for **Ru1** and **Ru12** 33

17.2 Bond lengths for **Ru12** 34

17.3 X-Ray structure for **Ru12** 34

18 Copies of NMR spectra 35

19 References 47

1 Materials and methods

1.1 General remarks

All reactions required the exclusion of oxygen and moisture were carried out in dry glassware (kept overnight in an oven at 120 °C) with dry anhydrous solvents (purified using mBraun's SPS) under a dry and oxygen-free argon atmosphere using the Schlenk technique. The addition of dry solvents or reagents was carried out using argon flushed stainless steel cannulas and plastic syringes.

For spectroscopic and analytic characterizations, the following devices were used:

Analytical thin layer chromatography (TLC) was performed on Merck Silica gel 60 F254 precoated aluminum sheets. Components were visualized by observation under UV light (254 or 365 nm) or dyed by aqueous $KMnO_4$ or anisaldehyde reagent.

Flash column chromatography was performed using silica gel 60 (230–400 mesh), purchased from Merck.

GC chromatograms were recorded using a PerkinElmer Clarus 580 model or Shimadzu GC-2010 Pro. As capillary column, either IntertCap 5MS-Sil (Perkin) or SH-Rxi-5Sil MS (Shimadzu) column were employed with helium as carrier gas. GC conversions were determined based on the ratio of an internal standard (methyl stearate) present in the starting material (for ethenolysis and self-metathesis of methyl oleate) or external standard (1,3,5-trimethoxybenzene) added to substrate before reaction (for all other reactions).

^{1}H NMR spectra were recorded in $CDCl_3$ or CD_2Cl_2 at room temperature on Agilent Mercury spectrometer (400 MHz). The data were interpreted in first-order spectra. Chemical shifts δ are reported in parts per million (ppm) downfield from trimethylsilane as a reference to the residual solvent signal: chloroform (δ_H = 7.26 ppm), dichloromethane (δ_H = 5.32 ppm). The following abbreviations are used to indicate the signal multiplicity: s (singlet), d (doublet), t (triplet), q (quartet), sept (septet), dd (doublet of doublet), dt (doublet of triplet), ddd (doublet of doublet of doublet), etc., bs (broad signal), m (multiplet). Coupling constants (*J*) are given in Hz and refer to H,H-couplings.

^{13}C NMR spectra were recorded in $CDCl_3$ or CD_2Cl_2 at room temperature on Agilent Mercury spectrometer (400 MHz). Chemical shifts δ are reported in parts per million (ppm) as a reference to the residual solvent signal: chloroform (δ_C = 77.16 ppm, central line of the triplet), dichloromethane (δ_C = 53.84 ppm, central line of the quintet). If no coupling constants are given, the multiplicity refers to ^{1}H-decoupled spectra; otherwise, the coupling constants belong to heteroatoms.

High Resolution Mass Spectra (HRMS) were provided by analytical laboratory at the Institute of Organic Chemistry, PAS or at the Institute of Biochemistry and Biophysics, PAS.

Elemental Analyses (EA) were provided by the EA analytical laboratory at the Institute of Organic Chemistry, PAS.

IR spectra were recorded on a Perkin-Elmer Spectrum One FTIR spectrometer. Substances were applied as films, solids, or in solution. The obtained data were processed with the software MestReNova (ver. 14.2.1). The wavenumbers are given in cm^{-1}.

1.2 Reagents and solvents

All reagents were purchased from Sigma-Aldrich, Apeiron Synthesis, TCI, Fluorochem, Alfa Aesar, and POCH and used without further purification unless otherwise stated.

2-(4-isobutylphenyl)propionaldehyde,[1] diethyl diallylmalonate,[2] *N*-allyl-4-methyl-*N*-(2-methylallyl)benzenesulfonamide[3] were obtained according to literature procedures.

THF, which was used in "Synthesis of ruthenium complexes", and toluene, which was used for the addition of catalyst in olefin metathesis reactions, were distilled over sodium under argon atmosphere.

SnatchCat (1,4-bis(2-isocyanopropyl)piperazine) was used as a solution in dichloromethane (c = 10 mg/mL) in order to quench the olefin metathesis reactions.

Ethylene was purchased and used as received from Air Liquide (3.5, 99.95% purity) or from Linde (4.5, 99.995% purity).

2 Synthesis of aldehyde 5

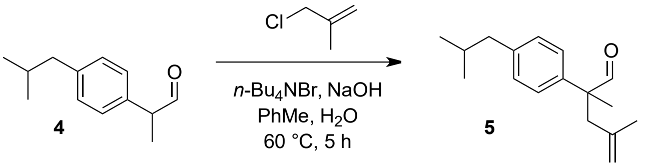

2.1 2-(4-isobutylphenyl)-2,4-dimethylpent-4-enal (**5**)

In a tree-neck round bottom flask equipped with a reflux condenser, dropping funnel, and magnetic stirring bar, NaOH (624 mg, 15.6 mmol, 1.50 equiv.), (*n*-Bu)$_4$NBr (335 mg, 1.04 mmol, 0.10 equiv.), distilled water (2 mL), and toluene (14 mL) were placed. The solution was warmed up to 60 °C and a mixture of **4** (1.98 g, 10.4 mmol, 1.00 equiv.) and 3-chloro-2-methylpropene (1.29 g, 13.8 mmol, 1.33 equiv., 97% pure) was added. The resulting solution was stirred at 60 °C for 3 hours. After cooling to RT, distilled water (20 mL) was added, layers were separated and aqueous layer was extracted with toluene (3×10 mL). The organic layers were combined, dried over Na_2SO_4, filtered, and the solvent was evaporated under reduced pressure. The residue was purified by distillation to afford the desired product as a colourless liquid (2.03 g, 8.31 mmol, 79%).

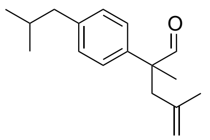

^{1}H NMR (400 MHz, $CDCl_3$): δ 9.54 (s, 1H), 7.24 – 7.12 (m, 4H), 4.82 (dq, *J* = 1.5, 2.1 Hz, 1H), 4.64 (dq, *J* = 0.9, 1.9 Hz, 1H), 2.77 – 2.60 (m, 2H), 2.48 (d, *J* = 7.2 Hz, 2H), 1.88 (sept, *J* = 6.8 Hz, 1H), 1.46 (s, 3H), 1.41 (dd, *J* = 0.8, 1.5 Hz, 3H), 0.91 (d, *J* = 6.7 Hz, 6H); **^{13}C NMR (101 MHz, $CDCl_3$):** δ 202.2, 141.8, 141.0, 137.0, 129.6, 127.2, 115.4, 53.3, 45.0, 44.3, 30.3, 24.2, 22.5, 18.6.; **HRMS-ESI (*m*/*z*)**: calculated for $C_{17}H_{25}O^+$ ($[M+H^+]^+$): 245.1900, found: 245.1902; **Elemental analysis**: calculated for $C_{17}H_{24}O$: C, 83.55; H, 9.90; found: C, 83.38; H, 9.79; **IR:** 3075, 3024, 2954, 2923, 2868, 2848, 2802, 2706, 1722, 1644, 1511, 1464, 1383, 1367, 1165, 1077, 1019, 894, 841, 795, 546.

3 Synthesis of imines

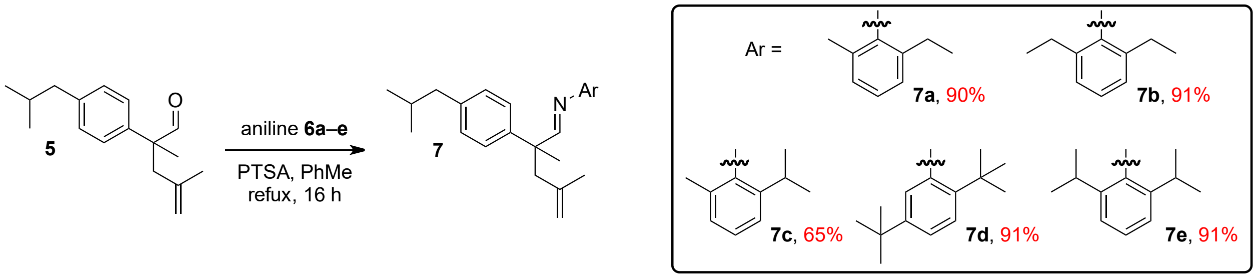

3.1 General procedure for synthesis of imines

In a round bottom flask equipped with Dean-Stark apparatus and magnetic stirring bar aldehyde **5** (1.00 equiv.), aniline (**6a**–**e**, 1.00 equiv.), and *p*-toluenesulfonic acid (PTSA, 1 mol%) were dissolved in toluene (c = 0.3 M). The reaction mixture was refluxed until the full consumption of the substrates (monitored by ^{1}H NMR measurements). The solvent was evaporated under reduced pressure, the crude reaction mixture was filtrated on short pad of neutral aluminum oxide (Al_2O_3, neutral, Broockman Grade I), and dried *in vacuo* to afford imines **7a**–**e** which were used in the next step without further purification.

3.1.1 *N*-(2-ethyl-6-methylphenyl)-2-(4-isobutylphenyl)-2,4-dimethylpent-4-en-1-imine (**7a**)

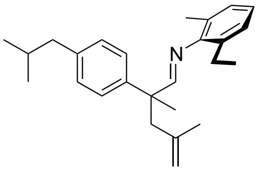

Imine was synthesized according to “General procedure for synthesis of imines”. **5** (1.50 g, 6.10 mmol), **6a** (830 mg, 6.10 mmol), and PTSA (11.0 mg, 61.0 µmol) were used to afford product **7a** as a yellow oily liquid (2.00 g, 5.53 mmol, 90%).

3.1.2 *N*-(2,6-diethylphenyl)-2-(4-isobutylphenyl)-2,4-dimethylpent-4-en-1-imine (**7b**)

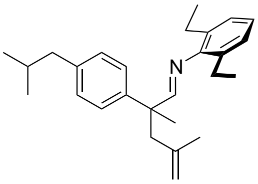

Imine was synthesized according to “General procedure for synthesis of imines”. **5** (1.50 g, 6.10 mmol), **6b** (916 mg, 6.10 mmol), and PTSA (11.0 mg, 61.0 µmol) were used to afford product **7b** as a yellow oily liquid (2.10 g, 5.60 mmol, 91%).

3.1.3 2-(4-isobutylphenyl)-*N*-(2-isopropyl-6-methylphenyl)-2,4-dimethylpent-4-en-1-imine (**7a**)

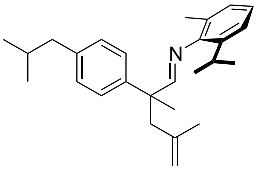

Imine was synthesized according to “General procedure for synthesis of imines”. **5** (3.40 g, 8.40 mmol), **6c** (1.25 g, 8.40 mmol), and PTSA (14.0 mg, 84.0 µmol) were used to afford product **7c** as a yellow oily liquid (2.03 g, 5.40 mmol, 65%).

3.1.4 *N*-(2,5-di-*tert*-butylphenyl)-2-(4-isobutylphenyl)-2,4-dimethylpent-4-en-1-imine (**7e**)

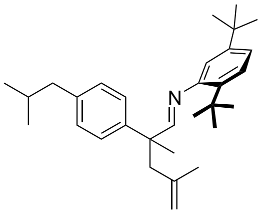

Imine was synthesized according to "General procedure for synthesis of imines". **5** (1.40 g, 5.70 mmol), **6d** (1.19 g, 5.70 mmol), and PTSA (10.0 mg, 57.0 µmol) were used to afford product **7d** as a yellow oil (2.25 g, 5.20 mmol, 91%).

3.1.5 *N*-(2,6-diisopropylphenyl)-2-(4-isobutylphenyl)-2,4-dimethylpent-4-en-1-imine (**7d**)

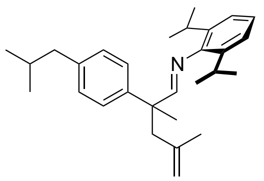

Imine was synthesized according to "General procedure for synthesis of imines". **5** (1.40 g, 5.70 mmol), **6e** (1.02 g, 5.70 mmol), and PTSA (10.0 mg, 57.0 µmol) were used to afford product **7e** as a yellow oily liquid (2.10 g, 5.20 mmol, 91%).

4 Synthesis of tetrafluoroborate CAACs precursors

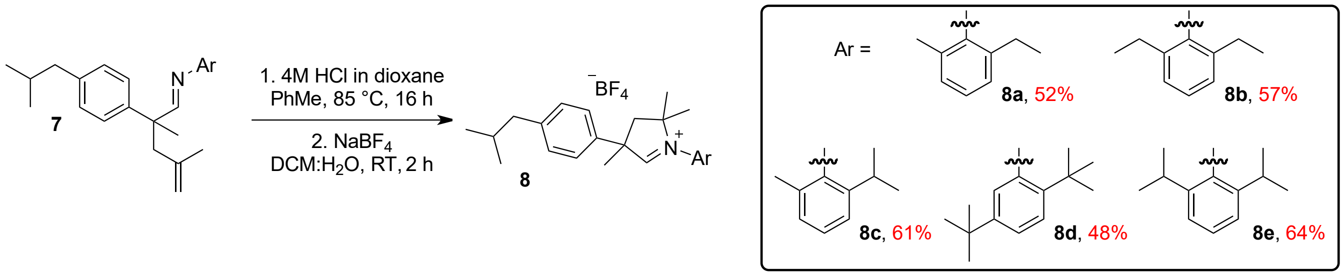

4.1 General procedure for salts synthesis

Under protective argon atmosphere in a round bottom flask equipped with magnetic stirring bar imine (**7a**–**e**, 1.00 equiv.) was dissolved in anh. toluene (c = 0.5 M), followed by addition of hydrogen chloride (c = 4.0 M in dioxane, 2.50 equiv.) at 0 °C. The reaction mixture was stirred at 85 °C for 16 hours, cooled to RT, and the solvent was evaporated under reduced pressure. The crude product was dissolved in a small amount of DCM, $NaBF_4$ (2.00 equiv.) dissolved in H_2O (ca. 10 mL) was added, and the mixture was stirred at RT for 2 hours. The reaction mixture was transferred to separation funnel and extracted with DCM three times, organic layers were combined, dried over anh. $MgSO_4$, and filtrated on Schott funnel through neutral celite. The solvent was evaporated under reduced pressure and the crude product was dissolved in small amount of MeOH followed by addition of Et_2O. Precipitate was formed, filtered, and dried *in vacuo* to afford the final products **8a**–**e**.

4.1.1 1-(2-ethyl-6-methylphenyl)-4-(4-isobutylphenyl)-2,2,4-trimethyl-3,4-dihydro-2*H*-pyrrol-1-ium tetrafluoroborate (8a)

Salt **8a** was synthesized according to "General procedure for salts synthesis". **7a** (1.85 g, 5.10 mmol), hydrogen chloride (3.20 mL, 4.0 M in dioxane, 12.8 mmol), and sodium tetrafluoroborate (1.12 g, 10.2 mmol) were used to afford product as a colourless solid (1.22 g, 2.70 mmol, 52%).

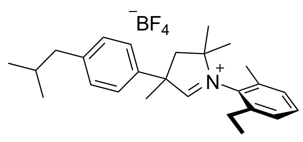

^{1}H NMR (400 MHz, $CDCl_3$): δ 9.51 – 9.41 (m, 1H), 7.44 – 7.32 (m, 3H), 7.35 – 7.27 (m, 0.5×H), 7.27 – 7.18 (m, 3H), 7.21 – 7.13 (m, 0.5×1H), 3.15 (ddd, *J* = 14.0, 9.3, 1.9 Hz, 1H), 2.66 (dd, *J* = 14.0, 2.3 Hz, 1H), 2.63 – 2.52 (m, 1H), 2.50 – 2.42 (m, 2H), 2.41 – 2.28 (m, 2H), 2.24 – 2.03 (m, 2H), 1.99 – 1.91 (m, 3H), 1.91 – 1.78 (m, 1H), 1.58 (d, *J* = 15.1 Hz, 3H), 1.38 (s, 3H), 1.33 – 1.23 (m, 0.5×3H), 1.11 – 1.01 (m, 0.5×3H), 0.92 – 0.85 (m, 6H); **^{13}C NMR (101 MHz, $CDCl_3$):** δ 189.9, 142.4, 140.2, 139.6, 137.5, 137.4, 134.3, 133.2, 131.8, 131.7, 131.2, 130.8, 130.7, 130.2, 130.0, 127.9, 127.9, 125.6, 125.6, 83.9, 55.4, 55.3, 48.5, 48.4, 45.0, 45.0, 30.2, 30.2, 29.3, 28.9, 28.0, 27.5, 27.3, 26.9, 25.0, 24.8, 22.5, 22.4, 22.4, 22.4, 19.5, 19.2, 15.6, 14.8; **HRMS-ESI (*m*/*z*)**: calculated for $C_{26}H_{36}N^+$ ([M]$^+$): 362.2842, found: 362.2842; **Elemental analysis**: calculated for $C_{26}H_{36}BF_4N$: C, 69.49; H, 8.08; N, 3.12; found: C, 69.59; H, 7.97; N, 3.24; **IR**: 3061, 2957, 2933, 2870, 1643, 1513, 1465, 1384, 1349, 1269, 1051, 1035, 797, 732, 701, 645, 573, 521.

4.1.2 1-(2,6-diethylphenyl)-4-(4-isobutylphenyl)-2,2,4-trimethyl-3,4-dihydro-2*H*-pyrrol-1-ium tetrafluoroborate (**8b**)

Salt **8b** was synthesized according to "General procedure for salts synthesis". **7b** (1.85 g, 4.90 mmol), hydrogen chloride (3.08 mL, 4.0 M in dioxane, 12.3 mmol), and sodium tetrafluoroborate (1.08 g, 9.90 mmol) were used to afford product as a colourless solid (1.30 g, 2.80 mmol, 57%).

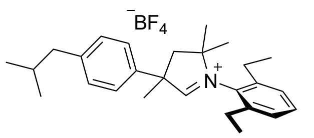

^{1}H NMR (400 MHz, $CDCl_3$): δ 9.49 (s, 1H), 7.49 – 7.35 (m, 3H), 7.34 – 7.27 (m, 1H), 7.26 – 7.17 (m, 3H), 3.18 (d, *J* = 14.1 Hz, 1H), 2.64 (d, *J* = 14.0 Hz, 1H), 2.57 (q, *J* = 7.6 Hz, 2H), 2.49 – 2.44 (m, 2H), 2.32 (dq, *J* = 15.1, 7.6 Hz, 1H), 2.13 (dq, *J* = 14.6, 7.3 Hz, 1H), 1.93 (s, 3H), 1.91 – 1.78 (m, 1H), 1.54 (s, 3H), 1.33 (s, 3H), 1.28 (t, *J* = 7.5 Hz, 3H) 1.07 (t, *J* = 7.5 Hz, 3H), 0.88 (dd, *J* = 6.6, 2.0 Hz, 6H); **^{13}C NMR (101 MHz, $CDCl_3$):** δ 189.8, 142.4, 140.0, 139.5, 137.5, 131.4, 130.8, 130.7, 127.9, 127.8, 125.6, 83.7, 55.3, 48.2, 45.0, 30.2, 29.1, 27.5, 26.8, 25.1, 24.8, 22.4, 22.4, 15.6, 14.7; **HRMS-ESI (*m*/*z*)**: calculated for $C_{27}H_{38}N^+$ ($[M]^+$): 376.2999, found: 376.2998; **Elemental analysis**: calculated for $C_{27}H_{38}BF_4N$: C, 69.98; H, 8.27; N, 3.02; found: C, 69.85; H, 8.25; N, 3.19; **IR**: 3053, 2957, 2935, 2871, 1643, 1513, 1464, 1383, 1349, 1271, 1051, 1034, 798, 733, 701, 572, 520.

4.1.3 4-(4-isobutylphenyl)-1-(2-isopropyl-6-methylphenyl)-2,2,4-trimethyl-3,4-dihydro-2*H*-pyrrol-1-ium tetrafluoroborate (**8c**)

Salt **8c** was synthesized according to "General procedure for salts synthesis". **7c** (2.00 g, 5.30 mmol), hydrogen chloride (3.33 mL, 4.0 M in dioxane, 13.3 mmol), and sodium tetrafluoroborate (1.17 g, 10.6 mmol) were used to afford product as a colourless solid (1.51 g, 3.30 mmol, 61%).

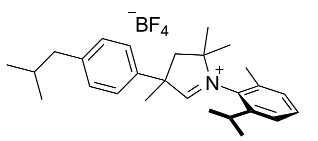

^{1}H NMR (400 MHz, $CDCl_3$): δ 9.52 (s, 0.67×1H), 9.47 (s, 0.33×1H), 7.47 – 7.30 (m, 4H), 7.25 – 7.15 (m, 3H), 3.26 – 3.11 (m, 1H), 2.76 – 2.62 (m, 1H+0.67×1H), 2.52 – 2.42 (m, 2H), 2.40 – 2.24 (m, 1H), 2.07 (s, 2H), 1.96 (s, 1H), 1.93 – 1.78 (m, 3H), 1.61 (s, 1H), 1.56 (s, 2H), 1.42 – 1.32 (m, 5H), 1.23 – 1.12 (m, 3H), 0.97 (d, *J* = 6.7 Hz, 1H), 0.92 – 0.85 (m, 6H); **^{13}C NMR (101 MHz, $CDCl_3$):** δ 189.9, 189.8, 145.0, 144.4, 142.5, 142.3, 137.5, 137.4, 134.3, 133.3, 131.6, 131.5, 130.8, 130.8, 130.7, 130.7, 130.2, 130.2, 125.6, 125.5, 125.3, 125.1, 84.0, 83.5, 55.4, 55.3, 48.4, 48.1, 45.0, 44.9, 30.2, 30.2, 30.0, 29.3, 29.2, 28.8, 28.1, 27.5, 27.1, 26.6, 26.1, 25.9, 22.5, 22.4, 22.4, 22.4, 22.1, 22.0, 19.7, 19.2; **HRMS-ESI (*m*/*z*)**: calculated for $C_{27}H_{38}N^+$ ($[M]^+$): 376.3004, found: 376.3001; **Elemental analysis**: calculated for $C_{27}H_{38}BF_4N$: C, 69.98; H, 8.27; N, 3.02; found: C, 69.95; H, 8.25; N, 3.21; **IR:** 3055, 2961, 2934, 2872, 1643, 1464, 1035, 953, 796, 762, 520.

4.1.4 1-(2,5-di-*tert*-butylphenyl)-4-(4-isobutylphenyl)-2,2,4-trimethyl-3,4-dihydro-2*H*-pyrrol-1-ium tetrafluoroborate (**8d**)

Salt **8d** was synthesized according to "General procedure for salts synthesis". **7d** (2.25 g, 5.20 mmol), hydrogen chloride (3.26 mL, 4.0 M in dioxane, 13.0 mmol), and sodium tetrafluoroborate (1.14 g, 10.4 mmol) were used to afford product as a colourless solid (1.30 g, 2.50 mmol, 48%).

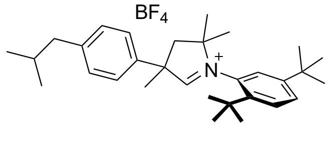

^{1}H NMR (400 MHz, $CDCl_3$): δ 9.75 – 9.66 (m, 0.53×1H), 9.29 – 9.23 (m, 0.47×1H), 7.67 – 7.61 (m, 0.53×1H), 7.55 – 7.46 (m, 1H + 0.47×1H), 7.40 – 7.33 (m, 2H), 7.32 – 7.25 (m, 0.47×1H), 7.24 – 7.16 (m, 2H), 6.65 (bs, 0.53×1H), 3.26 – 3.15 (m, 0.53×1H), 3.07 (d, *J* =

13.6 Hz, 0.47×1H), 2.76 – 2.67 (m, 0.47×1H), 2.53 (d, *J* = 13.9 Hz, 0.53×1H), 2.48 – 2.43 (m, 2H), 2.04 – 1.98 (m, 0.47×3H), 1.90 – 1.80 (m, 4H), 1.50 (d, *J* = 4.2 Hz, 3H), 1.46 – 1.41 (m, 0.53×9H), 1.36 (s, 0.53×3H), 1.35 – 1.32 (m, 0.47×9H), 1.27 – 1.24 (m, 0.53×9H), 1.11 – 1.08 (m, 0.47×9H), 0.91 – 0.85 (m, 6H); **^{13}C NMR (101 MHz, $CDCl_3$):** δ 188.6, 187.2, 150.9, 150.4, 142.2, 142.0, 141.9, 141.2, 137.9, 137.0, 132.1, 131.0, 131.0, 130.8, 130.5, 128.8, 128.5, 125.6, 125.5, 125.2, 125.1, 123.1, 82.4, 81.5, 55.0, 54.8, 48.6, 46.9, 45.0, 44.9, 37.6, 37.0, 34.6, 34.5, 33.7, 33.6, 30.9, 30.9, 30.4, 30.2, 30.1, 29.9, 28.4, 28.3, 27.1, 26.1, 22.5, 22.5, 22.4; **HRMS-ESI (*m*/*z*)**: calculated for $C_{31}H_{46}N^+$ ($[M]^+$): 432.3625, found: 432.3626; **Elemental analysis**: calculated for $C_{31}H_{46}BF_4N$: C, 71.67; H, 8.93; N, 2.70; found: C, 71.44; H, 8.87; N, 2.76; **IR**: 3058, 2959, 2870, 1646, 1613, 1503, 1467, 1395, 1366, 1269, 1054, 848, 799, 734, 561, 520.

4.1.5 1-(2,6-diisopropylphenyl)-4-(4-isobutylphenyl)-2,2,4-trimethyl-3,4-dihydro-2*H*-pyrrol-1-ium tetrafluoroborate (**8e**)

Salt **8e** was synthesized according to "General procedure for salts synthesis". **7e** (2.05 g, 5.10 mmol), hydrogen chloride (3.17 mL, 4.0 M in dioxane, 12.7 mmol), and sodium tetrafluoroborate (1.12 g, 10.2 mmol) were used to afford product as a colourless solid (1.60 g, 3.30 mmol, 64%).

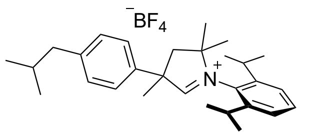

^{1}H NMR (400 MHz, $CDCl_3$): δ 9.58 – 9.51 (m, 1H), 7.54 – 7.38 (m, 3H), 7.36 – 7.30 (m, 1H), 7.28 – 7.19 (m, 3H), 3.26 (dd, *J* = 14.1, 1.7 Hz, 1H), 2.76 – 2.60 (m, 2H), 2.53 – 2.40 (m, 2H), 2.24 (sept, *J* = 6.8 Hz, 1H), 1.94 – 1.77 (m, 4H), 1.55 (bs, *J* = 1.6 Hz, 3H), 1.38 – 1.34 (m, 3H), 1.31 (s, 3H), 1.23 – 1.17 (m, 3H), 1.13 – 1.09 (m, 3H), 1.06 – 1.01 (m, 3H), 0.91 – 0.86 (m, 6H); **^{13}C NMR (101 MHz, $CDCl_3$):** δ 189.9, 144.9, 144.4, 142.4, 137.3, 132.1, 130.7, 128.9, 125.7, 125.5, 83.5, 55.4, 47.7, 45.0, 30.3, 30.2, 29.2, 29.0, 27.5, 26.3, 26.2, 26.0, 22.4, 22.4, 22.3, 22.3.; **HRMS-ESI (*m*/*z*)**: calculated for $C_{29}H_{42}N^+$ ($[M]^+$): 404.3312, found: 202.3314; **Elemental analysis**: calculated for $C_{29}H_{42}BF_4N$: C, 70.87; H, 8.61; N, 2.85; found: C, 70.68; H, 8.40; N, 2.85; **IR**: 3055, 2958, 2933, 2868, 1638, 1509, 1471, 1351, 1269, 1106, 1054, 1039, 880, 808, 735, 704, 581, 566, 520.

5 Synthesis of ruthenium complexes

5.1 General procedure for ruthenium complexes synthesis

In a dry Schlenk flask equipped with magnetic stirring bar, salt (**8a–e**, 2.20 equiv.) and Hoveyda-Grubbs 1st generation complex (**Hov I**) (1.00 equiv.) were suspended in THF (c_{CAAC} = 0.10 M), and the mixture was stirred for 1 minute. Next, LiHMDS was added (2.20 equiv.) and the resulting mixture was stirred until full consumption of substrates. Then, the crude mixture was filtered through a short pad of neutral aluminum oxide (Al_2O_3, neutral, Broockman Grade I) with Et_2O or DCM as an eluent. Green fraction was collected and the solvent was evaporated under reduced pressure. Next small amount of *n*-pentane was added to the residue and the mixture was placed in a sonic bath. The precipitate was filtered and washed with cold *n*-pentane (**Ru13**, **Ru15**) or *n*-heptane and next with diethyl ether (**Ru14**). Column chromatography (SiO_2, 10% AcOEt in *n*-hexane) to purify crude product was done for **Ru11** and **Ru12** and next products were precipitated from *n*-pentane.

5.1.1 **Ru11**

Complex **Ru11** was synthesized according to "General procedure for ruthenium complexes synthesis". **Hov I** (120 mg, 200 μmol), **8a** (198 mg, 440 μmol), and LiHMDS (73.5 mg, 440 μmol) were used to afford product as a green powder (102 mg, 150 μmol, 74%).

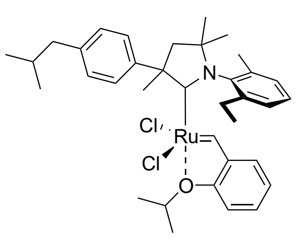

^{1}H NMR (400 MHz, CD_2Cl_2): δ 17.65 (s, 0.31×1H), 16.31 (s, 0.69×1H); **^{13}C NMR (101 MHz, CD_2Cl_2):** δ 300.8 (only diagnostic benzylidene proton and carbon signals are reported due to the presence of conformational and rotational isomers resulting in high complexity of spectra, which precluded further assignment); **HRMS-ESI (*m/z*)**: calculated for $C_{36}H_{47}NOCl_2Ru^+$ ([M]$^+$): 681.2073, found: 681.2071; **Elemental analysis**: calculated for $C_{36}H_{47}NOCl_2Ru$: C, 63.42; H, 6.95; N, 2.05; found: C, 63.27; H, 6.95; N, 2.13; **IR**: 2981, 2945, 2869, 1588, 1576, 1516, 1476, 1456, 1374, 1315, 1298, 1242, 1226, 1160, 1140, 1117, 1096, 1040, 997, 932, 881, 844, 807, 778, 755, 690, 569, 544.

5.1.2 **Ru12**

Complex **Ru12** was synthesized according to "General procedure for ruthenium complexes synthesis". **Hov I** (150 mg, 150 μmol), **8b** (255 mg, 549 μmol), and LiHMDS (91.9 mg, 549 μmol) were used to afford product as a green powder (125 mg, 180 μmol, 71%).

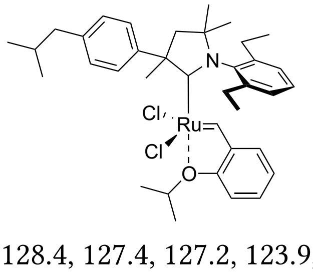

^{1}H NMR (400 MHz, CD_2Cl_2): δ 17.65 (bs, 0.15×1H), 16.39 (s, 0.85×1H), 8.37 – 7.87 (m, 2H), 7.70 – 7.25 (m, 6H), 6.96 – 6.68 (m, 3H), 4.96 (sept, *J* = 6.1 Hz, 1H), 3.12 (d, *J* = 12.9 Hz, 1H), 2.79 – 2.62 (m, 2H), 2.62 – 2.42 (m, 4H), 2.40 – 2.26 (m, 3H), 1.98 – 1.85 (m, 1H), 1.58 – 1.45 (m, 6H), 1.41 – 1.25 (m, 9H), 1.07 – 1.00 (m, 2H), 0.98 – 0.90 (m, 6H), 0.81 – 0.73 (m, 2H); **^{13}C NMR (101 MHz, CD_2Cl_2):** δ 299.9, 263.5, 152.5, 144.3, 143.9, 143.6, 141.4, 141.3, 139.1, 131.2, 130.4, 129.4, 128.4, 127.4, 127.2, 123.9, 122.2, 113.5, 78.3, 75.0, 63.3, 54.4, 54.1, 53.8, 53.6, 53.3, 48.4, 45.3, 31.2, 30.7, 30.1, 28.2, 27.7, 25.8, 24.5, 22.8, 22.6, 22.4, 14.9, 14.4; **HRMS-ESI (*m/z*)**: calculated for $C_{37}H_{49}NOCl_2Ru$ ([M]$^+$): 695.2229, found: 695.2228; **Elemental analysis**: calculated for $C_{37}H_{49}NOCl_2Ru$: C, 63.42; H, 6.95; N, 2.05; found: C, 63.27; H, 6.95; N, 2.13; **IR**: 2948, 2932, 2871, 1588, 1576, 1516, 1475, 1455, 1442, 1412, 1387, 1374, 1298, 1225, 1159, 1141, 1116, 1096, 1040, 997, 932, 881, 843, 807, 792, 773, 754, 693, 602, 568, 544.

5.1.3 Ru13

Complex **Ru13** was synthesized according to "General procedure for ruthenium complexes synthesis". **Hov I** (150 mg, 250 µmol), **8c** (255 mg, 549 µmol), and LiHMDS (91.9 mg, 549 µmol) were used to afford product as a green powder (75 mg, 108 µmol, 43%).

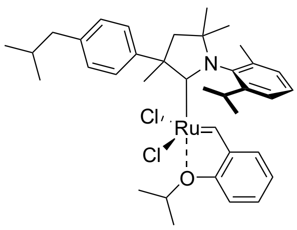

^{1}H NMR (400 MHz, $CDCl_3$): δ 16.37 (bs, 0.28×1H), 16.35 (s, 0.72×1H), 8.24 – 8.07 (m, 2H), 7.59 – 7.43 (m, 3H), 7.37 – 7.22 (m, 3H), 6.89 – 6.70 (m, 3H), 4.95 (dsept, *J* = 12.3, 6.2 Hz, 1H), 3.24 – 2.89 (m, 2H), 2.63 – 2.45 (m, 2H), 2.45 – 2.31 (m, 3H), 2.29 (s, 2H), 2.24 (s, 2H), 1.99 – 1.86 (m, 1H), 1.63 – 1.52 (m, 6H), 1.51 – 1.41 (m, 4H), 1.38 – 1.31 (m, 4H), 1.25 (d, *J* = 6.6 Hz, 1H), 1.00 – 0.91 (m, 6H), 0.84 (d, *J* = 6.5 Hz, 2H), 0.51 (d, *J* = 6.3 Hz, 1H); **^{13}C NMR (101 MHz, $CDCl_3$):** δ 301.0, 300.5, 264.7, 264.2, 152.6, 152.5, 148.9, 148.9, 144.1, 143.8, 141.1, 141.0, 139.8, 138.6, 138.5, 138.4, 138.3, 131.1, 131.0, 130.4, 130.2, 129.8, 129.8, 129.2, 129.1, 128.8, 128.0, 126.2, 125.6, 124.3, 124.1, 121.9, 121.8, 113.4, 113.3, 78.1, 77.9, 74.6, 63.3, 62.7, 49.2, 47.7, 45.2, 32.5, 31.2, 30.5, 30.4, 28.8, 28.3, 28.2, 27.6, 27.4, 26.9, 25.9, 24.2, 24.1, 22.9, 22.8, 22.7, 22.6, 22.5, 22.5, 22.5, 22.2, 22.0, 21.1; **HRMS-ESI (*m*/*z*)**: calculated for $C_{37}H_{49}NOCl_2Ru^+$ ([M]$^+$): 695.2235, found: 695.2227; **Elemental analysis**: calculated for $C_{37}H_{49}NOCl_2Ru$: C, 63.87; H, 7.10; N, 2.01; found: C, 64.00; H, 7.17; N, 2.07; **IR:** . 3055, 2972, 2951, 2927, 2867, 1589, 1577, 1515, 1477, 1453, 1406, 1384, 1375, 1299, 1226, 1157, 1140, 1113, 1097, 1039, 997, 935, 843, 799, 787, 741, 694, 607, 569, 546.

5.1.4 Ru14

Complex **Ru14** was synthesized according to "General procedure for ruthenium complexes synthesis". **Hov I** (300 mg, 499 µmol), **8d** (571 mg, 1.10 mmol), and LiHMDS (184 mg, 1.10 mmol) were used to afford product as a green powder (328 mg, 436 µmol, 87%).

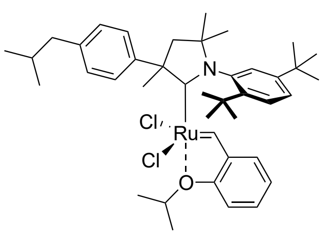

^{1}H NMR (400 MHz, CD_2Cl_2): δ 17.84 (s, 0.81×1H), 16.79 (s, 0.19×1H), 8.27 (d, *J* = 2.3 Hz, 0.81×1H), 8.13 (d, *J* = 8.1 Hz, 0.19×1H), 7.76 – 7.42 (m, 5H), 7.30 (d, *J* = 8.0 Hz, 0.19×2H), 7.20 (d, *J* = 2.3 Hz, 0.19×1H), 7.11 (d, *J* = 8.0 Hz, 0.81×2H), 7.08 (dd, *J* = 1.6, 7.6 Hz, 0.81×1H), 6.91 (d, *J* = 8.4 Hz, 0.81×1H), 6.86 (td, *J* = 0.8, 7.4 Hz, 0.81×1H), 6.78 (t, *J* = 7.4 Hz, 0.19×1H), 6.69 (dd, *J* = 1.7, 7.6 Hz, 0.19×H), 5.03 (sept, *J* = 5.7 0.81×Hz, 1H), 4.93 (sept, *J* = 5.2 Hz, 0.19×H), 3.08 (d, *J* = 13.6 Hz, 0.19×1H), 2.61 – 0.84 (m, 0.81×1H + 43H); **^{13}C NMR (101 MHz, CD_2Cl_2):** δ 296.3, 296.0, 271.8, 262.5, 153.5, 152.6, 149.9, 148.8, 148.1, 145.2, 144.4, 144.1, 143.6, 143.6, 140.9, 140.1, 139.8, 139.0, 137.0, 131.8, 131.1, 131.0, 130.8, 130.8, 130.5, 130.1, 129.3, 129.2, 126.6, 126.3, 125.3, 123.4, 123.3, 121.9, 121.8, 113.4, 113.2, 76.4, 74.8, 74.7, 74.4, 64.0, 63.1, 56.0, 50.1, 45.2, 45.1, 37.5, 37.1, 34.9, 34.8, 34.2, 34.0, 33.9, 33.2, 31.6, 31.1, 30.3, 30.3, 28.6, 28.5, 28.2, 27.8, 22.9, 22.6, 22.6, 22.6, 22.5, 22.2, 21.9, 21.9; **HRMS-ESI (*m*/*z*)**: calculated for $C_{41}H_{57}NOCl_2Ru^+$ ([M]$^+$): 751.2855, found: 751.2857; **Elemental analysis**: calculated for $C_{41}H_{57}NOCl_2Ru$: C, 65.50; H, 7.64; N, 1.86; found: C, 65.51; H, 7.83; N, 1.99; **IR**: 3069, 2995, 2955, 2866, 1589, 1575, 1499, 1475, 1454, 1397, 1387, 1370, 1362, 1314, 1300, 1271, 1249, 1221, 1150, 1141, 1116, 1099, 1087, 1070, 1024, 1017, 941, 882, 844, 831, 819, 793, 775, 745, 707, 687, 613, 583, 567, 549, 442.

5.1.5 **Ru15**

Complex **Ru15** was synthesized according to "General procedure for ruthenium complexes synthesis". **Hov I** (170 mg, 250 μmol), **8e** (270 mg, 549 μmol), and LiHMDS (91.9 mg, 549 μmol) were used to afford product as a green powder (131 mg, 181 μmol, 72%).

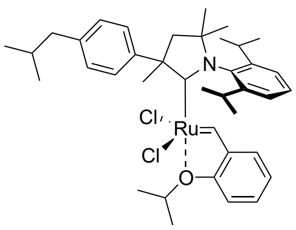

^{1}H NMR (400 MHz, CD_2Cl_2): δ 16.50 (s, 1H), 8.14 (d, *J* = 8.3 Hz, 2H), 7.65 (t, *J* = 7.8 Hz, 1H), 7.55 – 7.47 (m, 2H), 7.46 (dd, *J* = 7.7, 1.6 Hz, 1H), 7.30 (d, *J* = 8.4 Hz, 2H), 6.90 (d, *J* = 8.4 Hz, 1H), 6.83 (td, *J* = 7.4, 0.8 Hz, 1H), 6.72 (dd, *J* = 7.6, 1.7 Hz, 1H), 4.95 (sept, *J* = 6.2 Hz, 1H), 3.13 (d, *J* = 12.8 Hz, 1H), 3.05 (sept, *J* = 6.6 Hz, 1H), 2.94 (sept, *J* = 6.4 Hz, 1H), 2.58 (dd, *J* = 13.2, 6.9 Hz, 1H), 2.49 (dd, *J* = 13.1, 7.6 Hz, 1H), 2.32 (d, *J* = 12.8 Hz, 1H), 2.30 (s, 3H), 2.01 – 1.83 (m, 1H), 1.56 (d, *J* = 6.2 Hz, 3H), 1.50 (s, 3H), 1.43 – 1.35 (m, 6H), 1.34 (d, *J* = 6.6 Hz, 3H), 1.25 (d, *J* = 6.6 Hz, 3H), 0.96 (d, *J* = 6.7 Hz, 3H), 0.93 (d, *J* = 6.6 Hz, 3H), 0.78 (d, *J* = 6.5 Hz, 3H), 0.47 (d, *J* = 6.4 Hz, 3H); **^{13}C NMR (101 MHz, CD_2Cl_2):** δ 296.7, 264.7, 153.0, 148.7, 148.5, 143.2, 141.2, 141.1, 137.3, 131.1, 130.3, 129.9, 128.9, 126.3, 126.1, 124.1, 122.1, 113.6, 77.9, 75.0, 63.2, 48.3, 45.3, 32.9, 30.7, 29.1, 28.8, 28.6, 28.1, 27.4, 26.5, 24.6, 24.4, 22.8, 22.6, 22.5, 22.4; **HRMS-ESI (*m/z*)**: calculated for $C_{39}H_{53}NOCl_2Ru^+$ ($[M]^+$): 723.2542, found: 723.2537; **Elemental analysis**: calculated for $C_{39}H_{53}Cl_2NORu$: C, 64.72; H, 7.38; N, 1.94; found: C, 64.34; H, 7.43; N, 1.79; **IR**: 2978, 2953, 2923, 2868, 1586, 1575, 1516, 1473, 1454, 1402, 1374, 1313, 1293, 1224, 1158, 1142, 1114, 1097, 1038, 997, 936, 844, 809, 754, 569, 548.

6 Solubility of complexes in *n*-hexane

A few mg of complexes (**Ru1**, **Ru11–15**) was placed in a round bottom flask equipped with stirring bar. n-Hexane (HPLC grade) was added in 1 mL portions until full solubility of complex was observed.

Table S1 Solubility of complexes **Ru1**, **Ru11–Ru15** in *n*-hexane

Complex	**Ru1**	**Ru11**	**Ru12**	**Ru13**	**Ru14**	**Ru15**
Solubility [mg mL^{-1}]	0.039	0.400	0.580	0.478	1.525	0.333

7 Ethenolysis of methyl oleate (9)

7.1 Preparation of **9**

Oleic acid (500 g) was dissolved in acetone (99.9% in ratio 1 g of acid in 11 mL of solvent).[4] The mixture was cooled to −40 °C and stirred for 16 hours. Solid was filtered through Büchner funnel, transferred to a round bottom flask and remaining acetone was evaporated on rotary evaporator and under high vacuum. Process was repeated 2–4 times. Remaining oleic acid was dissolved in MeOH and stirred under argon for 16 hours with catalytic amount of *p*-toluenesulfonic acid. After cooling to RT, $MgSO_4$ was added and the reaction mixture was stirred for 2 hours, solid was filtered, and MeOH was evaporated under reduced pressure. Freshly prepared methyl oleate was placed in a round bottom flask equipped with magnetic stirring bar. Activated alumina (2.5 wt%) was added and distillation setup was assembled. Flask content was heated under high vacuum at 60 °C for 1 hour before distillation started. 10% of heads and 10% of tails were discarded. Freshly distilled methyl oleate was treated with 2.5 wt% of activated alumina and 0.1 mol% of BHT (2,6-di-*tert*-butyl-4-methylphenol), gases were evacuated, and the content of the flask was stirred at 100 °C for ca. 1 hour under gentle flow of argon. After cooling to RT, methyl oleate was filtered through pad of activated alumina under argon to flask and stored over activated alumina in the dark.

7.2 General procedure for ethenolysis of **9**

Under protective argon atmosphere complex (**Ru1**, **Ru11**–**15**, ca. 5 mg) was placed in Schlenk flask and dissolved in 5 mL of anh. toluene to prepare stock solution. Methyl oleate was filtered through pad of alumina to Schlenk flask equipped with magnetic stirring bar and degassed under vacuum ($p \sim 1 \times 10^{-2}$). The amount of stock solution containing 3 or 1 ppm of Ru-complex was added to methyl oleate under argon. The mixture was immediately transferred to an autoclave containing glass vessel equipped with a magnetic stirring bar. Next, autoclave was filled with ethylene 3.5 or 4.5 (10 bar) and reaction mixture was stirred at 40 °C for 6 h. After that time, pressure was normalized, autoclave was disassembled, solution of SnatchCat in DCM was added, a sample was taken, and subjected to GC analysis.

7.3 Procedure for GC analysis of results

Before reactions, samples of methyl oleate and ethenolysis products were used to determine response factors (RF) on GC. To GC vials known masses of substrate and products were added, dissolved in toluene and analyzed 4 times each on GC.

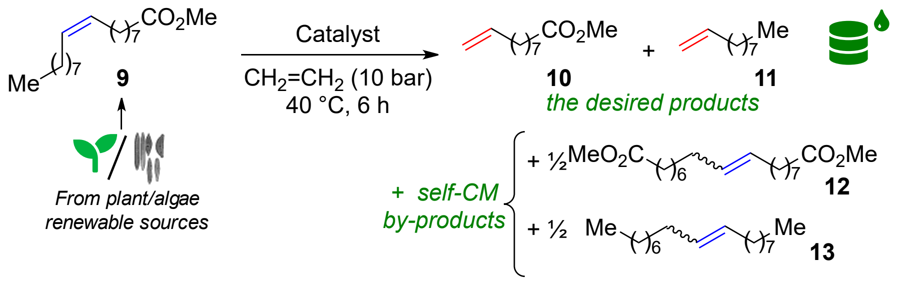

7.4 Results

Table S2 Results for ethenolysis of **9**, in presence of **Ru1**, **Ru11**–**Ru15**

Catalyst	Loading [ppm]	Ethylene grade	Conversion [%]	Selectivity [%]	Yield [%]	TON
**Ru1**	3	3.5	42	96	43	144 000
	1	4.5	28	97	27	274 000
**Ru11**	3	3.5	36	90	32	107 000
	1	4.5	18	89	16	160 000
**Ru12**	3	3.5	54	91	49	164 000
	1	4.5	24	92	22	221 000
**Ru13**	30	3.5	87	87	76	25 000
	20	3.5	79	90	71	35 000
	3	3.5	57	95	54	181 000
	1	4.5	38	96	36	362 000
**Ru14**	3	3.5	68	87	59	197 000
	1	4.5	30	90	27	274 000
**Ru15**	3	3.5	55	97	54	179 000
	1	4.5	27	99	27	270 000

Conversion = $100\times[1 - (A_{\mathbf{9}} \times A^0_{IS})/(A^0_{\mathbf{9}} \times A_{IS})]$; Selectivity = $100\times(n_{\mathbf{10}} + n_{\mathbf{11}})/[(n_{\mathbf{10}} + n_{\mathbf{11}}) + 2\times(n_{\mathbf{12}} + n_{\mathbf{13}})]$; Yield = (Conversion × Selectivity)/100; TON = Yield × $[(n^0_{\mathbf{9}}/n^0_{[Ru]})]/100$; $A_{\mathbf{9}}$, A_{IS} = GC area of methyl oleate and internal standard at the end of the reaction; $A^0_{\mathbf{9}}$, A^0_{IS} = GC area of methyl oleate and internal standard before the reaction;. IS = Internal Standard (methyl stearate).

8 SCM of methyl oleate (9)

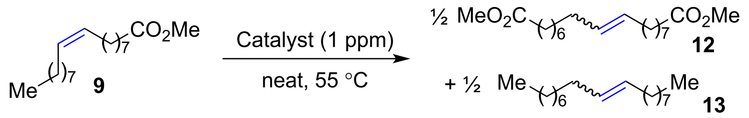

8.1 General procedure for SCM of **9**

Methyl oleate (**9**) was degassed under vacuum (p~1×10^{-2}) and transferred under protective argon atmosphere using syringe glass filter to Schlenk flask (1 equiv.). Substrate (containing internal standard—methyl stearate) was transferred to six vials (1.0 ppm catalyst loading) or Schlenk flasks (2.5 ppm catalyst loading). Under protective argon atmosphere complex (**Ru1**, **Ru11–15**, ca. 5 mg) was placed in separate Schlenk flask and dissolved in 5 mL of anh. toluene. Appropriate amount of stock solution of catalyst was added under argon to the substrate. The mixture was stirred at 55 °C for 4 h (1 ppm) or 24 h (2.5 ppm). During reaction time, samples for GC analyses were taken (ca. 0.1 mL each) to 1.5 mL vials containing ca. 1 mL of toluene and two drops of solution of SnatchCat in DCM.

8.2 Results for 2.5 ppm of catalyst

Table S3 Conversion for SCM of **9** in presence of **Ru1**, **Ru11–Ru15** (2.5 ppm) during 24 h

Time [min]	Catalyst					
	**Ru1**	**Ru11**	**Ru12**	**Ru13**	**Ru14**	**Ru15**
0	0	0	0	0	0	0
5	0	0.1	38.8	0	0	0
15	0.3	21.2	45.7	0	11.5	0
30	3.7	41.5	47.8	0	36.1	0
60	15.0	47.3	49.3	3.9	50.2	0
120	31.9	47.1	49.6	16.0	49.9	0
1440	45.8	49.0	50.5	40.1	51.0	0

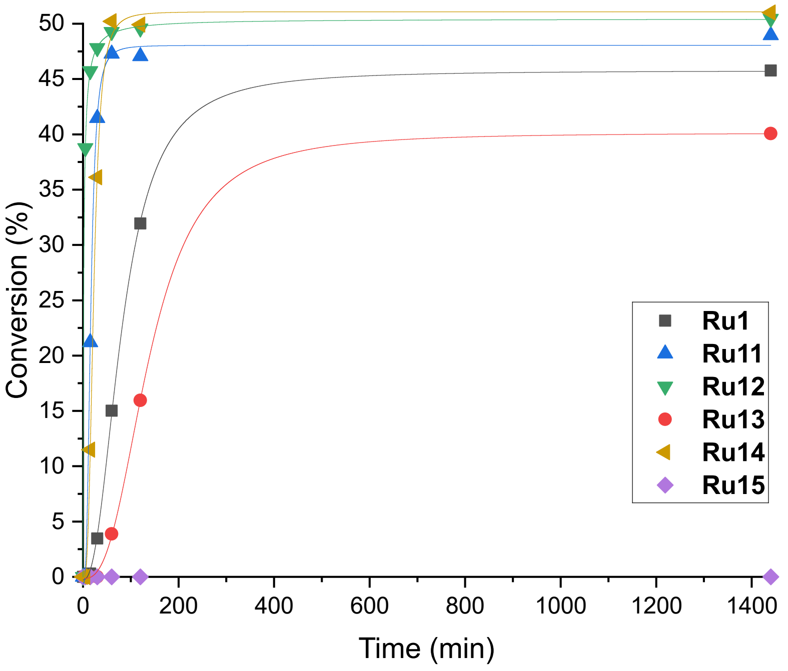

Figure S1 Plot with results for SCM of **9** (2.5 ppm) and trendlines

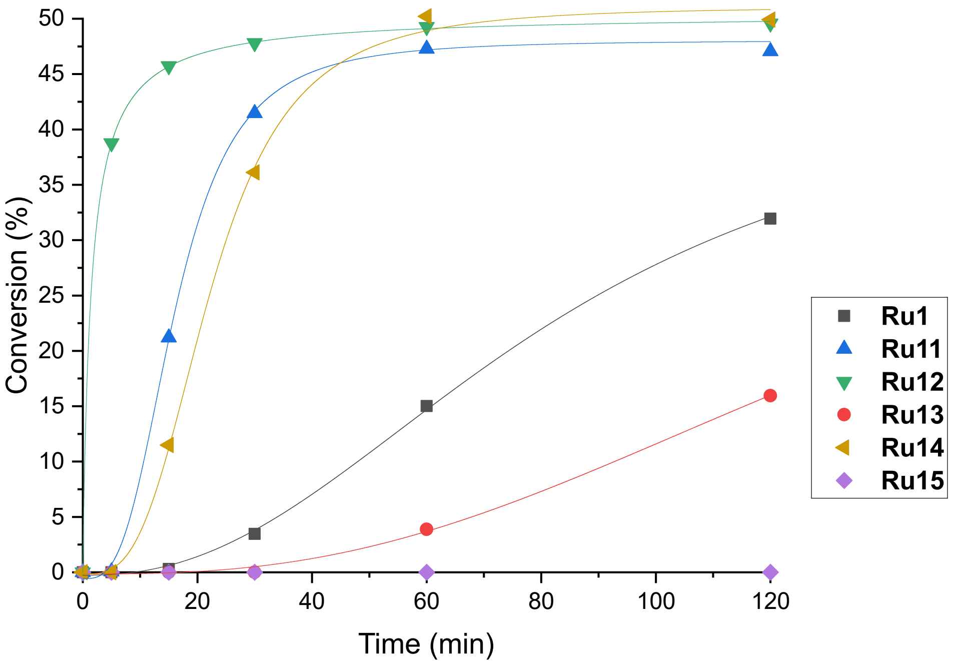

Figure S2 Plot with results for SCM of **9** (2.5 ppm) and trendlines (first 120 min only)

8.3 Results for 1 ppm of catalyst

Table S4 Conversion for SCM of **9** in presence of **Ru1**, **Ru11**–**Ru15** (1 ppm) during 24 h

Time [min]	Catalyst					
	**Ru1**	**Ru11**	**Ru12**	**Ru13**	**Ru14**	**Ru15**
0	0	0	0	0	0	0
5	0.1	3.6	1.3	0	0.7	0
15	0.4	14.6	10.5	0	8.8	0
30	2.6	25.0	25.6	0.6	24.5	0
60	4.2	32.2	38.1	3.9	41.4	0
120	8.4	35.2	42.8	7.5	48.1	0.7
180	10.5	35.6	44.0	10.9	49.5	0.8
240	11.9	35.6	43.7	13.4	49.4	0.2

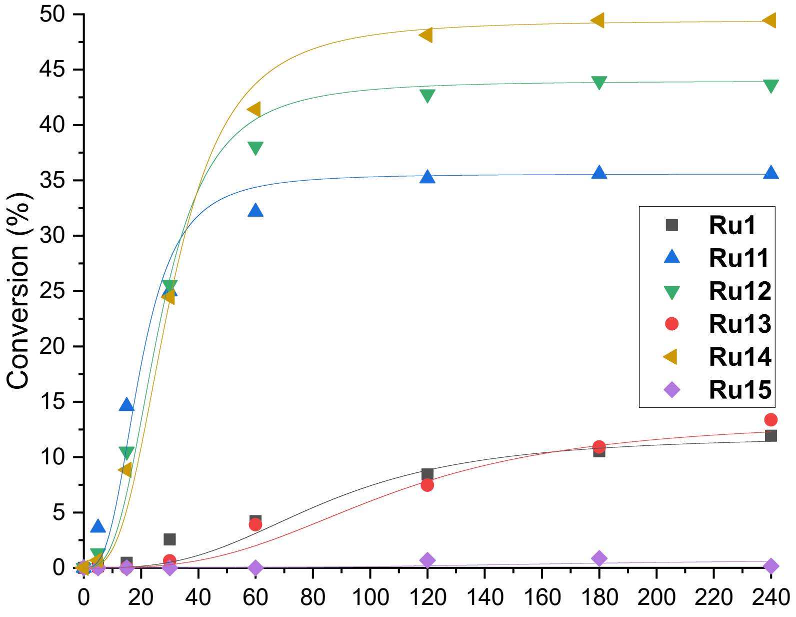

Figure S1 Plot with results for SCM of **9** (1.0 ppm) and trendlines

9 SCM of 1-decene (14)

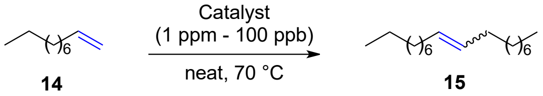

9.1 General procedure for SCM of **14**

Before reaction 1-decene (**14**) was mixed with BHT (a few mg) and neutral activated alumina (2.5 wt%), reflux for 3 hours, distilled under reduced pressure (p~1×10^{-1}) and stored with new batch of alumina in ampule.

14 was transferred under protective argon atmosphere using syringe glass filter to Schlenk flask (1 equiv.). Substrate was mixed with standard (1,3,5-methoxybenzene, 1 equiv.). The mixture was degassed. Under protective argon atmosphere complex (**Ru5** or **Ru12**, ca. 5 mg) was placed in separate Schlenk flask and dissolved in 20 mL of anh. toluene. Appropriate amount of stock solution of catalyst was added under argon to the mixture of substrate and internal standard. The mixture was stirred at 70 °C for 1 or 2 h. After reaction time, samples for GC analyses were taken (ca. 0.2 mL each) to 1.5 mL vials containing ca. 1 mL of toluene and two drops of solution of SnatchCat in DCM.

Yield was calculated using GC measurements and calibration curve. Turnover number was calculated as it was described before.[5] TON = 0.5 × Yield × $[(n^0_{\mathbf{14}} / n^0_{\mathbf{[Ru]}})]/100$. Where: $n^0_{\mathbf{14}}$, $n^0_{\mathbf{[Ru]}}$ = initial moles of 1-decene and catalyst used

9.2 Results

Table S5 Results for SCM of **14** in presence of **Ru5** and **Ru12**

Catalyst	Loading [ppm]	Time [h]	Yield	TON
**Ru5**	1 (4×0.25)	1	74	370 000
**Ru12**	1 (4×0.25)	1	78	390 000
	0.2 (4×0.025)	2	50	1 250 000
	0.1 (4×0.025)	1	31	1 550 000

10 CM of allyl benzene (16) and (*Z*)-but-2-ene-1,4-diyl diacetate (17)

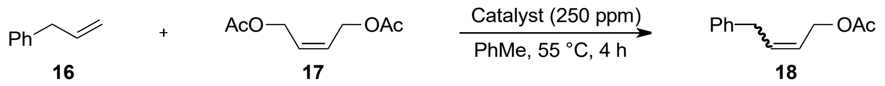

10.1 General procedure for CM of **16** and **17**

Under protective argon atmosphere in Schlenk flask equipped with magnetic stirring bar **16** (69 mg, 0.57 mmol) and **17** (207 mg, 1.14 mmol) were dissolved in anh. PhMe (c = 0.1 M). To this reaction mixture 250 ppm of Ru catalyst (as a stock solution in anh. PhMe) was added and reaction mixture was stirred for 4 hours. For **Ru15** after that time additional 100 ppm of Ru catalyst was added and reaction was stirred additional 12 hours. After completion of reaction solvent was evaporated under reduced pressure and product (**18**) was purified using flash column chromatography (2% EtOAc in *n*-hexane).

10.2 Results

Table S6 Results for CM of **16** and **17** in presence of **Ru1**, **Ru11**–**Ru15** (250 ppm)

Catalyst	Loading [ppm]	Time [h]	Conversion [%]	Selectivity [%]	Yield [%]	*E*:*Z* ratio
**Ru1**	250	4	88	81	72	87:13
**Ru11**	250	4	87	84	74	88:12
**Ru12**	250	4	86	85	74	89:11
**Ru13**	250	4	90	82	74	85:15
**Ru14**	250	4	89	84	75	87:13
**Ru15**	250+**100**	**16**	69	79	55	69:31

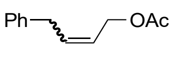

^{1}H NMR (400 MHz, $CDCl_3$): δ 7.34 – 7.28 (m, 2H), 7.25 – 7.15 (m, 3H), 5.93 (dtt, *J* = 15.3, 6.7, 1.3 Hz, 0.87×1H), 5.84 (dtt, *J* = 10.9, 7.5, 1.3 Hz, 0.13×1H), 5.76 – 5.58 (m, 1H), 4.78 – 4.71 (m, 0.13×2H), 4.55 (dq, *J* = 6.4, 1.1 Hz, 0.87×2H), 3.48 (ddd, *J* = 7.5, 1.5, 0.8 Hz, 0.13×2H), 3.44 – 3.38 (m, 0.87×2H), 2.09 (s, 0.13×3H), 2.07 (s, 0.87×3H); **^{13}C NMR (101 MHz, $CDCl_3$)**: δ 171.1, 171.0, 139.9, 139.7, 134.7, 133.6, 128.7, 128.7, 128.6, 128.5, 126.4, 126.3, 125.4, 124.4, 65.0, 60.3, 39.1, 38.8, 33.9, 21.2.

^{1}H and ^{13}C NMR spectra are in agreement with previously reported.[6]

11 CM of undec-10-en-1-yl acetate (20) and acrylonitrile (21)

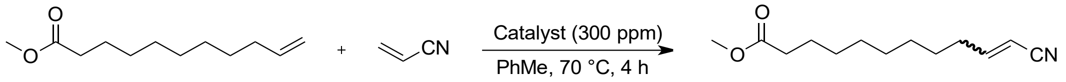

11.1 General procedure for CM of **20** and **21**

Under protective argon atmosphere in Schlenk flask equipped with magnetic stirring bar **20** (50 mg, 0.25 mmol) and acrylonitrile **21** (27 mg, 0.50 mmol) were dissolved in anh. PhMe (c = 0.1 M). To this reaction mixture 300 ppm of Ru precatalyst (as a stock solution in anh. PhMe) was added and reaction mixture was stirred for 3 hours at 70 °C. Yield was estimated by GC.

11.2 Results

Table S7 Results for CM of **20** and **21** in presence of **Ru8**, **Ru11** or **Ru12** (300 ppm)

Catalyst	Loading [ppm]	Yield [%]	*E*:*Z* ratio
**Ru8**	300	97	78:22
**Ru11**	300	96	78:22
**Ru12**	300	96	79:21

12 CM of undec-10-en-1-yl acetate (23) and acrylonitrile (21)

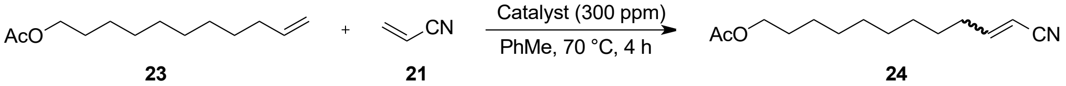

12.1 General procedure for CM of **23** and **21**

Under protective argon atmosphere in Schlenk flask equipped with magnetic stirring bar **23** (175 mg, 0.82 mmol) and acrylonitrile (**21**, 88 mg, 1.65 mmol) were dissolved in anh. PhMe (c = 0.1 M). To this reaction mixture 300 ppm of Ru precatalyst (as a stock solution in anh. PhMe) was added and reaction mixture was stirred for 4 hours. After completion of reaction solvent was evaporated under reduced pressure and product (**25**) was purified using flash column chromatography (2%⟶5% EtOAc in *n*-hexane).

12.2 Results

Table S8 Results for CM of **21** and **23** in presence of **Ru1**, **Ru8**, **Ru11** or **Ru12** (300 ppm)

Catalyst	Loading [ppm]	Yield [%]	*E*:*Z* ratio
**Ru1**	300	51	77:13
**Ru8**	300	61	82:18
**Ru11**	300	72	84:16
**Ru12**	300	83	80:20

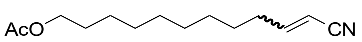

^{1}H NMR (400 MHz, $CDCl_3$): δ 6.71 (dt, *J* = 16.3, 7.0 Hz, 0.21×1H), 6.47 (dt, *J* = 10.9, 7.7 Hz, 0.79×1H), 5.35 – 5.28 (m, 1H), 4.05 (t, *J* = 6.7 Hz, 2H), 2.42 (qd, *J* = 7.6, 1.4 Hz, 0.79×2H), 2.25 – 2.18 (m, 0.21×2H), 2.04 (s, 3H), 1.66 – 1.57 (m, 2H), 1.52 – 1.40 (m, 2H), 1.39 – 1.26 (m, 8H); **^{13}C NMR (101 MHz, $CDCl_3$)**: δ 171.4, 171.4, 156.3, 155.3, 117.7, 116.2, 99.8, 99.7, 77.5, 77.2, 76.8, 64.7, 64.7, 33.4, 32.0, 29.3, 29.3, 29.2, 29.2, 29.0, 29.0, 28.7, 28.3, 27.7, 26.0, 21.2.

13 RCM of diethyl 2,2-diallylmalonate (25)

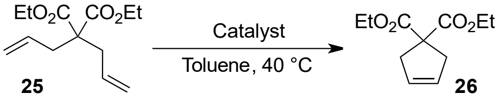

13.1 General procedure for RCM of **25**

Diethyl 2,2-diallylmalonate (**25**) was degassed under vacuum (p~1×10^{-2}) and transferred under protective argon atmosphere using syringe glass filter to Schlenk flask (1 equiv.). Substrate was mixed with standard (1,3,5-methoxybenzene, 1 equiv.). The mixture was degassed and dissolved in anh. toluene (0.1 M). Stock solution of substrate and internal standard was transferred to six Schlenk flasks. Under protective argon atmosphere complex (**Ru1**, **Ru11**–**15**, ca. 5 mg) was placed in separate Schlenk flask and dissolved in 2 mL of anh. toluene. Appropriate amount of stock solution of catalyst (1000 ppm) was added under argon to the mixture of substrate and internal standard. The mixture was stirred at 40 °C for 24 h. During reaction time, samples for GC analyses were taken (ca. 0.3 mL each) to 1.5 mL vials containing ca. 1 mL of toluene and two drops of solution of SnatchCat in DCM.

13.2 Results for 1000 ppm of catalyst

Table S9 Results for RCM of **25** in presence of **Ru1**, **Ru11**–**Ru15** (1000 ppm)

Time [min]	Catalyst					
	**Ru1**	**Ru11**	**Ru12**	**Ru13**	**Ru14**	**Ru15**
0	0	0	0	0	0	0
1	13.7	37.5	31.7	6.0	0.6	0.1
2	42.7	73.3	73.0	17.9	4.8	0.3
3	77.7	91.7	94.2	37.1	11.1	1.2
4	93.9	97.9	99.0	61.6	19.4	1.3
5	98.2	99.4	99.7	82.0	29.3	1.2
7	99.8	99.8	99.8	96.7	50.9	1.6
10	100	99.9	99.9	99.6	77.3	2.5
15	100	99.9	99.9	100	95.4	3.0
20	100	99.9	99.9	100	98.9	4.0
25	100	99.9	99.9	100	99.7	6.3
30	100	99.9	99.9	100	99.8	8.3
40	100	99.9	99.9	100	99.9	12.5
50	100	99.9	99.9	100	99.9	15.9
60	100	99.9	100	100	99.9	23.9
90	100	100	100	100	100	43.2
120	100	100	100	100	100	63.4
1440	100	100	100	100	100	100

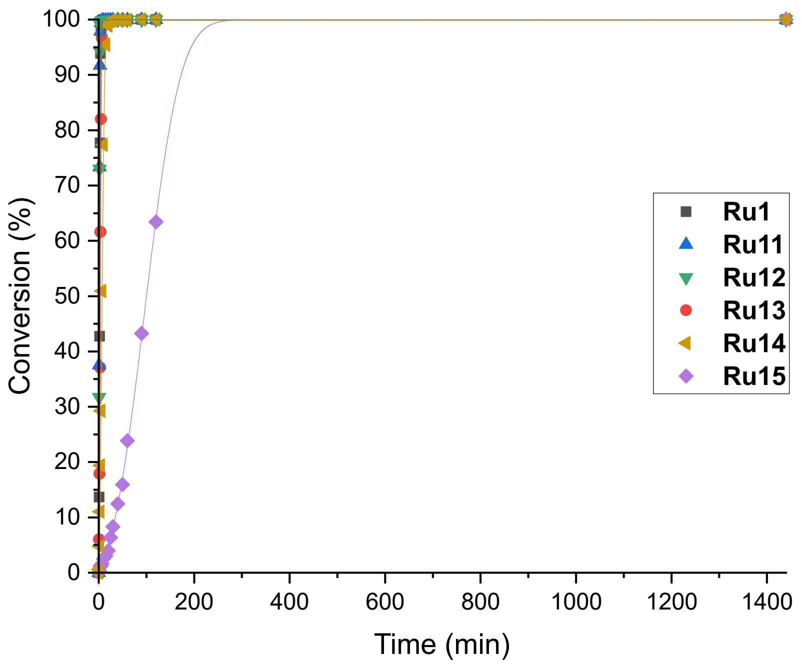

Figure S2 Plot with results for RCM of **25** (1000 ppm) and trendlines

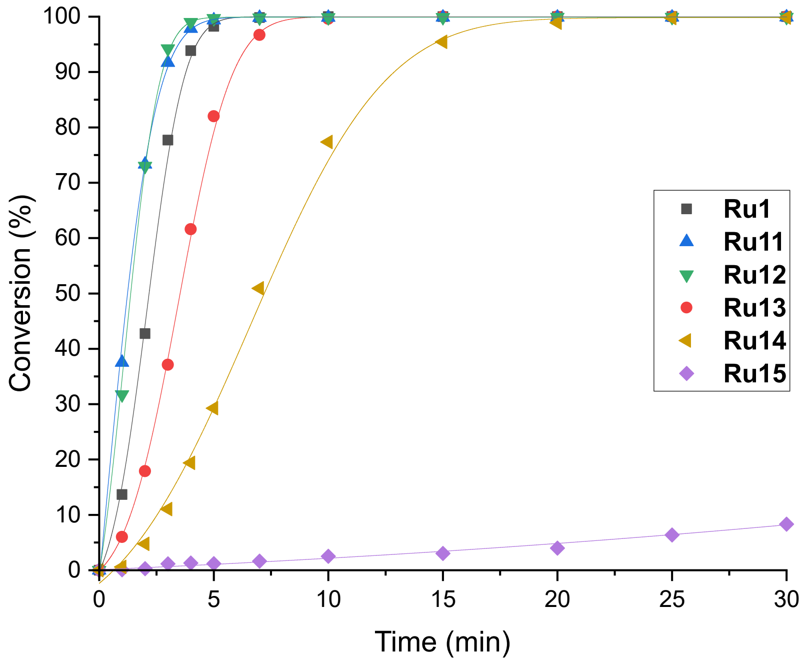

Figure S3 Plot with results for RCM of **25** (1000 ppm) and trendlines (first 30 min only).

13.3 Results for 100 ppm of catalyst

Table S10 Results for RCM of **25** in presence of **Ru1**, **Ru11**–**Ru15** (100 ppm)

Time [min]	Catalyst					
	**Ru1**	**Ru11**	**Ru12**	**Ru13**	**Ru14**	**Ru15**
0	0	0	0	0	0	0
1	0.9	10.5	3.4	0.4	0	0
2	3.6	19.7	8.1	1.2	0	0
3	8.1	31.9	15.5	3.5	0.2	0
5	20.8	54.7	31.0	8.7	1.1	0
7	35.7	66.5	43.0	14.4	1.7	0
10	57.9	79.7	58.8	25.7	3.3	0
15	84.8	92.4	78.9	50.0	5.5	0
30	100	97.6	100	92.9	16.9	0
60	100	100	100	100	56.0	0.6
120	100	100	100	100	82.2	2.1

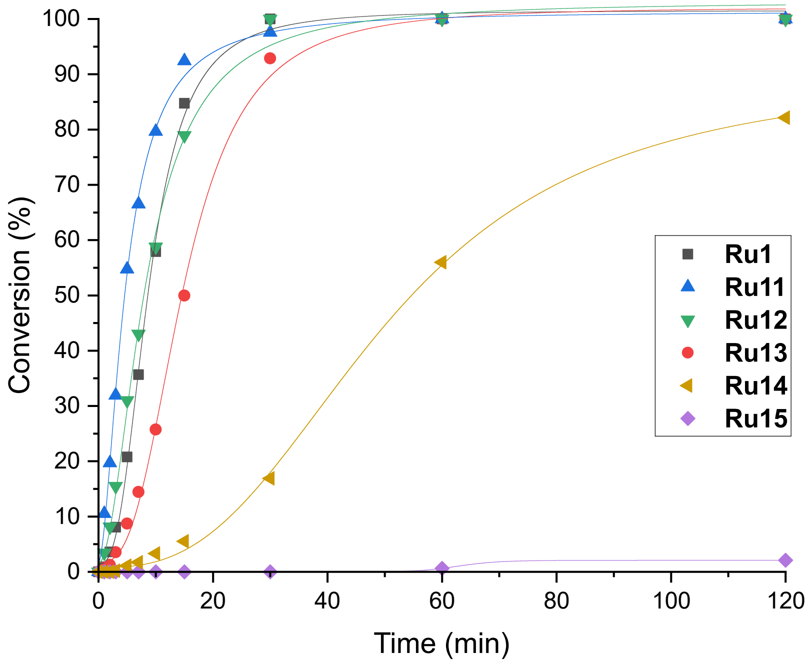

Figure S4 Plot with results for RCM of **25** (100 ppm) and trendlines

14 RCM of *N*-allyl-4-methyl-*N*-(2-methylallyl)benzenesulfonamide (27)

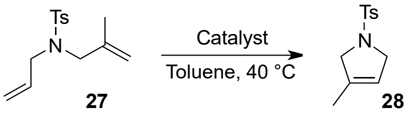

14.1 General procedure for RCM of **27**

N-allyl-4-methyl-*N*-(2-methylallyl)benzenesulfonamide (**27**) was transferred under protective argon atmosphere to Schlenk flask (1 equiv.) and then mixed with internal standard (1,3,5-methoxybenzene, 1 equiv.). The mixture was degassed and dissolved in anh. toluene (0.1 M). Stock solution of substrate and internal standard was transferred to twelve 4 mL vials. Under protective argon atmosphere complex (**Ru1**, **Ru11**–**15**, ca. 5 mg) was placed in another Schlenk flasks and dissolved in 2 mL of anh. toluene. Appropriate amount of stock solution of catalyst (1000 or 100 ppm) was added under argon to mixture of substrate and internal standard. The mixture was stirred at 40 °C for 24 h. During reaction time, samples for GC analyses were taken (ca. 0.3 mL each) to 1.5 mL vials containing ca. 1 mL of toluene and two drops of solution of SnatchCat in DCM.

14.2 Results for 1000 ppm of catalyst

Table S11 Results for RCM of **27** in presence of **Ru1**, **Ru11**–**Ru15** (1000 ppm)

Time [min]	Catalyst					
	**Ru1**	**Ru11**	**Ru12**	**Ru13**	**Ru14**	**Ru15**
0	0	0	0	0	0	0
5	71.9	92.4	97.4	16.6	42.3	—[a]
10	96.0	99.1	100	66.6	81.5	6.4
15	98.8	99.8	100	89.9	94.3	6.4
30	100	100	100	98.8	99.6	6.0
60	100	100	100	100	100	5.8
90	100	100	100	100	100	6.2
120	100	100	100	100	100	5.9
1440	100	100	100	100	100	100

[a] The measurement was omitted due to random deviation.

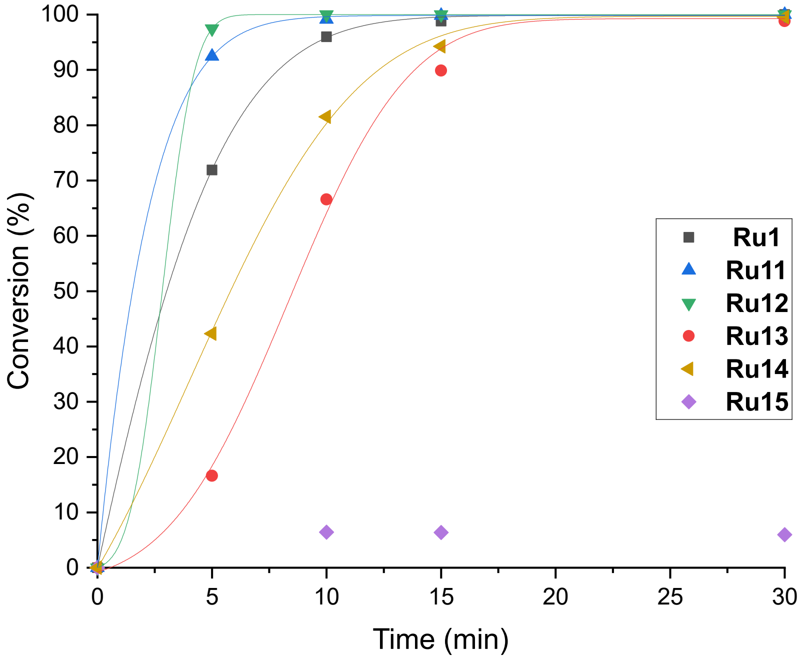

Figure S5 Plot with results for RCM of **27** (1000 ppm) and trendlines (first 30 min only)

14.3 Results for 100 ppm of catalyst

Table S12 Results for RCM of **27** in presence of **Ru1**, **Ru11**–**Ru15** (100 ppm)

Time [min]	Catalyst					
	**Ru1**	**Ru11**	**Ru12**	**Ru13**	**Ru14**	**Ru15**
0	0	0	0	0	0	0
5	10.2	49.8	31.7	2.9	7.1	—[a]
10	12.0	79.2	69.4	3.0	8.2	0.8
15	15.9	89.7	86.0	5.5	9.7	—[a]
30	41.6	96.4	96.5	19.3	14.7	3.9
60	84.4	98.6	99.0	78.8	31.0	4.9
90	93.4	98.9	99.4	92.6	39.6	—[a]
120	96.0	99.0	99.6	96.7	47.2	—[a]
1440	99.3	99.0	99.6	99.7	84.9	25.2

[a] The measurement was omitted due to random deviation.

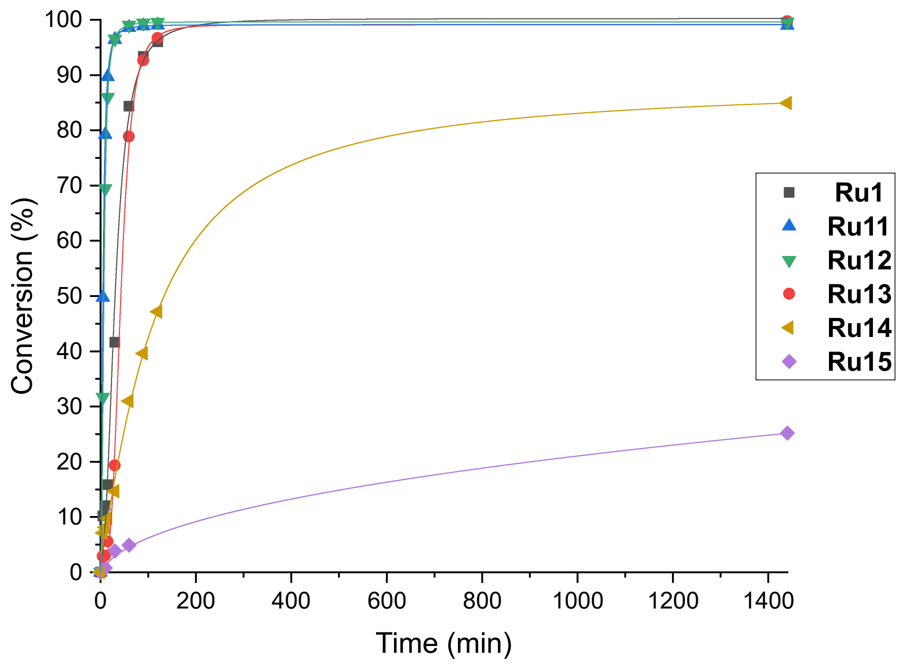

Figure S8 Plot with results for RCM of **27** (100 ppm) and trendlines

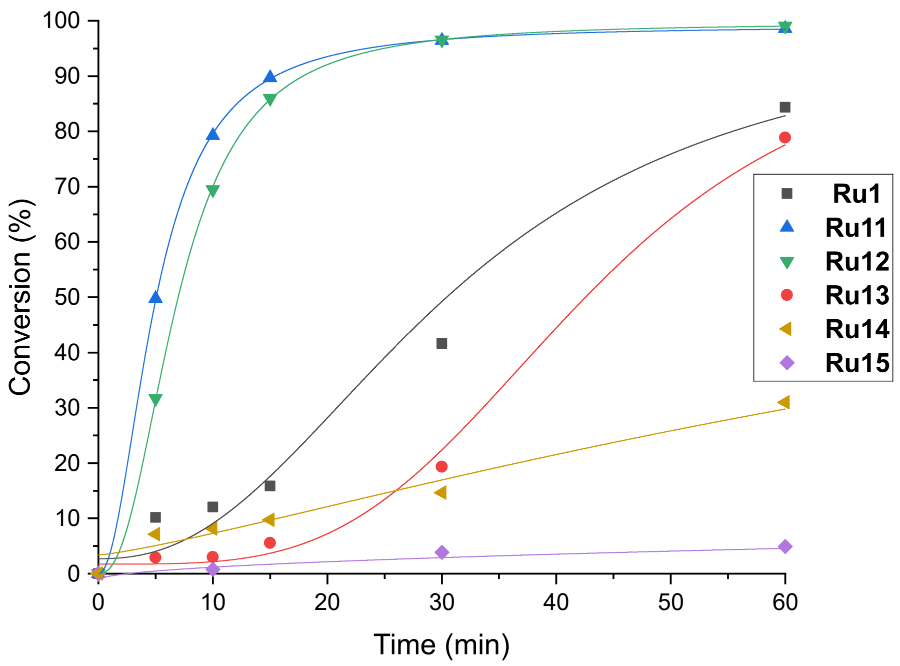

Figure S9 Plot with results for RCM of **27** (100 ppm) and trendlines (first 60 min only)

15 RCM of *N,N*-diallyl-4-ethoxy-3-(1-methyl-7-oxo-3-propyl-6,7-dihydro-1*H*-pyrazolo[4,3-*d*]pyrimidin-5-yl)benzenesulfonamide (29)

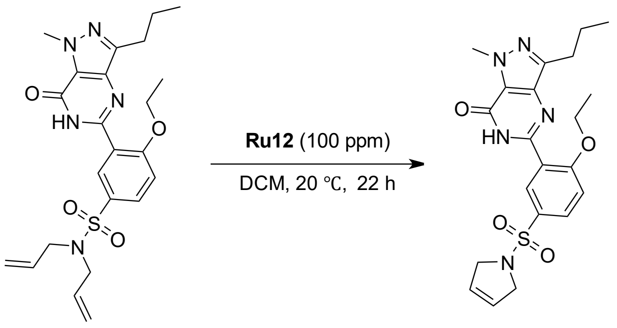

29 **30**

In Schlenk flask **29** (145 mg, 304 µmol, 1 equiv.) was dissolved in anhydrous DCM (3 mL) under argon atmosphere and then solution of complex **Ru12** in anh. DCM (1722 µmol/L) was added. The resulting solution was stirred at room temperature for 22 hours. After reaction completion, to reaction mixture ca. 10 mL of n-hexane was added and concentrated to 3 mL. Supernatant was discarded. Product was dried under vacuum overnight to furnish the product **30** as a white solid (124 mg, 279 µmol, 92%).

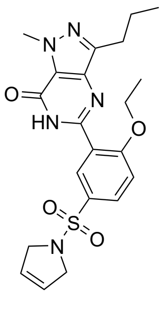

^{1}H NMR (400 MHz, Chloroform-*d*) δ 10.83 (s, 1H), 8.88 (d, *J* = 2.5 Hz, 1H), 7.91 (dd, *J* = 2.5, 8.7 Hz, 1H), 7.15 (d, *J* = 8.8 Hz, 1H), 5.72 – 5.65 (m, 2H), 4.36 (q, *J* = 7.0 Hz, 2H), 4.27 (s, 3H), 4.19 (s, 4H), 2.93 (t, *J* = 7.6 Hz, 2H), 1.86 (h, *J* = 7.4 Hz, 2H), 1.63 (t, *J* = 7.0 Hz, 3H), 1.02 (t, *J* = 7.4 Hz, 3H).

^{1}H NMR spectrum is in agreement with previously reported.[7]

16 Stability test for Ru11 and Ru15 on air

Based on recent publication published by Mauduit et al.,[8] we decided to do stability test on air in the same way.

To NMR tube complex **Ru11** or **Ru15** (0.024 M), 1,3,5trimethoxybenzene and toluene-d_8 were added on air. The tube was equipped with a rubber cap sealed with Parafilm®. The resulting solution was held at 80 °C and monitored by ^{1}H NMR over time.

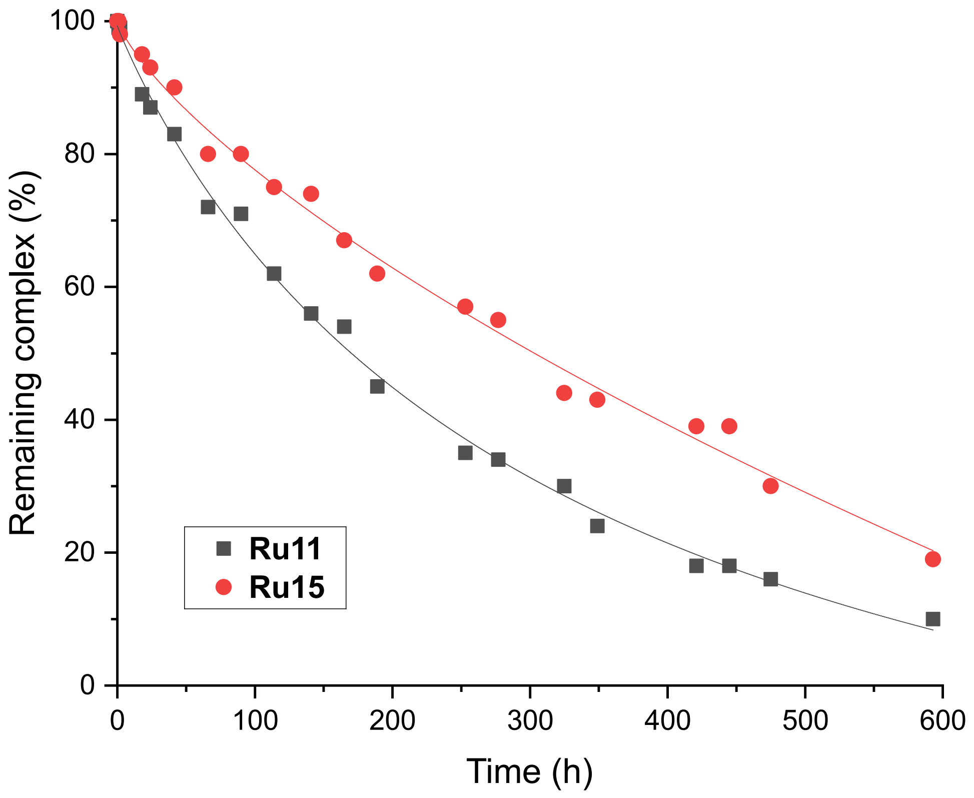

Table S13 Results for stability test on air in toluene for **Ru11** and **Ru15**

Time [min]	Remaining complex [%]	
	**Ru11**	**Ru15**
0	100	100
1	99	101
2	100	98
18	89	95
24	87	93
41.5	83	90
66	72	80
90	71	80
114	62	75
141	56	74
165	54	67
189	45	62
253	35	57
277	34	55
325	30	44
349	24	43
421	18	39
445	18	39
475	16	30
593	10	19

17 XRD DATA

17.1 Crystal data and structure refinement details for **Ru1** and **Ru12**

**Identification code**	**Ru6**	**Ru12**
Empirical formula	$C_{33}H_{41}Cl_2NORu$	$C_{37}H49Cl_2NORu$
Formula weight	639.64	695.74
Temperature/K	100(2)	100(2)
Crystal system	monoclinic	monoclinic
Space group	$P2_1/c$	$P2_1/c$
a/Å	16.01368(15)	14.97514(11)
b/Å	10.34404(7)	11.89935(9)
c/Å	19.37205(17)	19.33172(15)
α/°	90	90
β/°	110.8702(10)	98.9295(7)
γ/°	90	90
Volume/Å^3	2998.37(5)	3403.05(5)
Z	4	4
ρ_{calc}g/cm^3	1.417	1.358
μ/mm^{-1}	6.063	5.385
F(000)	1328.0	1456.0
Crystal size/mm^3	0.18 × 0.08 × 0.06	0.417 × 0.21 × 0.177
Radiation	Cu$K\alpha$ (λ = 1.54184)	CuKα (λ = 1.54184)
2Θ range for data collection/°	9.448 to 134.15	8.756 to 134.148
Index ranges	$-19 \le h \le 19$, $-12 \le k \le 12$, $-23 \le l \le 23$	$-17 \le h \le 17$, $-14 \le k \le 14$, $-23 \le l \le 23$
Reflections collected	51271	33773
Independent reflections	5349 [R_{int} = 0.0523, R_{sigma} = 0.0247]	6071 [R_{int} = 0.0457, R_{sigma} = 0.0286]
Data/restraints/parameters	5349/0/351	6071/0/388
Goodness-of-fit on F^2	1.040	1.032
Final R indexes [I>=2σ (I)]	R_1 = 0.0212, wR_2 = 0.0529	R_1 = 0.0302, wR_2 = 0.0762
Final R indexes [all data]	R_1 = 0.0234, wR_2 = 0.0539	R_1 = 0.0313, wR_2 = 0.0770
Largest diff. peak/hole/e Å^{-3}	0.32/-0.34	0.99/-0.71

17.2 Bond lengths for **Ru12**

Atom	Atom	Length/Å	Atom	Atom	Length/Å
C(1)	C(2)	1.561(3)	C(19)	C(20)	1.404(3)
C(1)	N(1)	1.325(3)	C(19)	C(24)	1.518(3)
C(1)	Ru(1)	1.936(2)	C(20)	C(21)	1.380(4)
C(2)	C(3)	1.540(3)	C(21)	C(22)	1.383(4)
C(2)	C(5)	1.536(3)	C(22)	C(23)	1.394(4)
C(2)	C(15)	1.548(3)	C(23)	C(26)	1.516(3)
C(3)	C(4)	1.531(3)	C(24)	C(25)	1.540(3)
C(4)	C(16)	1.528(3)	C(26)	C(27)	1.509(4)
C(4)	C(17)	1.530(3)	C(28)	C(29)	1.446(3)
C(4)	N(1)	1.526(3)	C(28)	Ru(1)	1.840(2)
C(5)	C(6)	1.398(3)	C(29)	C(30)	1.401(3)
C(5)	C(10)	1.386(4)	C(29)	C(34)	1.409(3)
C(6)	C(7)	1.391(4)	C(30)	C(31)	1.386(3)
C(7)	C(8)	1.386(4)	C(31)	C(32)	1.390(4)
C(8)	C(9)	1.392(4)	C(32)	C(33)	1.399(4)
C(8)	C(11)	1.511(4)	C(33)	C(34)	1.389(3)
C(9)	C(10)	1.390(4)	C(34)	O(1)	1.366(3)
C(11)	C(12)	1.537(4)	C(35)	C(36)	1.486(4)
C(12)	C(13)	1.521(4)	C(35)	C(37)	1.519(4)
C(12)	C(14)	1.531(5)	C(35)	O(1)	1.480(3)
C(18)	C(19)	1.399(3)	Cl(1)	Ru(1)	2.3342(6)
C(18)	C(23)	1.412(3)	Cl(2)	Ru(1)	2.3543(6)
C(18)	N(1)	1.453(3)	O(1)	Ru(1)	2.3192(15)

17.3 X-Ray structure for **Ru12**

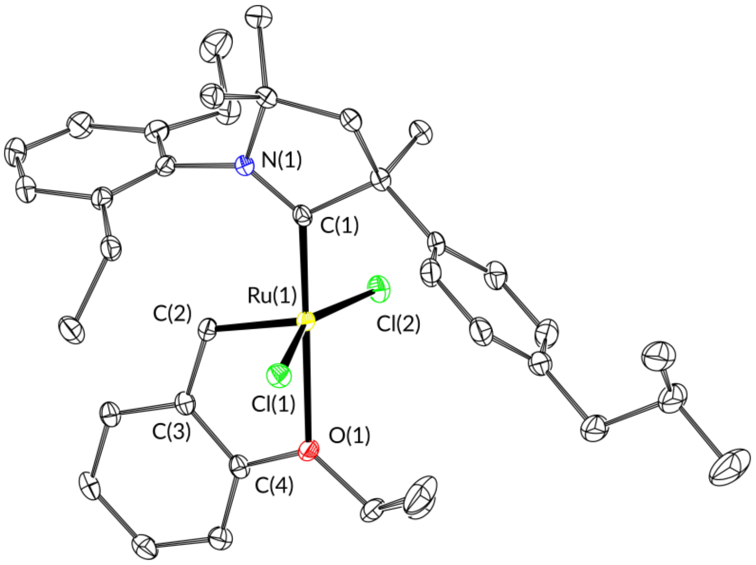

18 Copies of NMR spectra

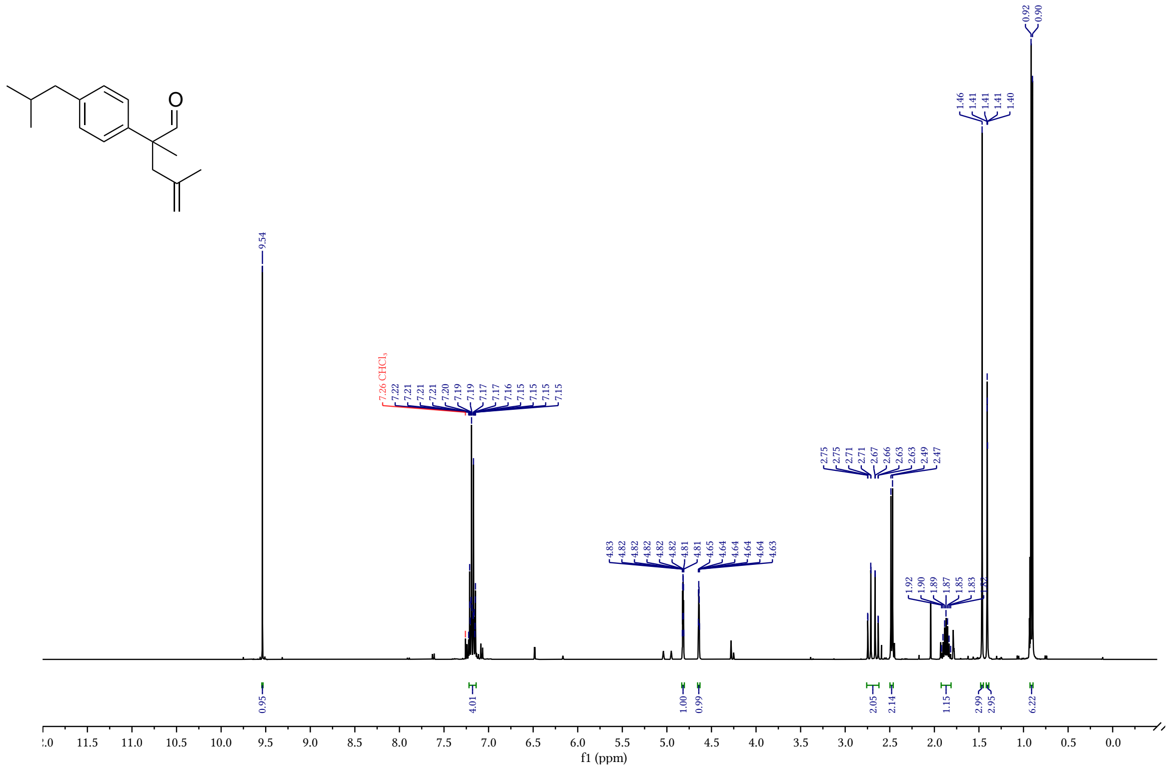

Figure S6 ^{1}H NMR spectrum of 5

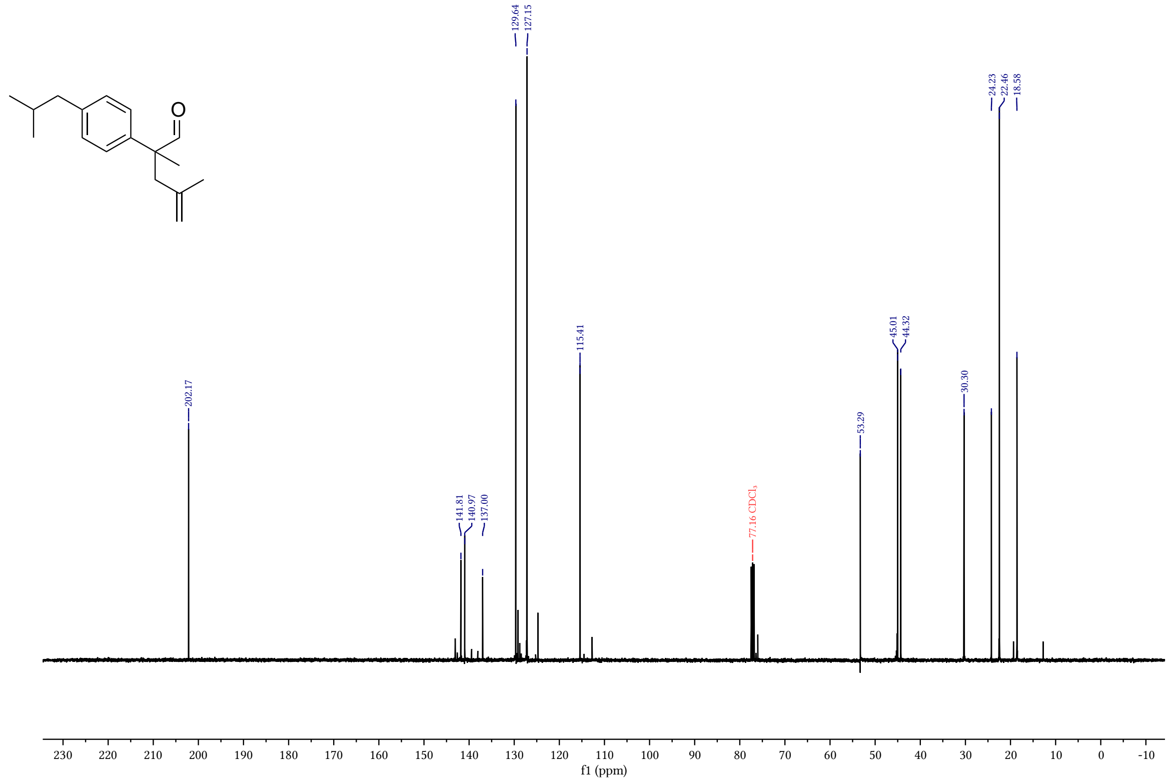

Figure S7 ^{13}C NMR spectrum of **5**

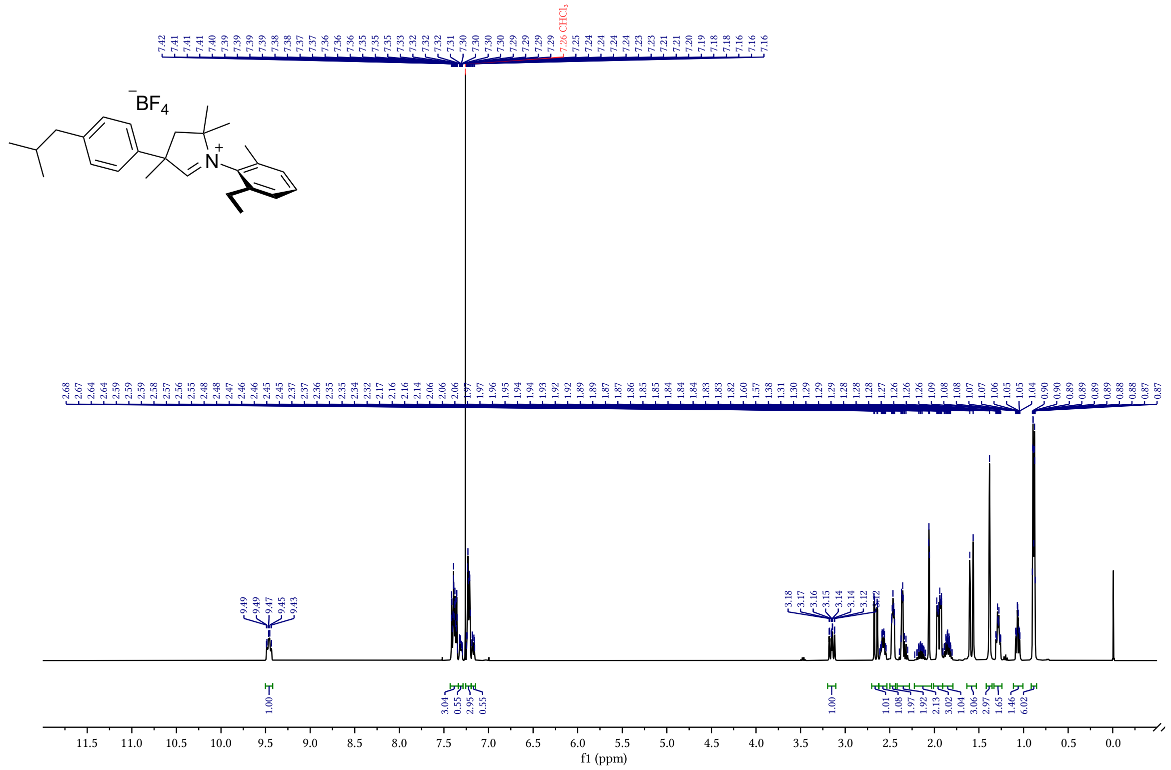

Figure S8 ^{1}H NMR spectrum of **8a**

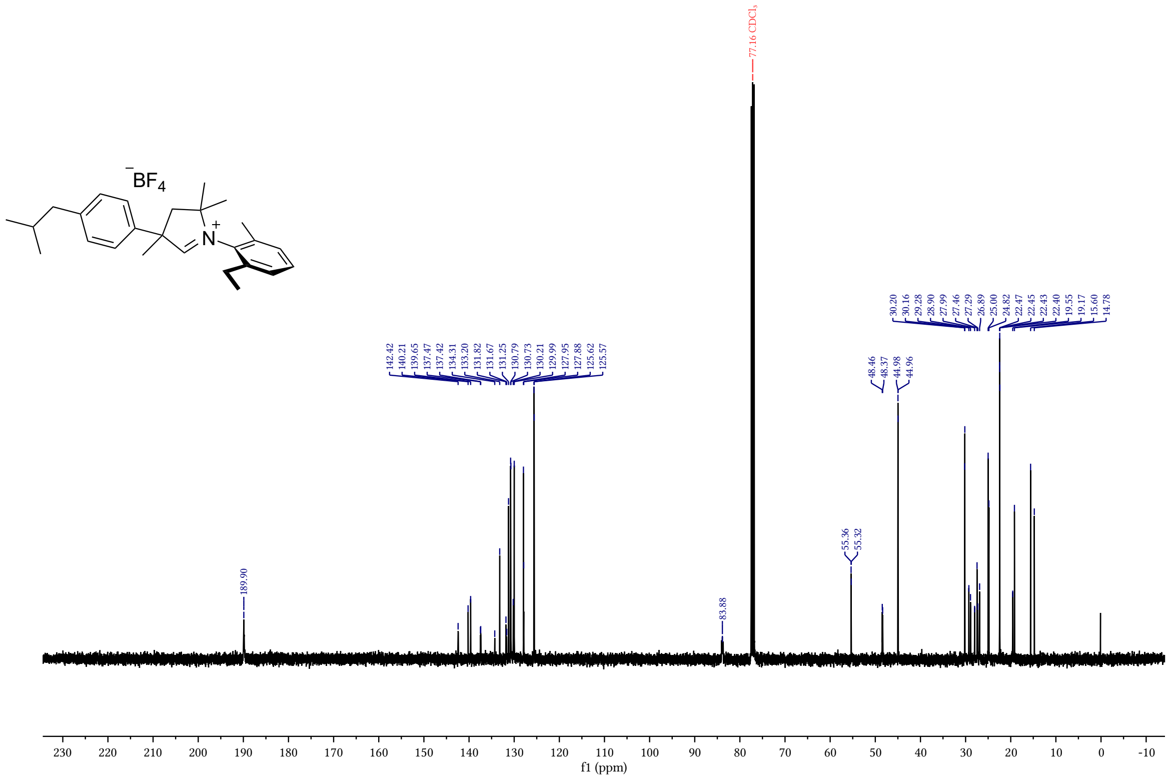

Figure S9 ^{13}C NMR spectrum of **8a**

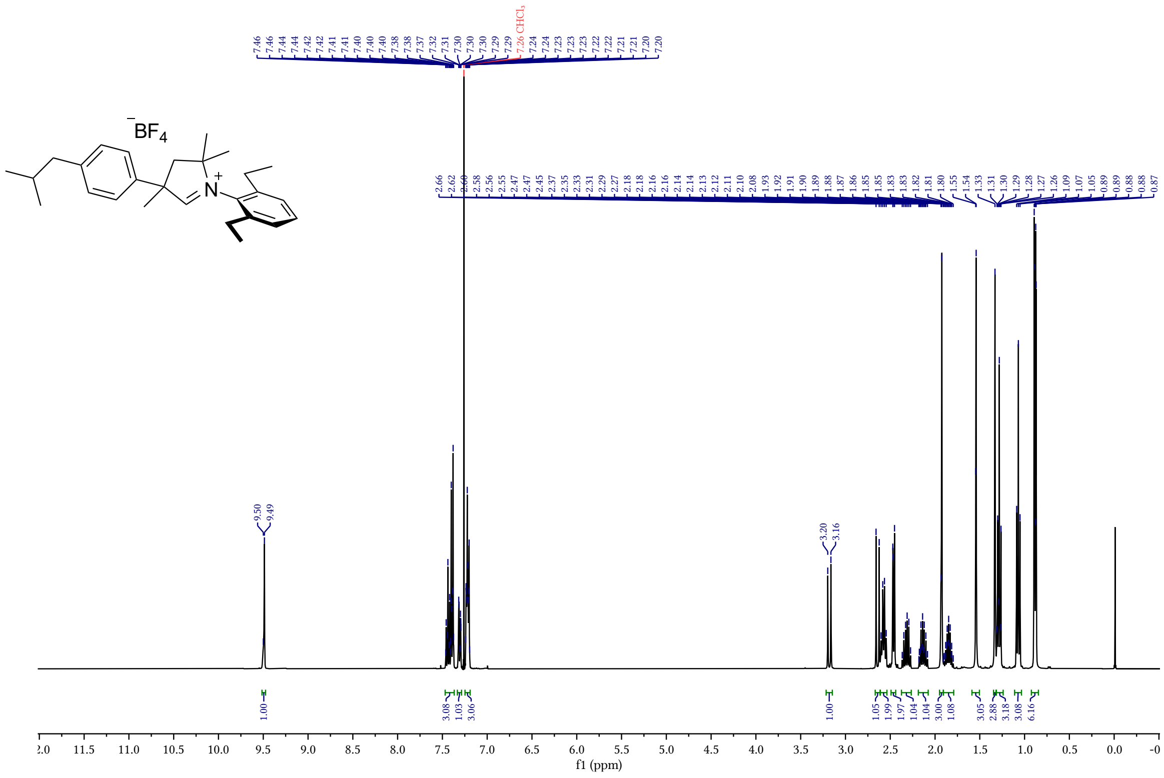

Figure S10 ^{1}H NMR spectrum of **8b**

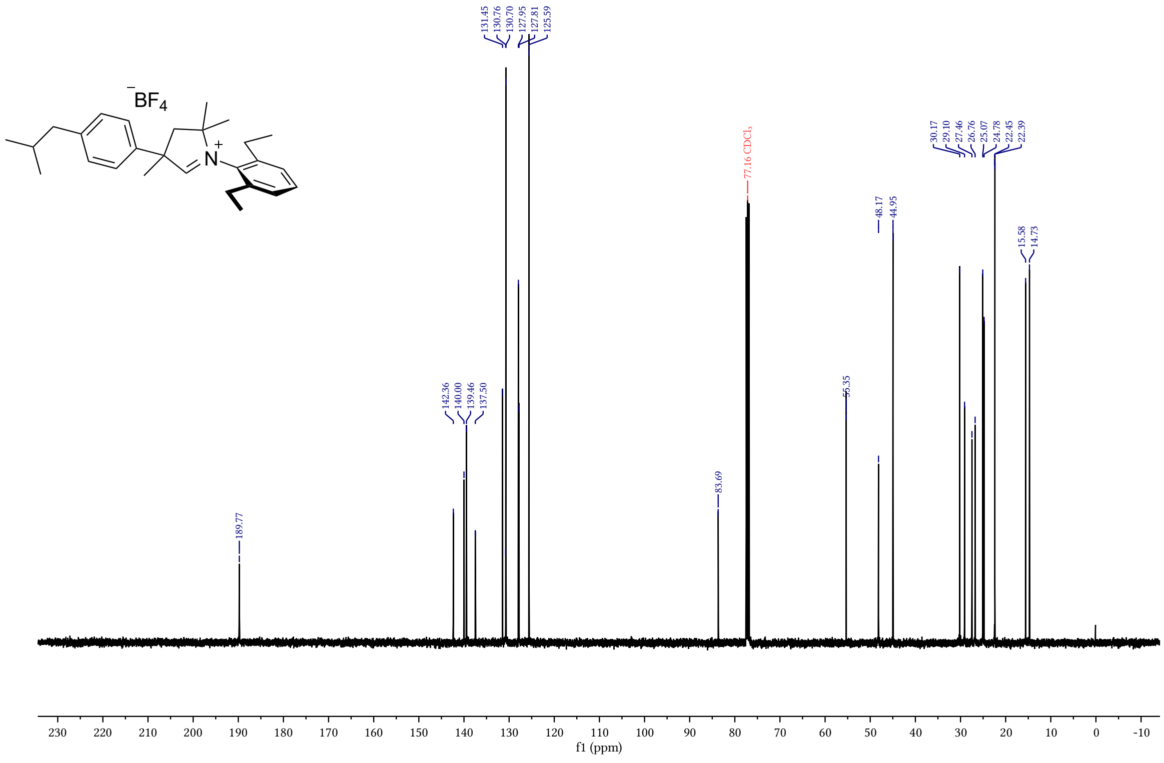

Figure S11 ^{13}C NMR spectrum of **8b**

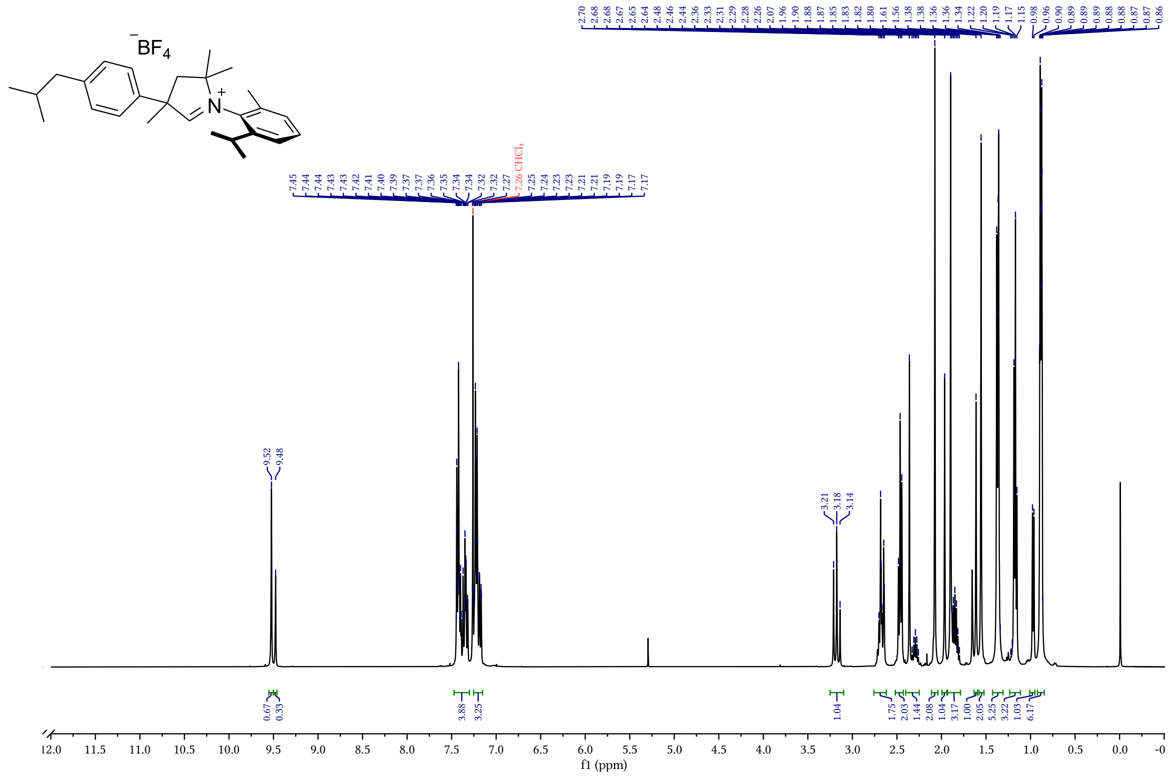

Figure S12 ^{1}H NMR spectrum of **8c**

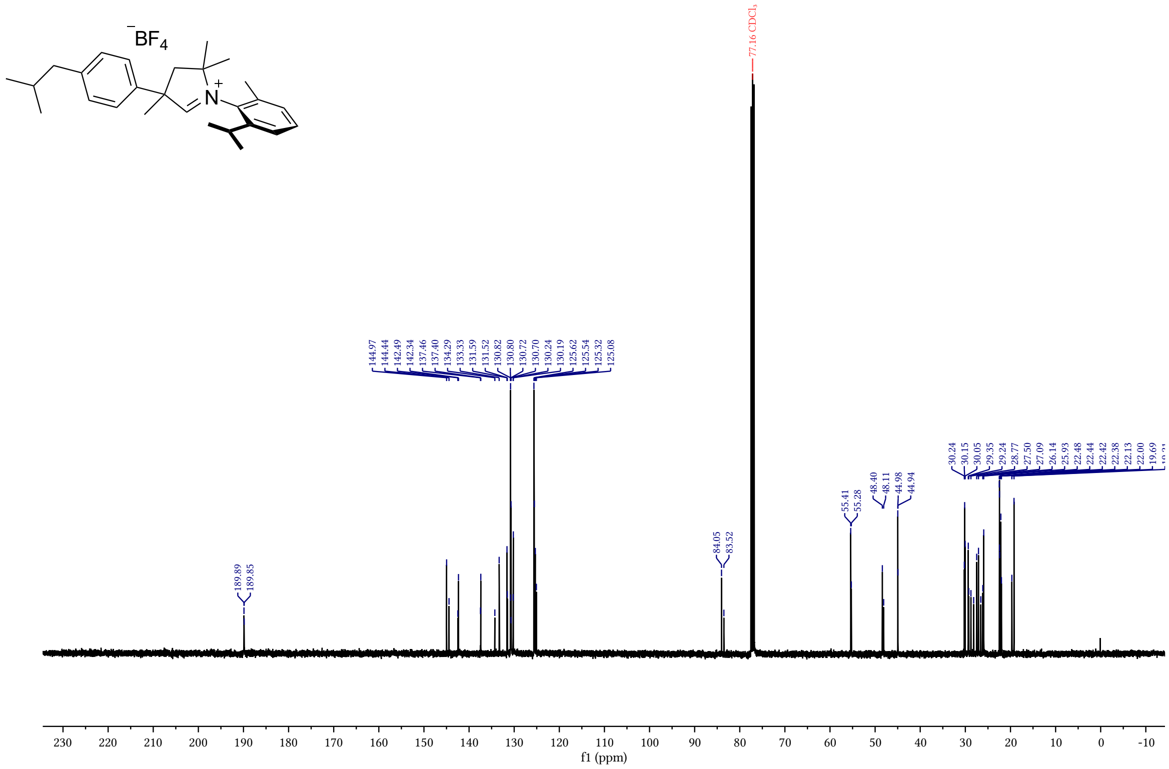

Figure S13 ^{13}C NMR spectrum of **8c**

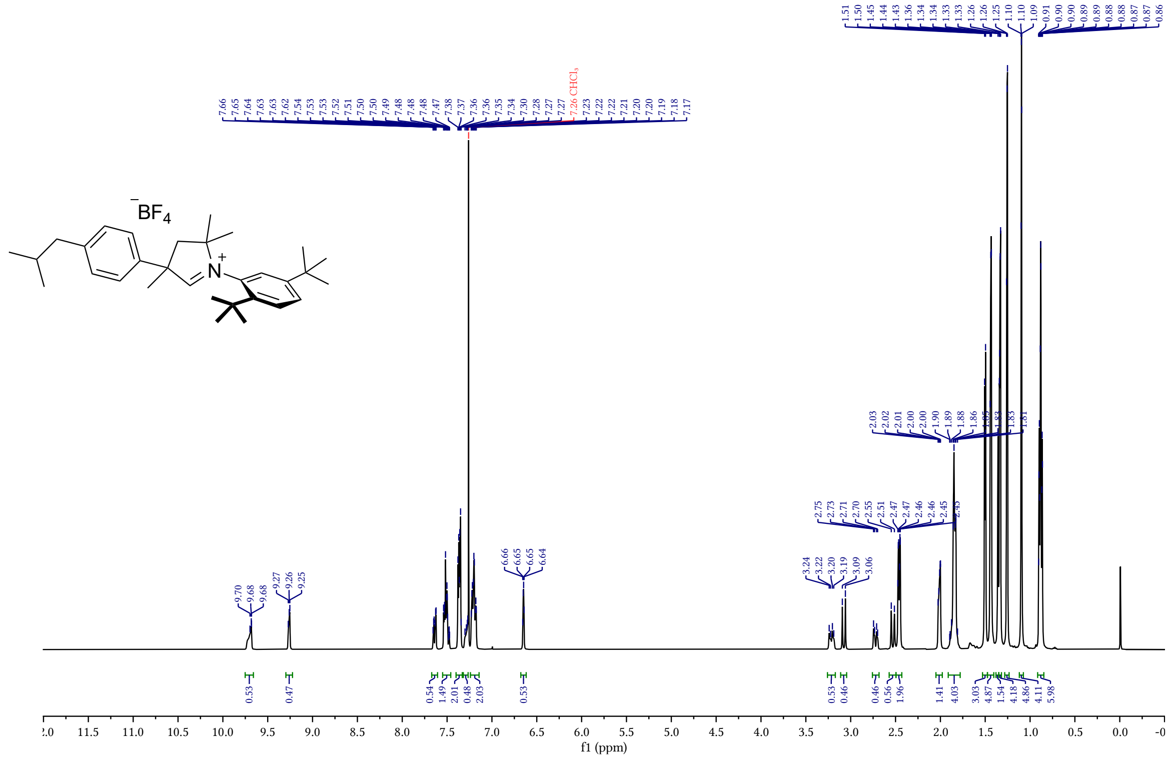

Figure S14 ^{1}H NMR spectrum of **8d**

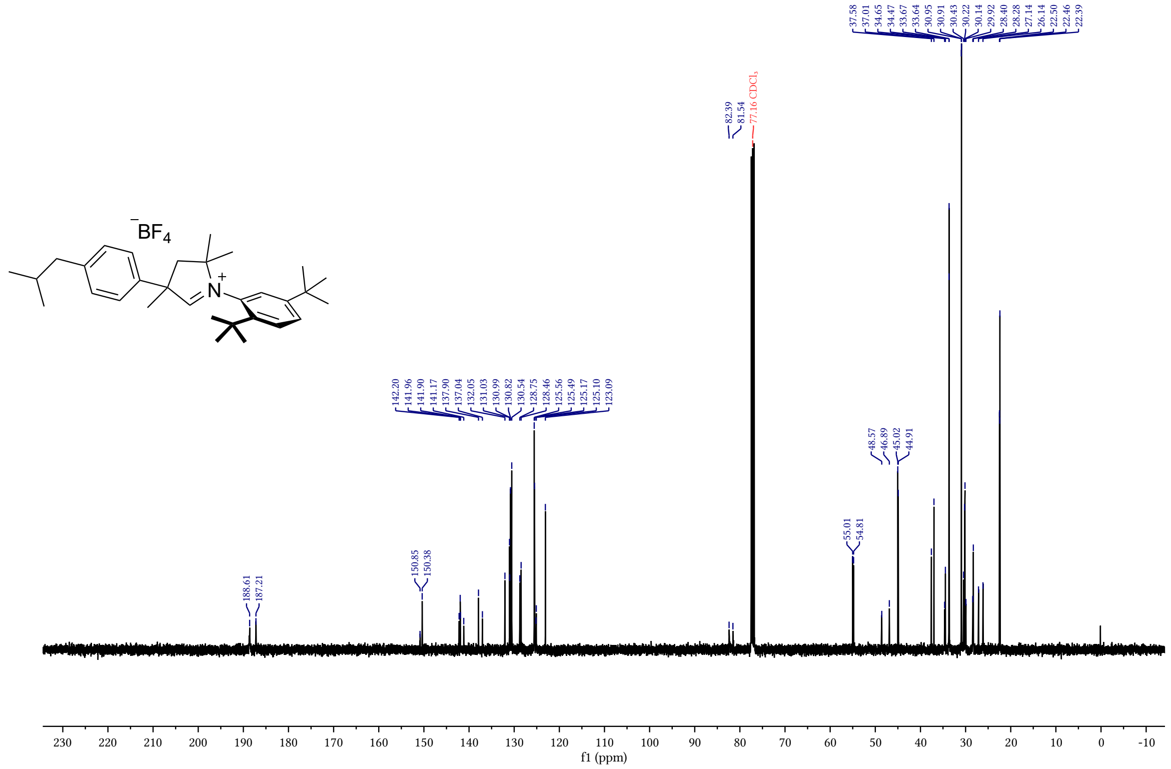

Figure S15 ^{13}C NMR spectrum of **8d**

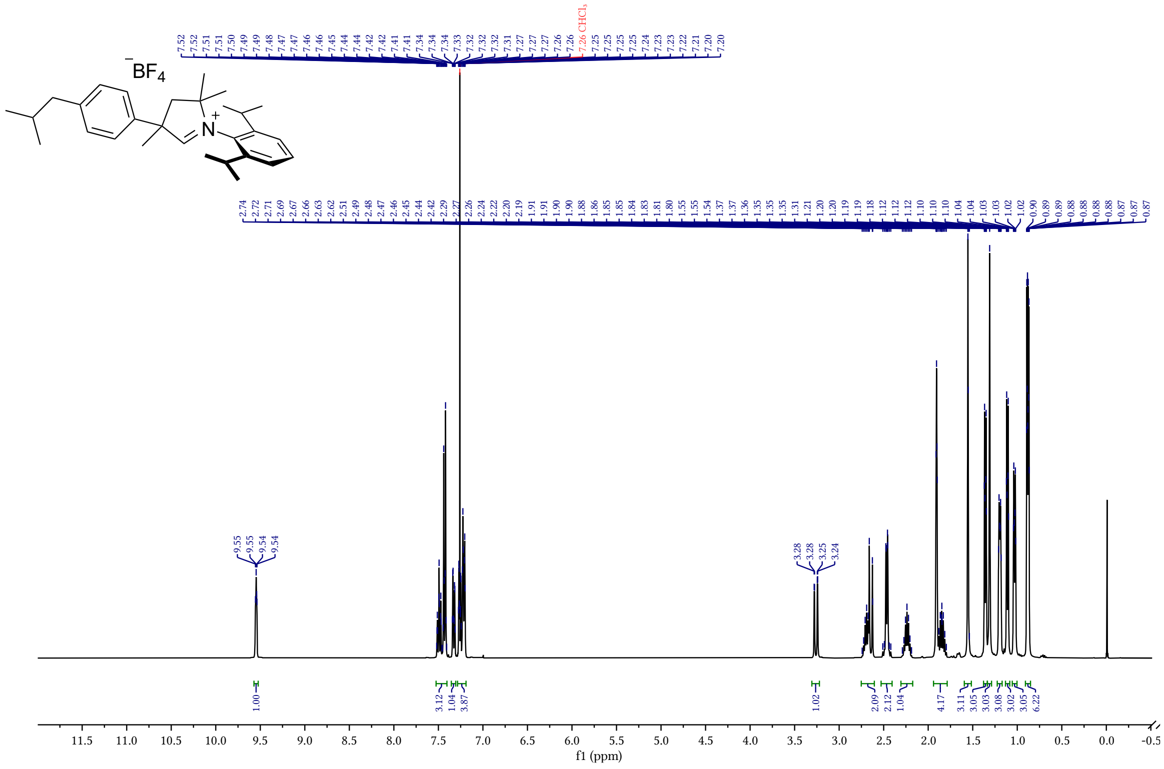

Figure S16 ^{1}H NMR spectrum of **8e**

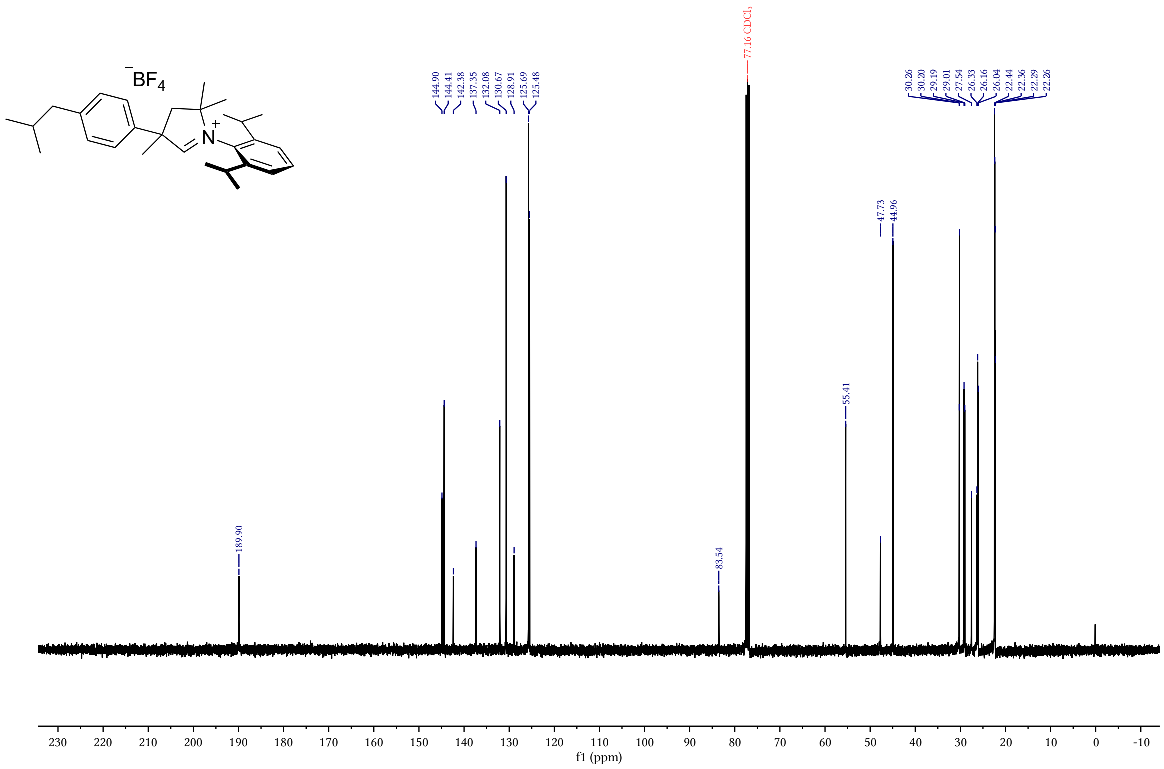

Figure S17 ^{13}C NMR spectrum of **8e**

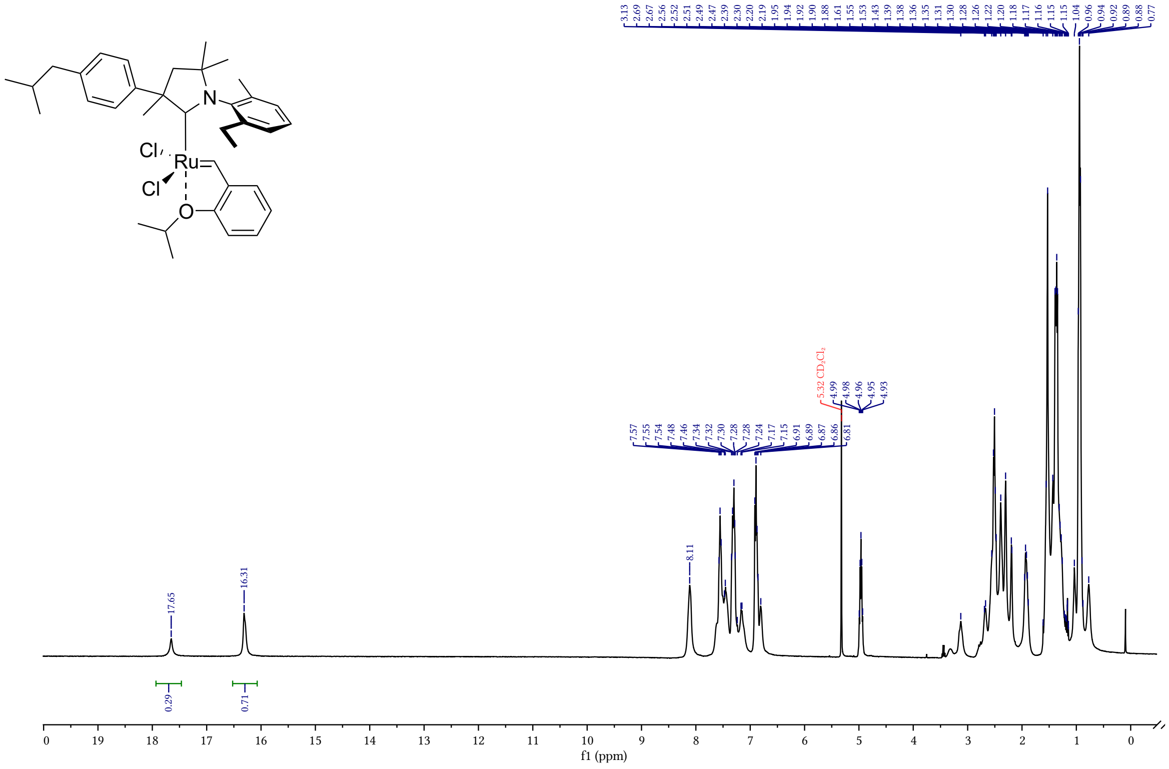

Figure S18 ^{1}H NMR spectrum of **Ru11**

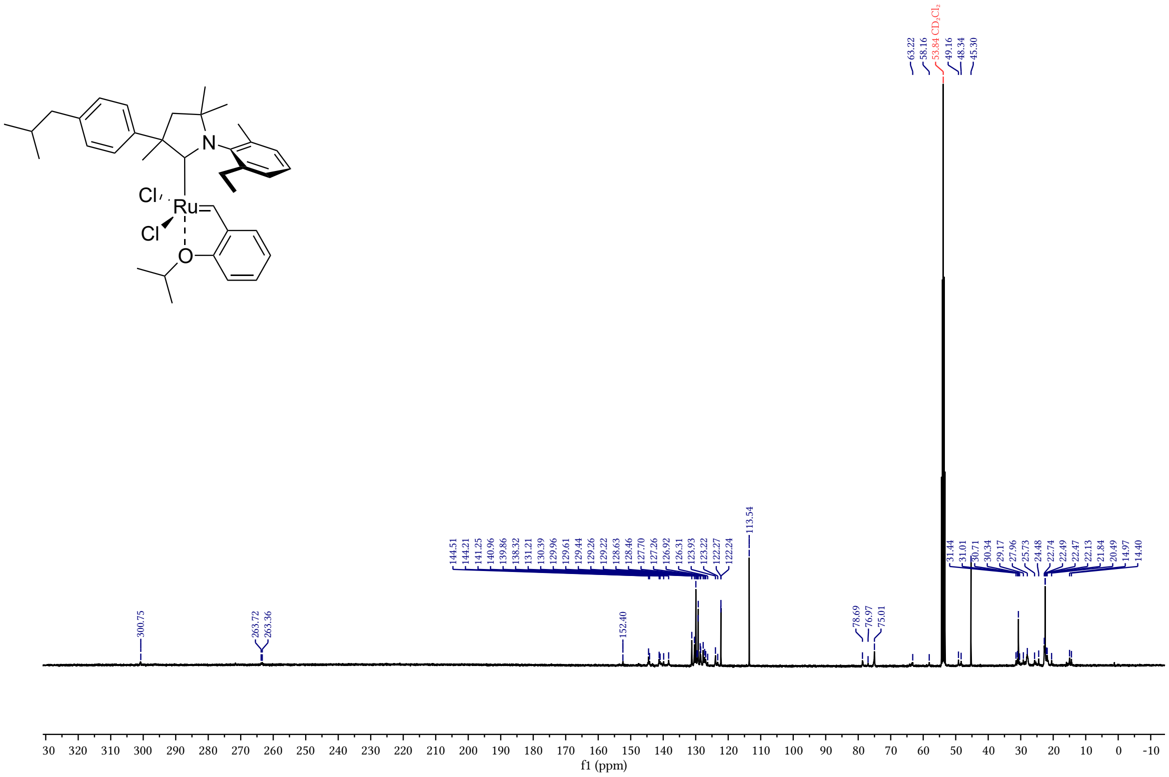

Figure S19 ^{13}C NMR spectrum of **Ru11**

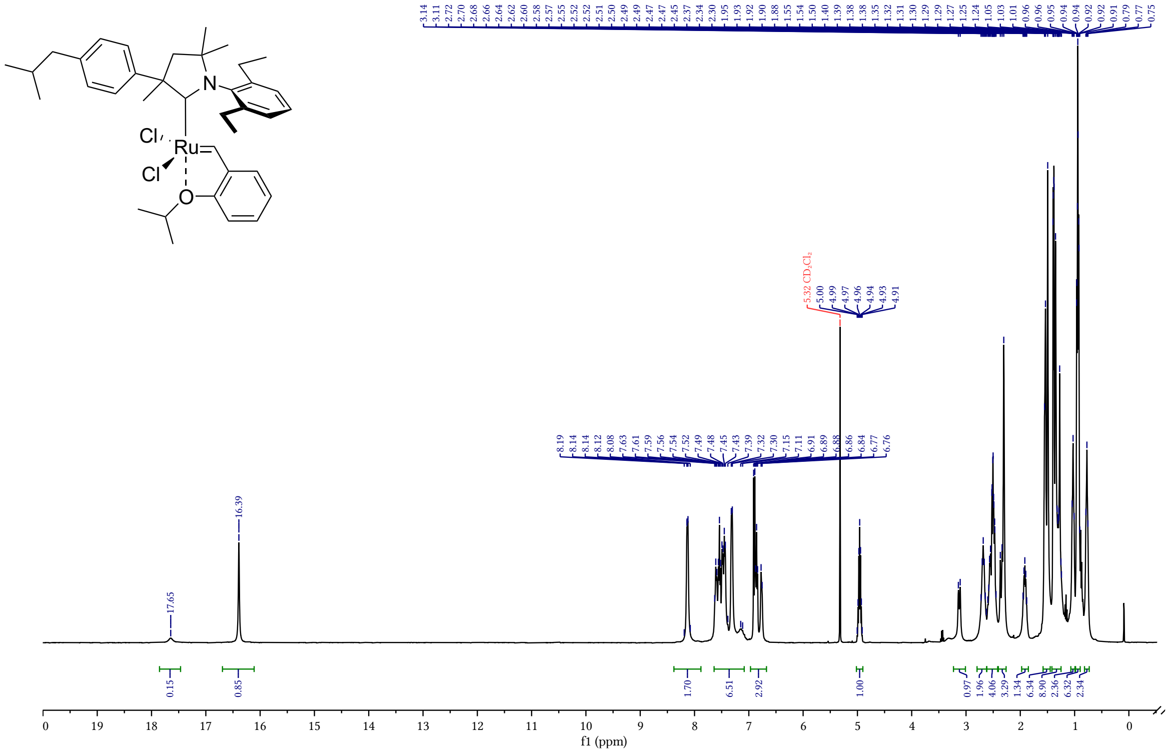

Figure S20 ^{1}H NMR spectrum of **Ru12**

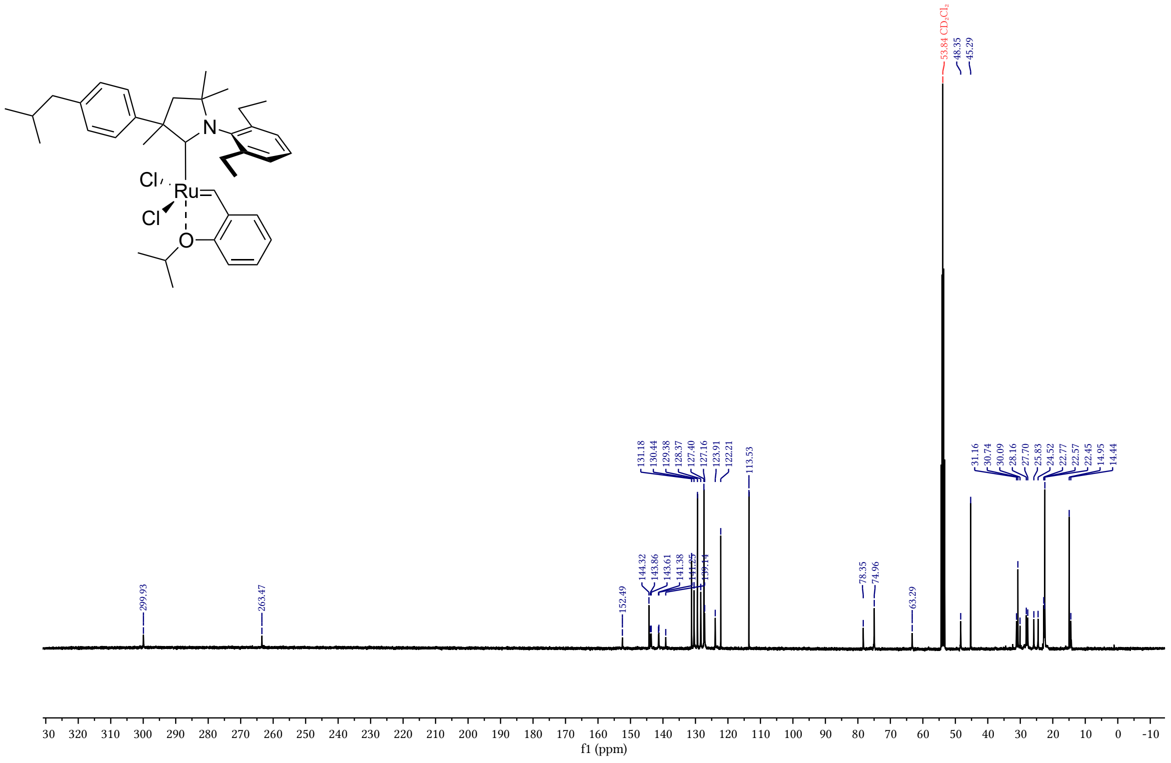

Figure S21 ^{13}C NMR spectrum of **Ru12**

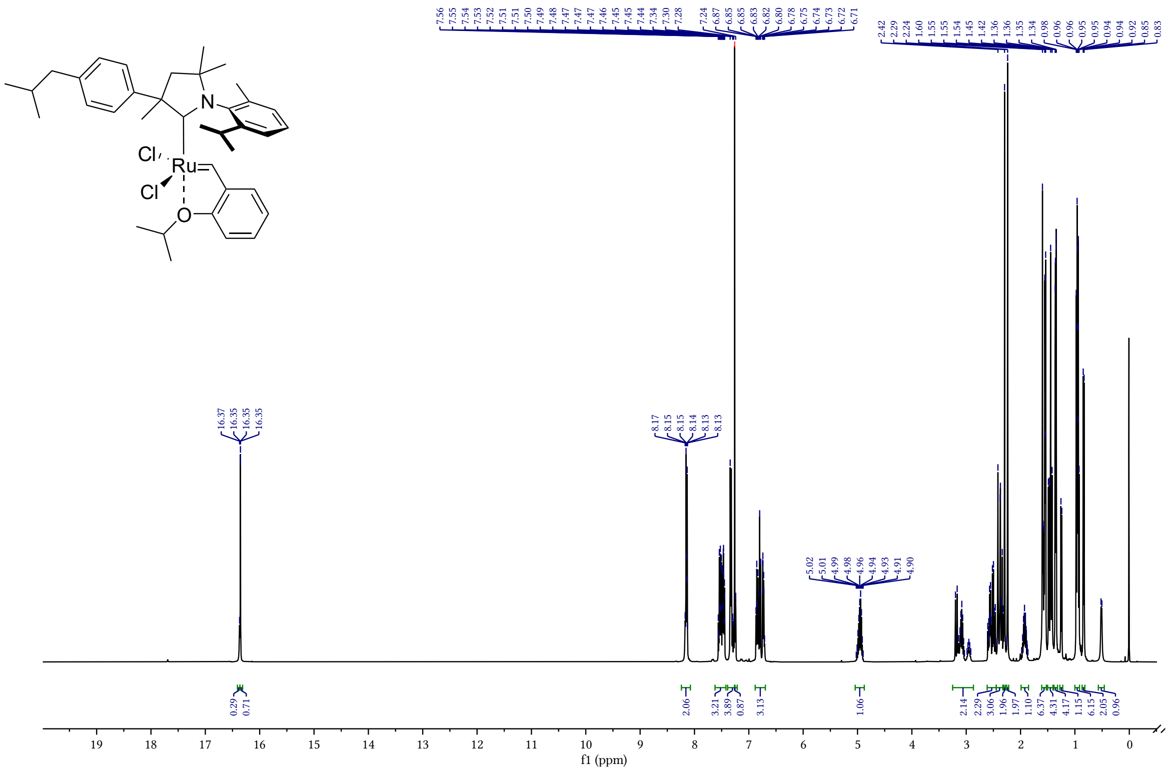

Figure S22 ^{1}H NMR spectrum of **Ru13**

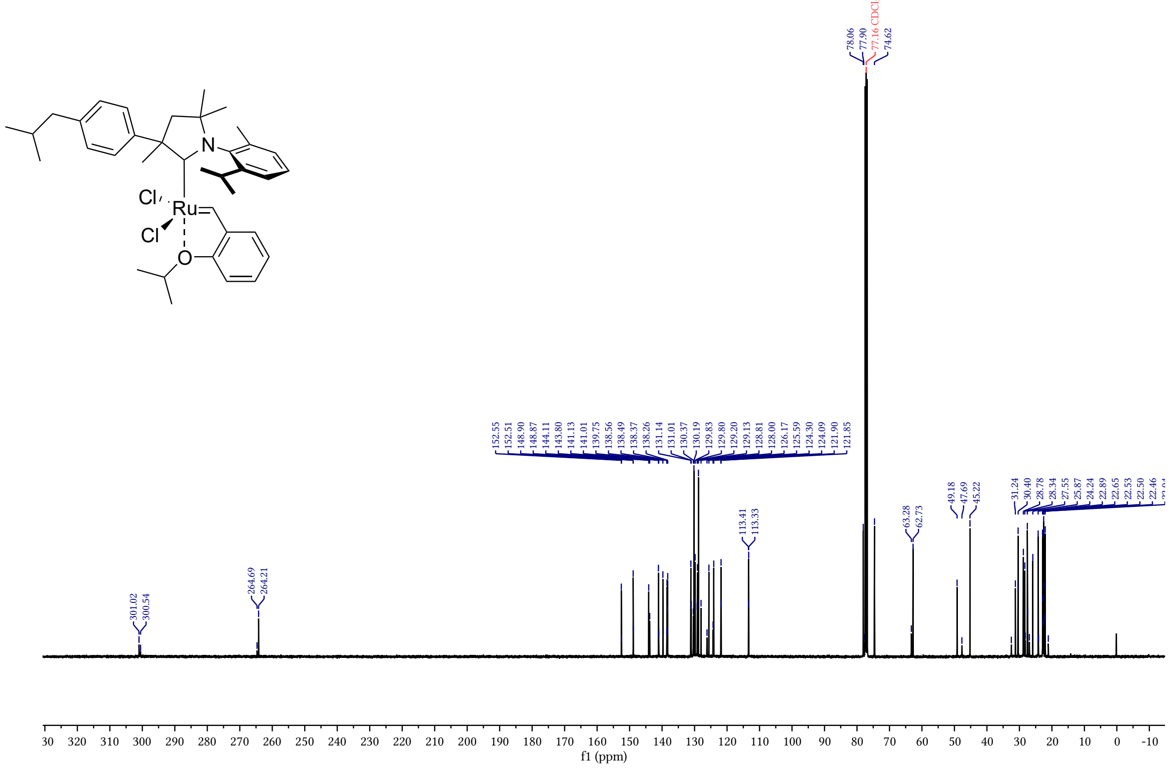

Figure S23 ^{13}C NMR spectrum of **Ru13**

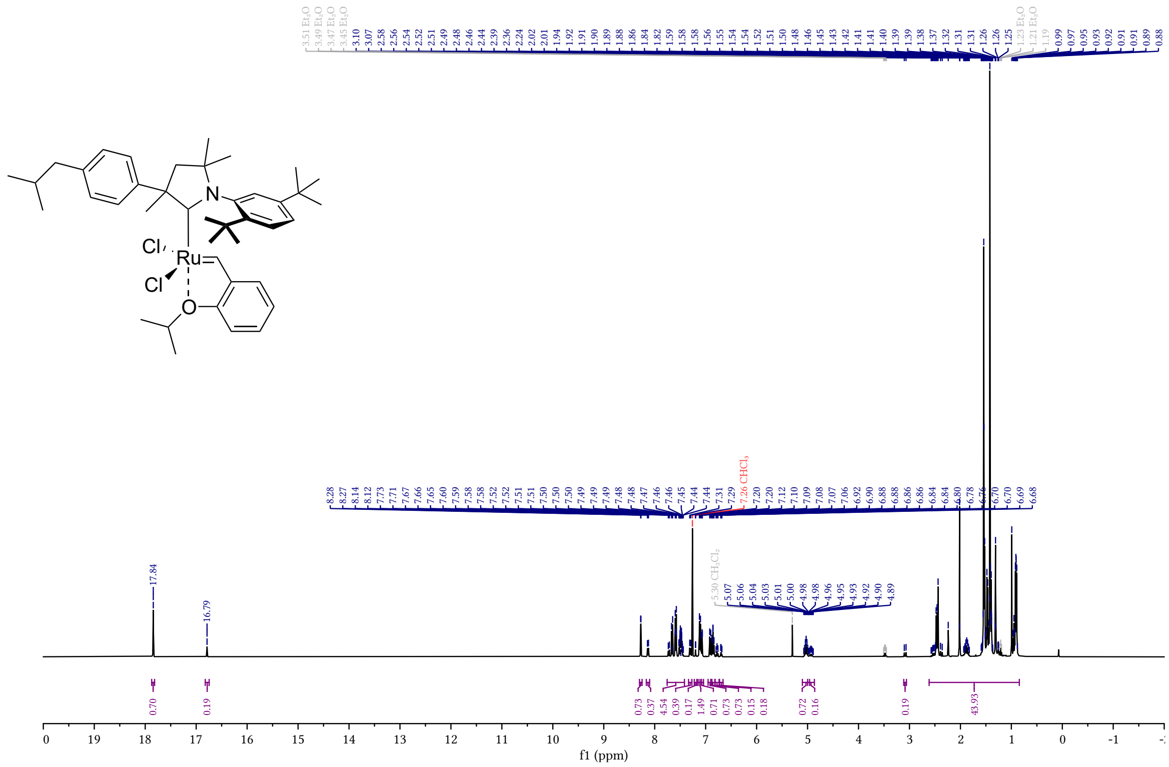

Figure S24 ^{1}H NMR spectrum of **Ru14**

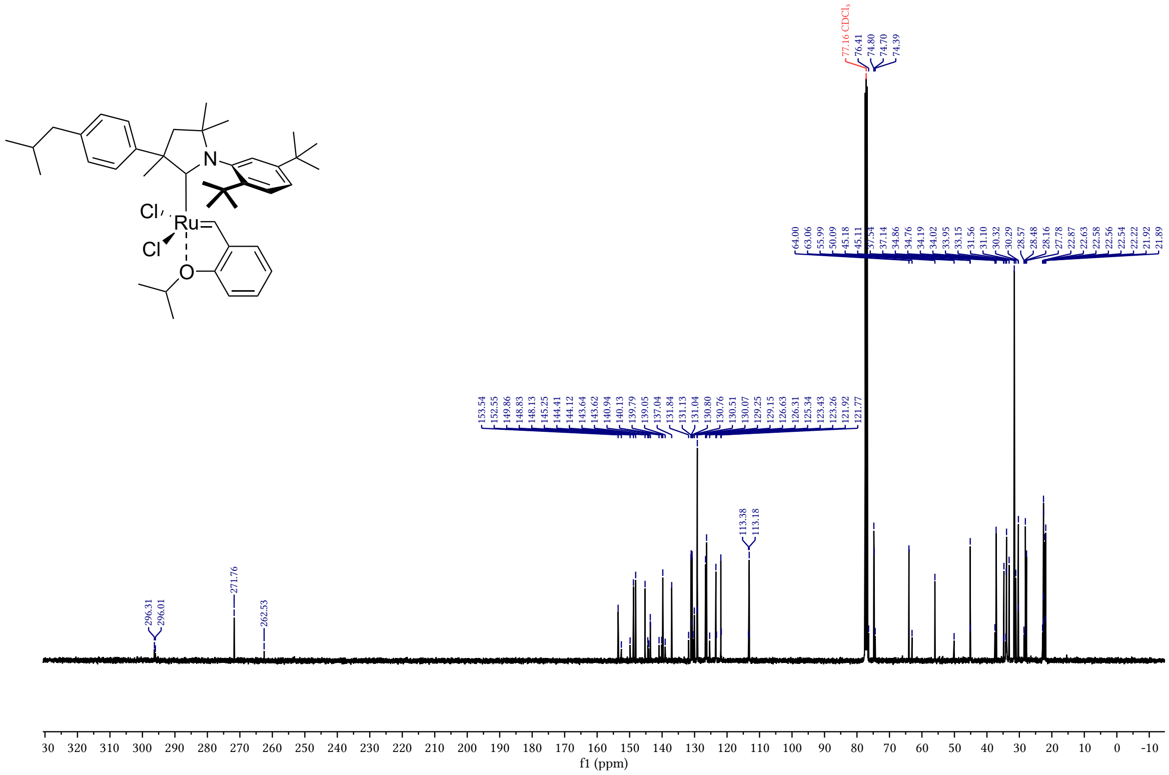

Figure S25 ^{13}C NMR spectrum of **Ru14**

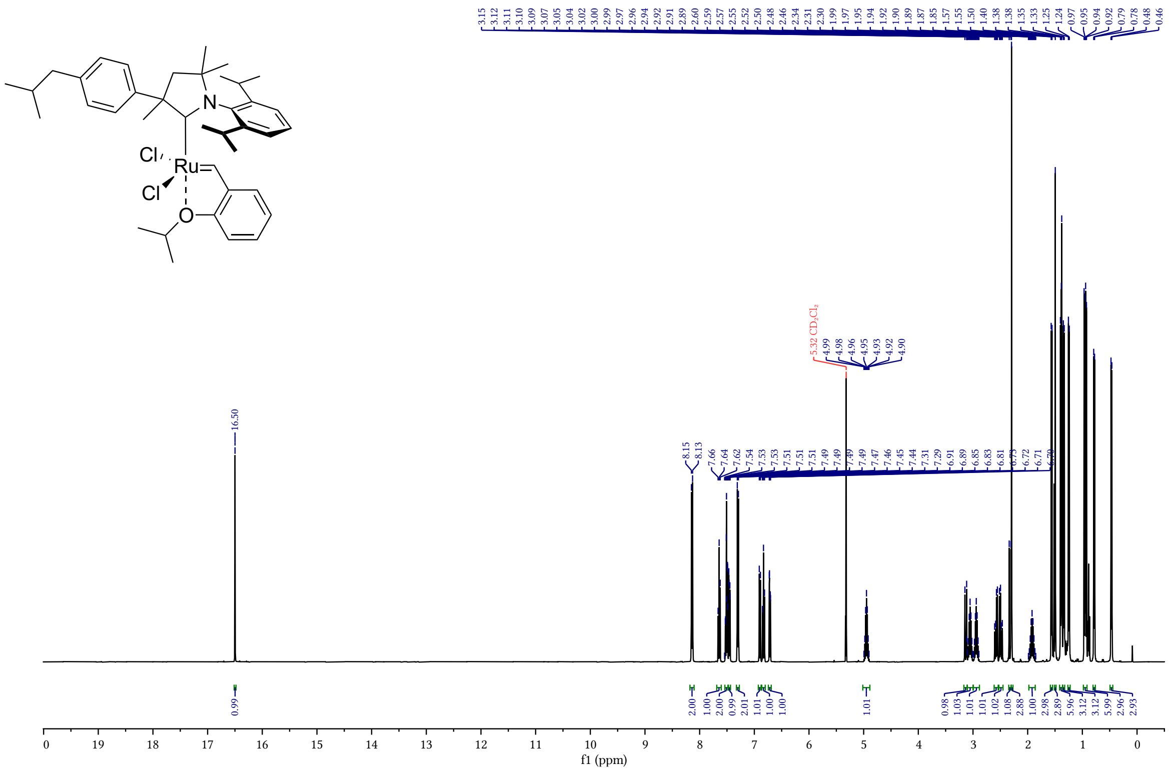

Figure S26 ^{1}H NMR spectrum of **Ru15**

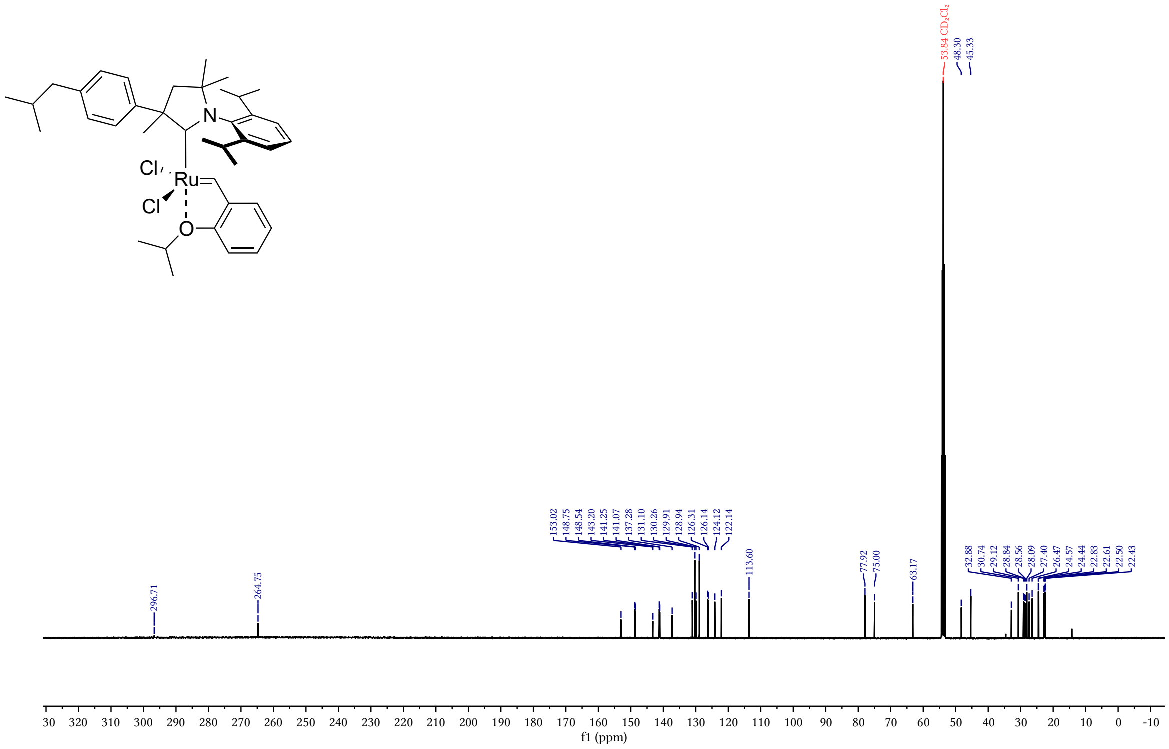

Figure S27 ^{13}C NMR spectrum of **Ru15**

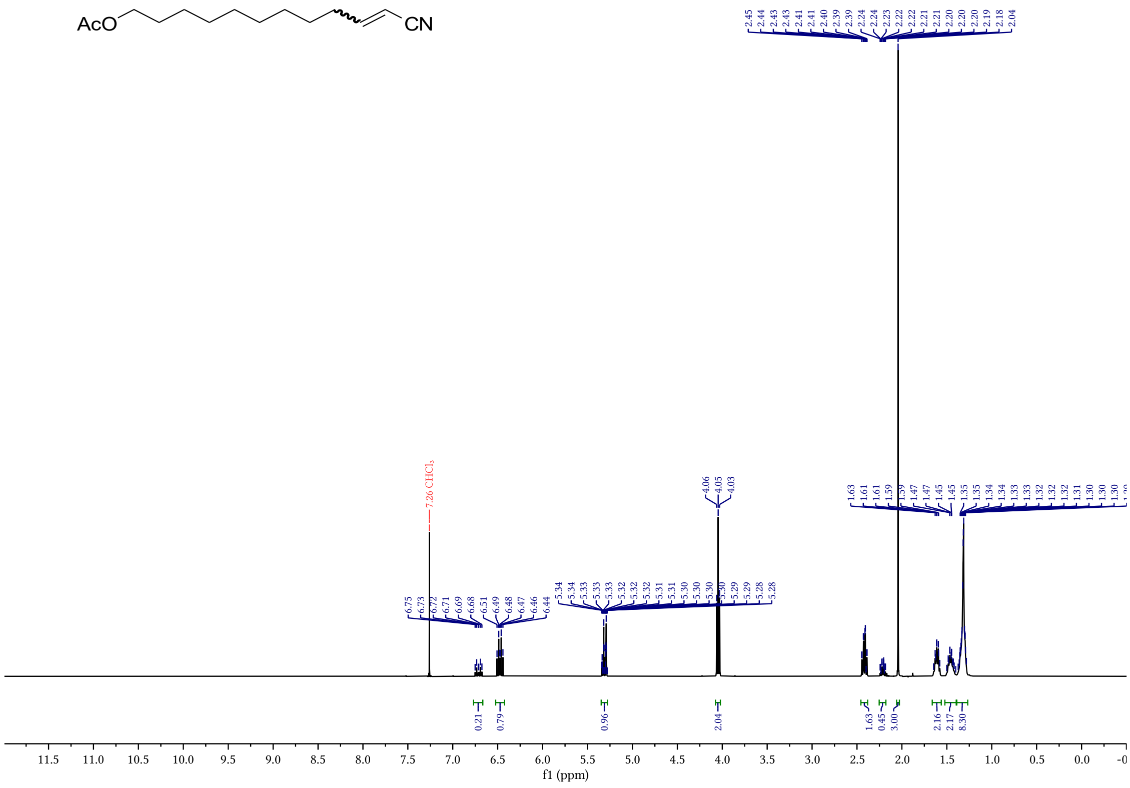

Figure S28 ^{1}H NMR spectrum of **24**

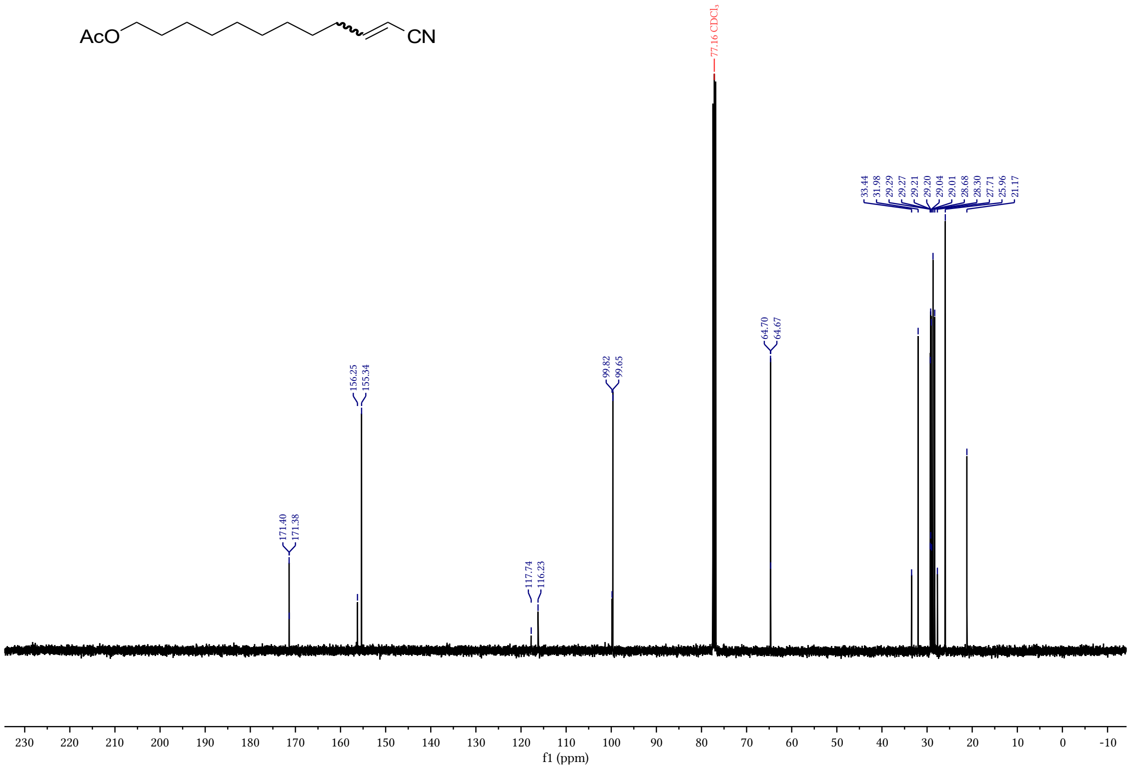

Figure S29 ^{13}C NMR spectrum of **24**

19 References

(1) Rogers, R. D.; Kore, R.; Uppara, P. V. Methods of Acylation with an Ionic Liquid Catalyzing Medium. US20180170847A1, June 21, 2018. https://patents.google.com/patent/US20180170847A1/en?oq=US2018170847 (accessed 2023-02-24).

(2) Li, H.; Hong, J. H. Synthesis and Anti-HIV Evaluation of New Acyclic Phosphonate Nucleotide Analogues and Their Bis(SATE) Derivatives. *Nucleosides, Nucleotides & Nucleic Acids* **2010**, *29* (8), 581–590. https://doi.org/10.1080/15257770.2010.495958.

(3) Schmidt, B.; Krehl, S.; Jablowski, E. Assisted Tandem Catalytic RCM-Aromatization in the Synthesis of Pyrroles and Furans. *Org. Biomol. Chem.* **2012**, *10* (26), 5119–5130. https://doi.org/10.1039/C2OB25543J.

(4) Armarego, W. L. F. Aliphatic Compounds. In *Purification of laboratory chemicals*; Butterworth-Heinemann is an imprint of Elsevier: Amsterdam, 2017; p 171.

(5) Gawin, R.; Kozakiewicz, A.; Guńka, P. A.; Dąbrowski, P.; Skowerski, K. Bis(Cyclic Alkyl Amino Carbene) Ruthenium Complexes: A Versatile, Highly Efficient Tool for Olefin Metathesis. *Angew. Chem. Int. Ed.* **2017**, *56* (4), 981–986. https://doi.org/10.1002/anie.201609009.

(6) Blackwell, H. E.; O'Leary, D. J.; Chatterjee, A. K.; Washenfelder, R. A.; Bussmann, D. A.; Grubbs, R. H. New Approaches to Olefin Cross-Metathesis. *J. Am. Chem. Soc.* **2000**, *122* (1), 58–71. https://doi.org/10.1021/ja993063u.

(7) Monsigny, L.; Piątkowski, J.; Trzybiński, D.; Woźniak, K.; Nienałtowski, T.; Kajetanowicz, A.; Grela, K. Activated Hoveyda-Grubbs Olefin Metathesis Catalysts Derived from a Large Scale Produced Pharmaceutical Intermediate – Sildenafil Aldehyde. *Adv. Synth. Catal.* **2021**, *363* (19), 4590–4604. https://doi.org/10.1002/adsc.202100669.

(8) Del Vecchio, A.; Talcik, J.; Colombel-Rouen, S.; Lorkowski, J.; Serrato, M. R.; Roisnel, T.; Vanthuyne, N.; Bertrand, G.; Jazzar, R.; Mauduit, M. Highly Robust and Efficient Blechert-Type Cyclic(Alkyl)(Amino)Carbene Ruthenium Complexes for Olefin Metathesis. *ACS Catal.* **2023**, *13* (9), 6195–6202. https://doi.org/10.1021/acscatal.3c01208.
